# E6AP is essential for the proliferation of HPV-positive cancer cells by preventing senescence

**DOI:** 10.1371/journal.ppat.1012914

**Published:** 2025-02-07

**Authors:** Alicia Avenhaus, Milica Velimirović, Julia Bulkescher, Martin Scheffner, Felix Hoppe-Seyler, Karin Hoppe-Seyler

**Affiliations:** 1 German Cancer Research Center (DKFZ), Molecular Therapy of Virus-Associated Cancers, Heidelberg, Germany; 2 Faculty of Biosciences, Heidelberg University, Heidelberg, Germany; 3 Department of Biology, Konstanz Research School Chemical Biology, University of Konstanz, Konstanz, Germany; University of North Carolina at Chapel Hill, UNITED STATES OF AMERICA

## Abstract

Oncogenic types of human papillomaviruses (HPVs) are major human carcinogens. The formation of a trimeric complex between the HPV E6 oncoprotein, the cellular ubiquitin ligase E6AP and the p53 tumor suppressor protein leads to proteolytic p53 degradation and plays a central role for HPV-induced cell transformation. We here uncover that E6AP silencing in HPV-positive cancer cells ultimately leads to efficient induction of cellular senescence, revealing that E6AP acts as a potent anti-senescent factor in these cells. Thus, although the downregulation of either E6 or E6AP expression also acts partially pro-apoptotic, HPV-positive cancer cells surviving E6 repression proliferate further, whereas they become irreversibly growth-arrested upon E6AP repression. We moreover show that the senescence induction following E6AP downregulation is mechanistically highly dependent on induction of the p53/p21 axis, other than the known pro-senescent response of HPV-positive cancer cells following combined downregulation of the viral E6 and E7 oncoproteins. Of further note, repression of E6AP allows senescence induction in the presence of the anti-senescent HPV E7 protein. Yet, despite these mechanistic differences, the pathways underlying the pro-senescent effects of E6AP or E6/E7 repression ultimately converge by being both dependent on the cellular pocket proteins pRb and p130. Taken together, our results uncover a hitherto unrecognized and potent anti-senescent function of the E6AP protein in HPV-positive cancer cells, which is essential for their sustained proliferation. Our results further indicate that interfering with E6AP expression or function could result in therapeutically desired effects in HPV-positive cancer cells by efficiently inducing an irreversible growth arrest. Since the critical role of the E6/E6AP/p53 complex for viral transformation is conserved between different oncogenic HPV types, this approach could provide a therapeutic strategy, which is not HPV type-specific.

## Author summary

Tumor-associated types of human papillomaviruses (HPVs) cause approximately 5% of all cancers in humans, including cervical cancer and a significant portion of head and neck cancers. The elucidation of the pathways that are critical for the growth of HPV-positive cancer cells is an important basis for the development of novel treatment strategies. A key step for the malignant cell transformation by HPVs is the interaction of the viral E6 protein with the cellular E6AP protein. We here show that the downregulation of E6AP results in a strong and irreversible growth arrest of HPV-positive cancer cells by inducing a growth-inhibitory process called cellular senescence. We furthermore provide insights into the molecular mechanisms by which E6AP downregulation leads to senescence. Our findings uncover a hitherto unrecognized function of E6AP in HPV-positive cancer cells, revealing that it acts as a potent anti-senescent factor. Moreover, we show that this activity of E6AP is essential for maintaining the growth of HPV-positive cancer cells. These findings indicate that E6AP is a critical determinant for sustaining the growth of HPV-positive cancer cells and raise the possibility that blocking E6AP in these cells could provide a novel treatment strategy.

## Introduction

Oncogenic types of human papillomaviruses (HPVs), such as HPV16 and HPV18, are major carcinogens, being closely linked to almost 5% of the total cancer incidence in humans [1]. These include prevalent cancers, such as cervical cancer, which alone accounts for over 300,000 new cancer cases and over 600,000 cancer deaths each year worldwide [2]. In view of this substantial HPV-linked cancer burden, it is of high interest to gain detailed insights into the molecular pathways that determine the tumorigenic phenotype of HPV-positive cancer cells. Their understanding may also pave the way for novel treatment strategies.

A large body of experimental evidence has demonstrated that the expression of the viral *E6/E7* oncogenes plays a key role for the induction of HPV-linked cell transformation as well as for the maintenance of the malignant growth of cervical cancer cells [3–6]. A main transforming activity of the anti-apoptotic E6 oncoprotein [7] is the formation of a trimeric complex with the cellular ubiquitin ligase E6AP (E6-associated protein, encoded by the *UBE3A* gene) and the tumor suppressor protein p53, leading to the proteolytic degradation of p53 [8]. Notably, the oncogenic activity of HPV16 E6 was found to be E6AP-dependent in transgenic mice [9]. Critical targets for the transforming activity of the anti-senescent E7 oncoprotein [10] include cellular pocket proteins such as the retinoblastoma tumor suppressor protein pRb and p130, which are destabilized by E7 [11–13]. In addition, E7 can lead to cell transformation through pRb-independent processes, e.g., by inducing the degradation of the protein tyrosine phosphatase PTPN14, which promotes YAP1 signaling [14,15].

The finding that the proliferation of HPV-positive cervical cancer cells requires the sustained expression of the viral *E6/E7* oncogenes suggests that they represent attractive targets for therapeutic interference [16]. Indeed, the inhibition of E6/E7 expression in HPV-positive cervical cancer cells leads to a rapid and efficient induction of cellular senescence [17–19], which is classically defined as an irreversible growth arrest [20]. Further, blocking E6 expression or function alone [21–30] or silencing E6AP expression [24,31–33] were found to exert pro-apoptotic effects in HPV-positive cancer cells, although the extent of apoptosis varies between different studies and experimental conditions.

In the present work, we focused on analyzing the long-term effects of E6AP repression in HPV-positive cancer cells. Interestingly, we found that E6AP repression ultimately leads to highly efficient induction of cellular senescence, revealing that E6AP acts as a potent anti-senescent factor in these cells. As a consequence, cells surviving apoptosis induced by E6AP repression become irreversibly growth arrested, in contrast to cells surviving E6 repression, which continue to proliferate. Moreover, we delineate differences in the mechanisms underlying the anti-proliferative and pro-senescent responses of HPV-positive cancer cells upon E6AP or E6/E7 silencing, particularly with regard to the differential contribution of the p53/p21 axis. Overall, our findings reveal a potent anti-senescent role for E6AP, which is essential for sustaining the proliferation of HPV-positive cancer cells by keeping them in a non-senescent state.

## Results

### E6AP downregulation efficiently induces senescence in HPV-positive cancer cells

**Fig 1:**
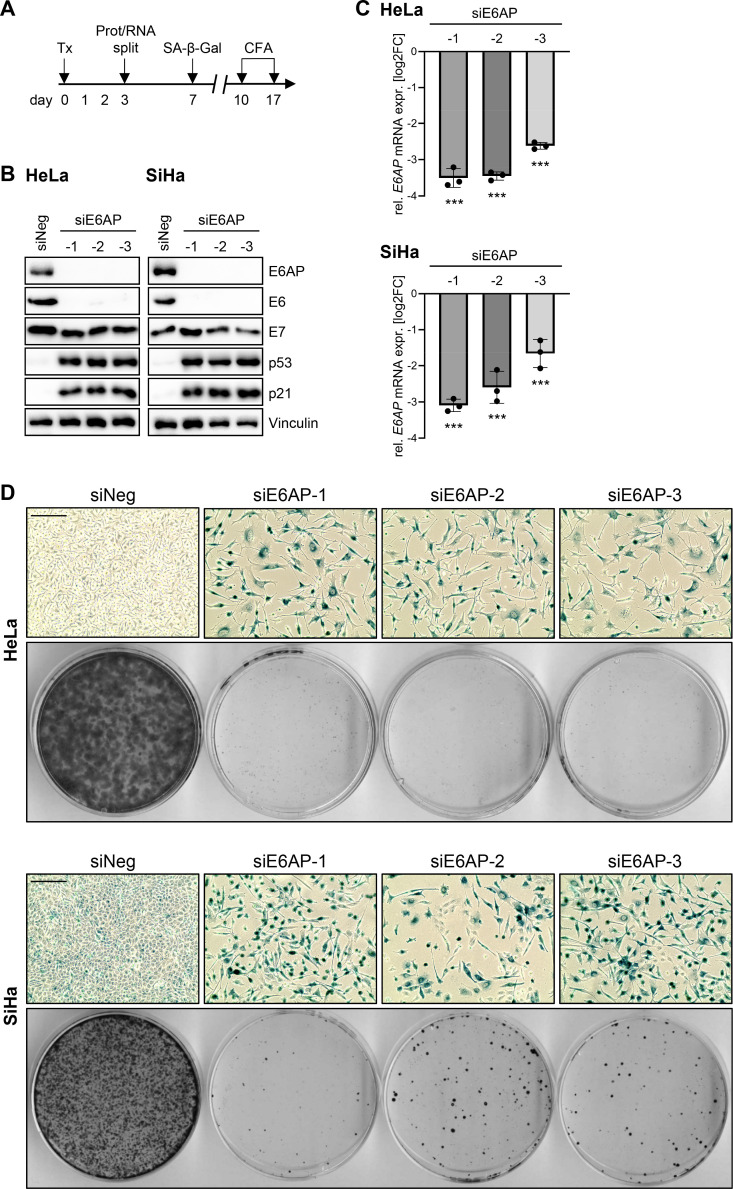
Downregulation of E6AP induces senescence in HPV-positive cancer cells. **(A)** Treatment scheme: HeLa or SiHa cells were reverse transfected (Tx) with three different E6AP-specific siRNAs (siE6AP-1, -2, -3), or control siRNA (siNeg). 72 h post-transfection, cells were either harvested for protein (Prot) or RNA analyses, or split and further cultivated for senescence assays (SA-β-Gal staining) or colony formation assays (CFAs) after the indicated time periods. **(B)** Immunoblot analyses of E6AP, E6, E7, p53, p21, and Vinculin protein levels. **(C)** qRT-PCR analyses of *E6AP* mRNA levels. Shown are log2-transformed fold changes (log2FC) of mean expression with standard deviations (*n* = 3). Statistically significant differences between cells transfected with siE6AP-1, -2, or -3, and those transfected with siNeg (log2FC = 0) were assessed using one-way ANOVA with Sidak’s test for multiple comparisons. *** *p* ≤ 0.001. **(D)** Corresponding senescence assays (upper panels; SA-β-Gal staining, blue; scale bar: 200 µm) and CFAs (lower panels).

In order to investigate the cellular fate resulting from E6AP repression, E6AP was downregulated in HPV18-positive HeLa and HPV16-positive SiHa cells by RNAi (RNA interference) and the phenotype of the cells was monitored over time (Fig 1A). We employed three different siRNAs (small interfering RNAs) targeting different regions in the *E6AP* transcript, each leading to an efficient downregulation of E6AP protein levels to virtually undetectable levels (Fig 1B) and a pronounced reduction of *E6AP* transcript levels (Fig 1C), in both HeLa and SiHa cells. In line with previous reports, the interference with E6AP expression was associated with a substantial decrease in E6 protein levels [33–35] as well as with an increase in p53 [30,31,33,36] and p21 levels [24,36] (Fig 1B).

Interestingly, we observed that both HeLa and SiHa cells reacted to the downregulation of E6AP with an efficient induction of cellular senescence, as shown by the typical morphological changes (cellular enlargement and flattening, cytoplasmic extensions) and positive staining for the well-established senescence marker SA-β-Gal (Senescence-Associated-β-Galactosidase) [37] (Fig 1D, upper panels for each cell line). The irreversible growth arrest resulting from cellular senescence typically leads to the inhibition of colony formation [38]. In line, a strong reduction in colony outgrowth was detectable in HeLa and SiHa cells following E6AP silencing (Fig 1D, lower panels for each cell line). In strong contrast, E6AP silencing in HPV-negative, p53 wildtype RKO, HCT116, and U2OS cancer cells did not result in an appreciable increase in senescence (S1A Fig, upper panels for each cell line), and led to an only weak increase of p53 and p21 levels (S1B Fig) and a weak decrease in colony formation capacity, if at all (S1A Fig, lower panels for each cell line).

To further corroborate that E6AP repression results in senescence of HPV-positive cancer cells, we employed the CRISPR/Cas9 gene editing technology as an alternative experimental approach. Targeting the *UBE3A/E6AP* gene in HeLa cells by using two different guide RNAs resulted in downregulation of E6AP levels, concomitant downregulation of E6 levels, upregulation of p53 and p21 levels, and induction of senescence (S2A-C Fig).

Collectively, our findings uncover that E6AP acts as a potent anti-senescent factor in HPV-positive cancer cells.

### The fates of HPV-positive cancer cells surviving E6AP or E6 repression differ drastically

The pronounced anti-senescent activity of E6AP indicates that – besides targeting the HPV oncogenes – blocking E6AP expression could provide a novel strategy to efficiently inhibit the growth of HPV-positive cancer cells. Therefore, and to gain further insights into the mechanisms underlying the anti-proliferative effects of E6AP repression, we next compared the consequences of E6AP, E6, or E6/E7 silencing for the phenotype of HPV-positive cancer cells.

**Fig 2:**
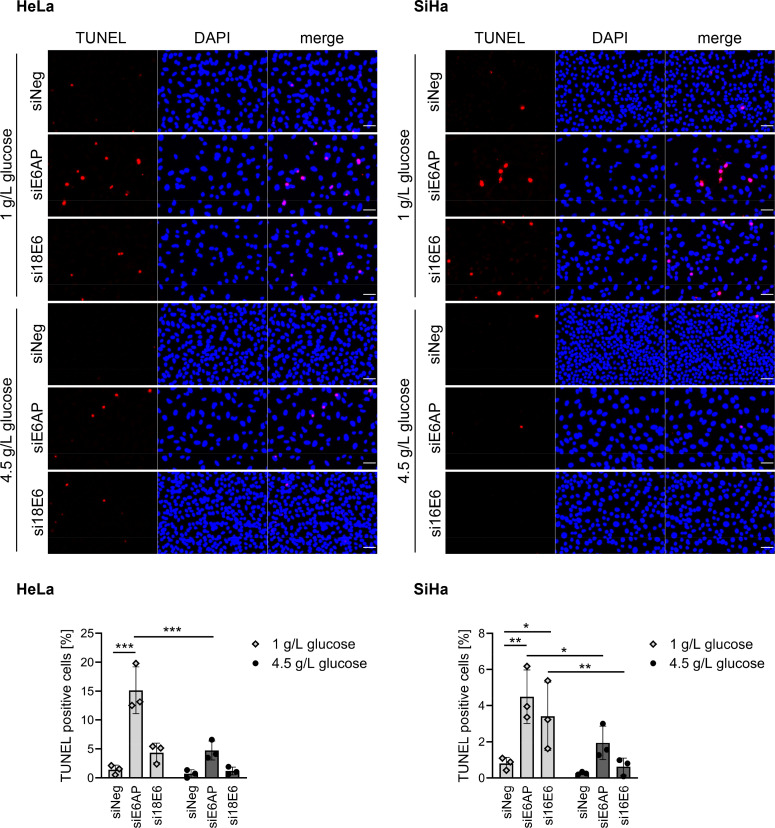
Downregulation of E6AP or E6 exerts pro-apoptotic effects in HPV-positive cancer cells. HeLa or SiHa cells were transfected with 20 nM of siE6AP, si18E6 or si16E6, or control siRNA (siNeg), and cultivated under either 1 g/L or 4.5 g/L glucose in the cell culture medium for 96 h. Upper panels: TUNEL analyses of HeLa or SiHa cells (scale bar: 50 µm). Lower panels: Quantification of TUNEL positive (apoptotic) cells relative to the number of DAPI-stained cells. Results are presented as mean percentages with standard deviations (*n* = 15 fields of view, each with ≥ 50 cells from three independent experiments). Statistically significant differences for cells transfected with siNeg compared to siE6AP or siE6 and for cells cultivated under 1 g/L or 4.5 g/L glucose were assessed using two-way ANOVA with Tukey’s test for multiple comparisons. * *p* ≤ 0.05, ** *p* ≤ 0.01, *** *p* ≤ 0.001.

Previous studies have shown that interference with E6AP or E6 expression exerts pro-apoptotic effects in cervical cancer cells, with the extent of apoptosis varying between different experimental conditions (see Introduction). TUNEL (Terminal deoxynucleotidyl transferase-mediated UTP end labeling) assays revealed an increase in the number of apoptotic cells upon either E6AP or E6 silencing, in both HeLa and SiHa cells (Fig 2). Notably, the extent of apoptosis was linked to cell culture conditions such as glucose supply in the medium, with lower glucose concentrations increasing the apoptotic response (Fig 2).

**Fig 3:**
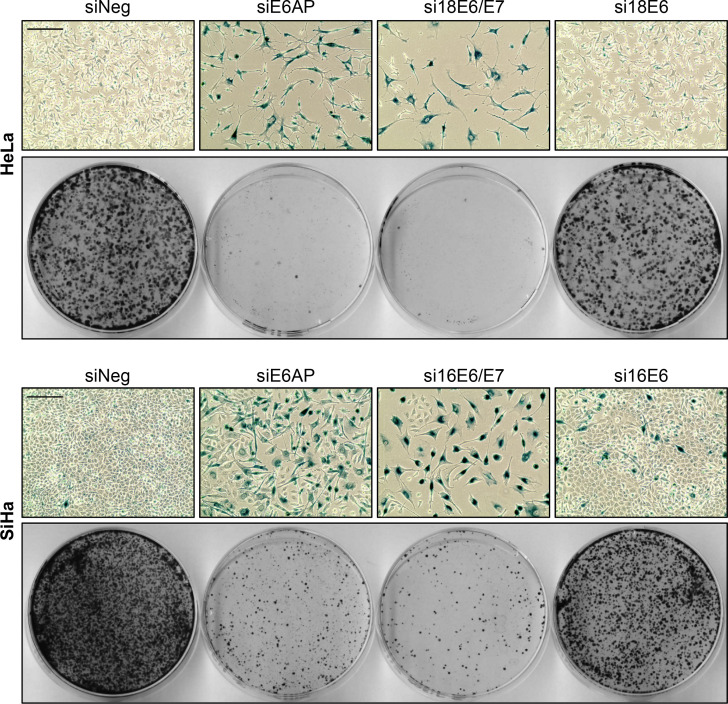
Differential effects of downregulating E6AP, E6/E7, or E6 on the senescence response of HPV-positive cancer cells. HeLa or SiHa cells were transfected with siE6AP, si18E6/E7 or si16E6/E7, si18E6 or si16E6, or control siRNA (siNeg) and further examined following the treatment scheme depicted in Fig 1A. Shown are corresponding senescence assays (upper panels; SA-β-Gal staining, blue; scale bar: 200 µm) and CFAs (lower panels).

Further, we compared the cellular fates upon downregulation of E6AP, E6, or E6/E7 over time, following the experimental scheme depicted in Fig 1A. In contrast to the strong pro-senescent effects of E6AP or E6/E7 repression, downregulation of E6 alone only rarely led to signs of senescence in HeLa and SiHa cells (Fig 3, upper panels for each cell line). This differential response was mirrored by a much more pronounced inhibition of colony formation in both HeLa and SiHa cells upon E6AP or E6/E7 silencing, compared to E6 silencing alone (Fig 3, lower panels for each cell line).

Taken together, these results reveal that the phenotypic responses of HPV-positive cancer cells upon E6 or E6AP repression strongly differ, since HPV-positive cancer cells surviving E6AP repression react with an irreversible growth arrest, whereas cells surviving E6 repression continue to grow.

### E6AP, E6, or E6/E7 silencing exerts differential effects on proliferation and cell cycle regulation

**Fig 4:**
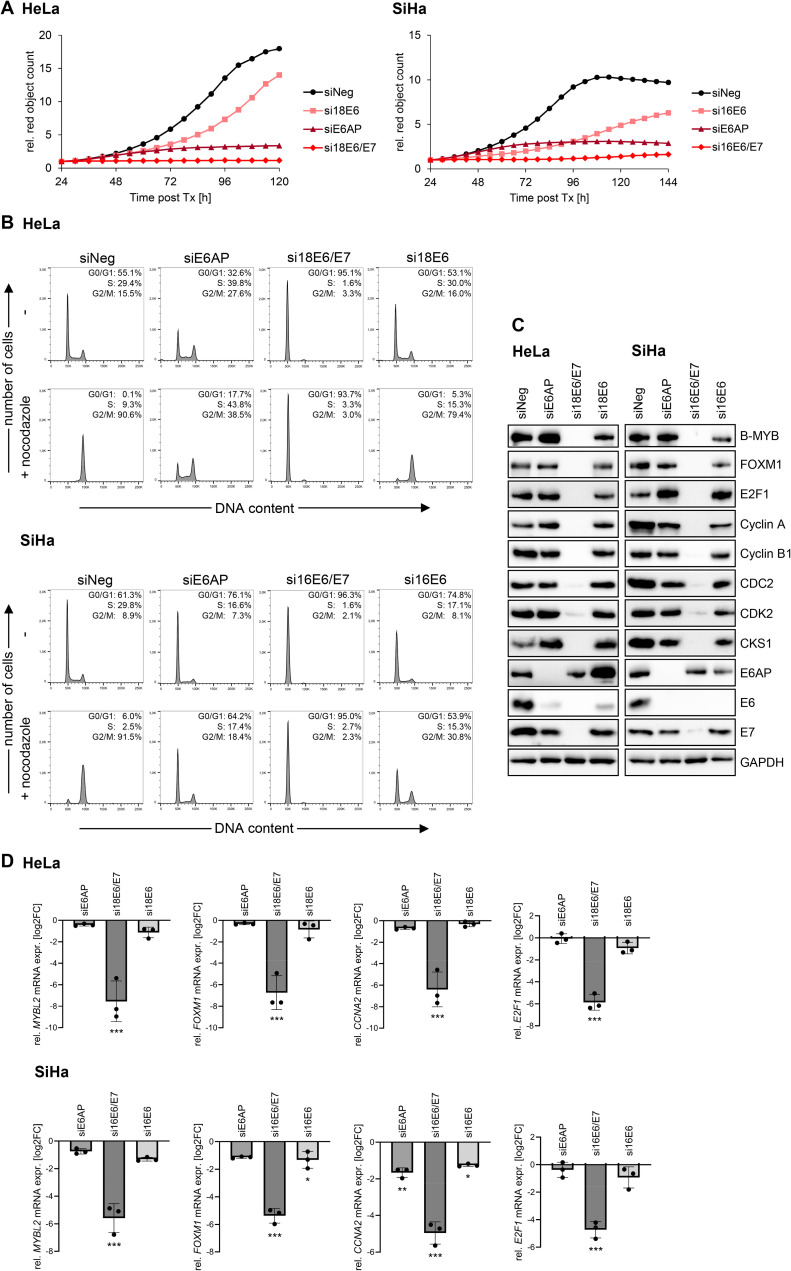
Differential effects of downregulating E6AP, E6/E7, or E6 on the cell cycle regulation of HPV-positive cancer cells. **(A)** HeLa-mKate2 or SiHa-mKate2 cells were transfected with siE6AP, si18E6/E7 or si16E6/E7, si18E6 or si16E6, or control siRNA (siNeg) and imaged by the Incucyte S3 live-cell imaging system for up to 144 h post-transfection. Cell counts (red objects) were quantified using the Incucyte software package and are shown relative to the counts at 24 h post-transfection (set to 1). Rel, relative; Tx, transfection. **(B)** HeLa or SiHa cells were transfected with siE6AP, si18E6/E7 or si16E6/E7, si18E6 or si16E6, or control siRNA (siNeg), cultivated for 72 h, and treated with nocodazole or solvent control (-) for the last 16 h (HeLa) or 24 h (SiHa) of cultivation. Cell cycle profiles and quantifications of the percentages of cell populations in the individual cell cycle phases are shown. **(C)** HeLa or SiHa cells were transfected with siE6AP, si18E6/E7 or si16E6/E7, si18E6 or si16E6, or control siRNA (siNeg), cultivated for 72 h, and examined by immunoblot for B-MYB, FOXM1, E2F1, Cyclin A, Cyclin B1, CDC2, CDK2, CKS1, E6AP, E6, E7, and GAPDH protein levels. **(D)** Corresponding qRT-PCR analyses of *MYBL2* (coding for B-MYB), *FOXM1*, *CCNA2* (coding for Cyclin A2), and *E2F1* mRNA levels. Shown are log2-transformed fold changes (log2FC) of mean expression with standard deviations (*n* = 3). Statistically significant differences between cells transfected with siE6AP, siE6/E7, or siE6, and those transfected with siNeg (log2FC = 0) were assessed using one-way ANOVA with Sidak’s test for multiple comparisons. * *p* ≤ 0.05, ** *p* ≤ 0.01, *** *p* ≤ 0.001.

To gain further insights into the divergent phenotypic effects in response to E6AP, E6, or E6/E7 repression, we performed comparative proliferation assays and cell cycle analyses. Growth curves obtained by live-cell imaging revealed that the proliferation of both HeLa and SiHa cells was rapidly arrested by silencing E6/E7 expression and with some delay, but also with high efficiency, by silencing E6AP expression (Fig 4A). In contrast, upon E6 silencing, the cells resumed their proliferation after an only temporary slowdown.

Interestingly, despite both E6AP and E6/E7 repression resulted in growth arrest and senescence induction in HeLa and SiHa cells, we observed strikingly different effects on their cell cycle distribution. In specific, E6/E7 silencing resulted in highly efficient G1 arrest in both cell lines, as visualized 72 h after transfection (Fig 4B). In contrast, the effects of E6AP or E6 silencing on the cell cycle profiles were much less pronounced. In HeLa cells, E6AP silencing led to a limited increase in S and G2/M phase populations, whereas E6 silencing alone did not appreciably affect the cell cycle distribution. In SiHa cells, both E6AP and E6 silencing led to a weak increase of cells in G1 populations, when compared to control siRNA-treated cells.

In addition, we concomitantly treated the cells with the microtubule polymerization inhibitor nocodazole, which targets proliferating cells, leading to G2/M arrest [39]. Unlike control siRNA-transfected cells, which retained their cycling capacities and entered a nocodazole-induced G2/M arrest, siE6/E7-transfected cells were confirmed to be efficiently G1-arrested, as their cell cycle profile remained unchanged upon nocodazole treatment (Fig 4B). Similarly, upon E6AP silencing, nocodazole treatment led to an only weak increase in G2/M phase populations in both HeLa and SiHa cells, indicating a marked reduction in their cycling capacities. After silencing E6 alone, nocodazole treatment strongly affected the cell cycle profile of HeLa cells, indicating that these cells proliferate. In SiHa cells, nocodazole treatment revealed decreased cycling activities 72 h after transfection, which corresponded to the growth curve at that time point (Fig 4A). Thus, although both E6/E7 and E6AP silencing lead to growth arrest and efficient induction of cellular senescence, the corresponding cell cycle profiles differ strongly.

Of further note, we found that only E6/E7 repression, but not E6 or E6AP repression, led to a strong downregulation of a spectrum of factors which can promote cell cycle progression [40,41], such as B-MYB, FOXM1, E2F1, Cyclin A, Cyclin B1, CDC2 (CDK1), CDK2, and CKS1 (Fig 4C). Concomitant RNA analyses of a selection of genes encoding these factors indicate that the downregulation occurred at the transcript level (Fig 4D).

In summary, and consistent with the results of the senescence assays (Fig 3) and proliferation assays (Fig 4A), these findings show that both HeLa and SiHa cells are strongly impaired in their growth following silencing of E6AP or E6/E7 expression, whereas they resume proliferation upon E6 silencing alone. Of further interest, E6/E7 repression is linked to a pronounced G1 arrest and efficient downregulation of cell cycle-promoting genes, which is not observed upon E6AP repression, albeit the cells are effectively growth arrested under both conditions.

### The anti-proliferative and pro-senescent effects of E6AP repression are highly dependent on the p53/p21 axis

**Fig 5:**
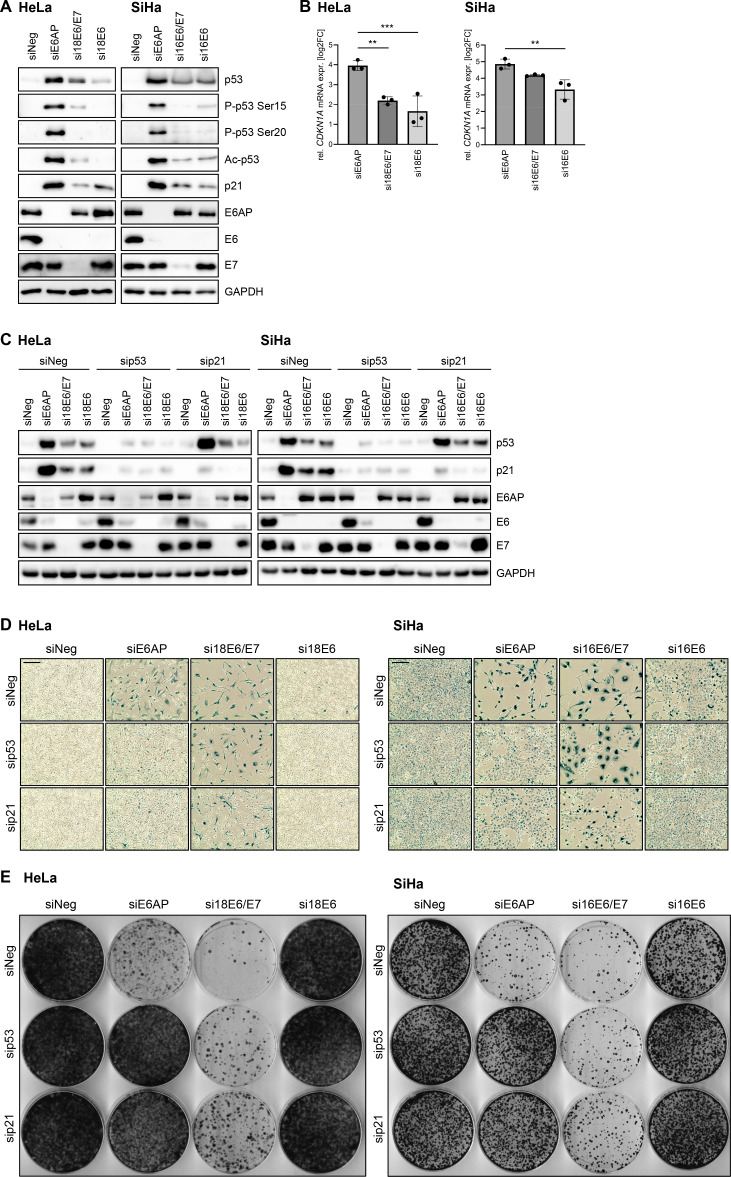
p53 and p21 are critical for senescence induction upon E6AP downregulation. **(A)** HeLa or SiHa cells were transfected with siE6AP, si18E6/E7 or si16E6/E7, si18E6 or si16E6, or control siRNA (siNeg), cultivated for 72 h, and examined by immunoblot for p53, P-p53 Ser15, P-p53 Ser20, Ac-p53 Lys382, p21, E6AP, E6, E7, and GAPDH protein levels. **(B)** qRT-PCR analyses of *CDKN1A* (coding for p21) mRNA levels. Shown are log2-transformed fold changes (log2FC) of mean expression with standard deviations (*n* = 3). Statistically significant differences between cells transfected with siE6AP, siE6/E7, or siE6, and those transfected with siNeg (log2FC = 0) were assessed using one-way ANOVA with Sidak’s test for multiple comparisons. ** *p* ≤ 0.01, *** *p* ≤ 0.001. **(C-E)** HeLa or SiHa cells were transfected with siE6AP, si18E6/E7 or si16E6/E7, si18E6 or si16E6, or control siRNA (siNeg), alone or in combination with sip53 or sip21, as indicated. Cells were examined following the treatment scheme depicted in Fig 1A by **(C)** immunoblot for p53, p21, E6AP, E6, E7, and GAPDH protein levels **(D)** senescence assays (SA-β-Gal staining, blue; scale bar: 200 µm), and **(E)** corresponding CFAs.

To further explore the mechanisms underlying the growth inhibition and senescence induction following E6AP repression, we analyzed the role of p53 and p21, which are key factors for the senescence response under many, but not all biological conditions [20]. Blocking expression of E6AP, E6/E7, or E6 led to a pronounced increase in p53 and p21 protein levels (Fig 5A). Interestingly, however, we consistently observed a substantially higher induction of p53 and p21 following E6AP silencing, compared to E6/E7 or E6 silencing (Fig 5A).

The expression of p53 and p21 can be directly coupled, since the *CDKN1A* gene (coding for p21) is a transcriptional target of p53 [42], however, p53-independent mechanisms of p21 induction have also been found [43]. Moreover, the transactivation of p53 target genes, including *CDKN1A*, has been linked to specific p53 modifications, such as phosphorylation at Ser15 and Ser20 or acetylation of Lys382 [44–46], which do not necessarily correlate with total p53 amounts [47]. Therefore, we examined whether these post-translationally modified p53 forms are also differentially affected by E6AP, E6/E7, or E6 silencing. Indeed, we detected substantially higher levels of P-p53 Ser15, P-p53 Ser20, and Ac-p53 Lys382 upon E6AP silencing, compared to E6/E7 or E6 silencing (Fig 5A). In line with the higher concentrations of transactivating p53 forms, E6AP silencing also resulted in a higher increase in *CDKN1A* transcript levels than E6/E7 or E6 silencing (Fig 5B).

Next, we analyzed the functional contribution of the p53/p21 axis to the senescence induction upon E6AP or E6/E7 silencing. Interestingly, concomitant silencing of E6AP expression on the one hand, and p53 or p21 expression on the other hand (Fig 5C), efficiently counteracted the senescence induction (Fig 5D) as well as the repression of the colony formation capacity (Fig 5E), which was observed upon silencing E6AP expression alone, in both HeLa and SiHa cells. In strong contrast, the efficiency of senescence induction upon E6/E7 repression was only marginally decreased when p53 expression was concomitantly silenced (Fig 5C-E). The combined downregulation of E6/E7 and p21 (Fig 5C) weakly allowed senescence evasion, as indicated by the emergence of cells lacking signs of senescence next to senescent cells (Fig 5D), as well as by an increase in colony formation capacities, compared to E6/E7 silencing alone (Fig 5E).

**Fig 6:**
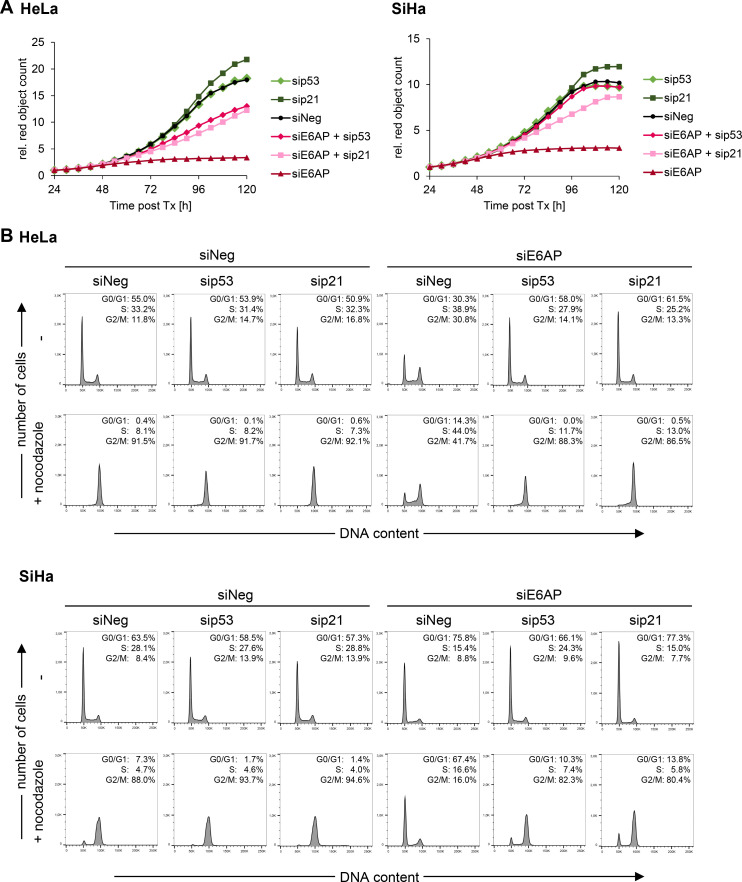
p53 and p21 are critical for cell cycle changes upon E6AP downregulation. **(A)** HeLa-mKate2 or SiHa-mKate2 cells were transfected with siE6AP or control siRNA (siNeg), either alone or in combination with sip53 or sip21, as indicated, and imaged by the Incucyte S3 live-cell imaging system for up to 120 h post-transfection. Cell counts (red objects) were quantified using the Incucyte software package and are shown relative to the counts at 24 h post-transfection (set to 1). Rel, relative; Tx, transfection. **(B**) Cell cycle analyses of HeLa or SiHa cells, transfected with siE6AP or control siRNA (siNeg), either alone or in combination with sip53 or sip21, as indicated. Cells were cultivated for 72 h and treated with nocodazole or solvent control (-) for the last 16 h (HeLa) or 24 h (SiHa) of cultivation. Cell cycle profiles and quantifications of the percentages of cell populations in the individual cell cycle phases are shown.

Subsequently, we investigated whether the inhibitory effects on the proliferation rate (Fig 4A) and the changes in cell cycle distribution (Fig 4B) of cervical cancer cells in response to E6AP repression are also p53- and p21-dependent. Silencing of p53 or p21 expression alone had no substantial influence on the growth rate of either HeLa or SiHa cells (Fig 6A). Notably, however, we observed that the growth-inhibitory effect of E6AP repression was efficiently counteracted by concomitant silencing of either p53 or p21 expression, in both HeLa and SiHa cells (Fig 6A). Furthermore, their cell cycle profiles resembled that of control siRNA-transfected cells (Fig 6B) and treatment with nocodazole led to a strong increase in G2/M populations (Fig 6B), confirming that the cells were proliferating.

**Fig 7:**
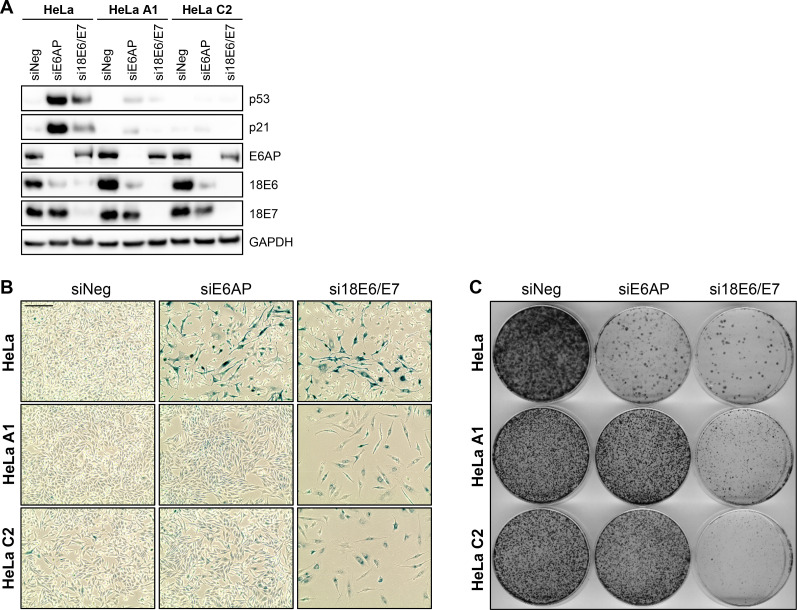
Downregulation of E6AP does not induce senescence in “p53 null” cells. HeLa or HeLa “p53 null” cells (HeLa A1, HeLa C2) were transfected with siE6AP, si18E6/E7, or control siRNA (siNeg). Cells were examined following the treatment scheme depicted in Fig 1A by **(A)** immunoblot for p53, p21, E6AP, 18E6, 18E7, and GAPDH protein levels **(B)** senescence assays (SA-β-Gal staining, blue; scale bar: 200 µm), and **(C)** corresponding CFAs.

To further corroborate the differential role of p53 for the senescence induction upon E6AP or E6/E7 repression, we comparatively analyzed parental HeLa cells and two HeLa cell clones in which p53 expression is efficiently silenced by stable RNAi [31] (Fig 7A). As shown by the morphological changes and positive senescence assays in these cells (Fig 7B), and further supported by a strong reduction of their colony formation capacities (Fig 7C), E6/E7 silencing efficiently induced senescence irrespective of their p53 status. In contrast, whereas the senescence induction upon E6AP silencing was readily detectable in parental HeLa cells, it was strongly impaired in HeLa cells in which p53 expression is stably repressed (Fig 7B and 7C).

Collectively, these data reveal strong differences in the role of the p53/p21 axis for the anti-proliferative and pro-senescent effects of E6AP or E6/E7 repression in HPV-positive cancer cells, since these phenotypic responses are highly dependent on p53 and p21 induction upon E6AP repression, but not upon E6/E7 repression.

### Senescence induction following E6AP repression depends on pRb and p130

**Fig 8:**
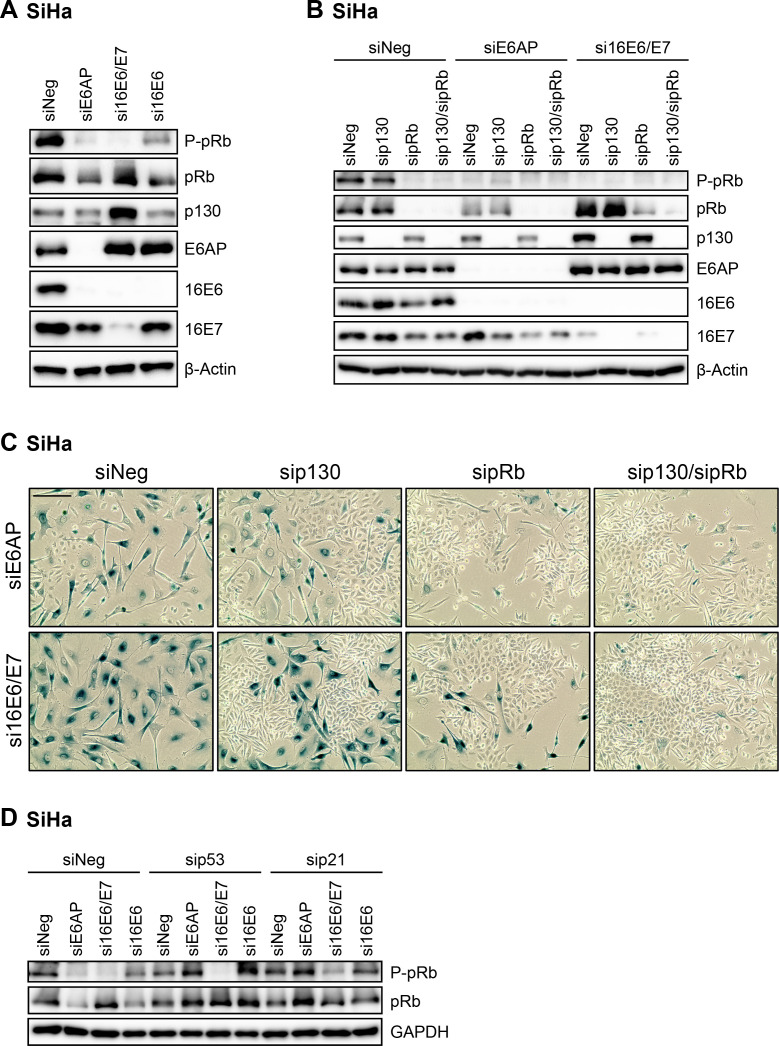
Senescence induction upon E6AP or E6/E7 downregulation depends on p130 and pRb. **(A)** SiHa cells were transfected with siE6AP, si16E6/E7, si16E6, or control siRNA (siNeg). After 72 h, protein levels of P-pRb Ser807/811, total pRb, p130, E6AP, 16E6, 16E7, and β-Actin were measured by immunoblot analyses. **(B,C)** SiHa cells were transfected with siE6AP, si16E6/E7, or control siRNA (siNeg), either alone or in combination with sip130, sipRb, or a combination of both sip130/sipRb, as indicated. Cells were examined following the treatment scheme depicted in Fig 1A by **(B)** immunoblot for P-pRb Ser807/811, total pRb, p130, E6AP, 16E6, 16E7, and β-Actin protein levels and by **(C)** senescence assays (SA-β-Gal staining, blue; scale bar: 200 µm). **(D)** SiHa cells were transfected with siE6AP, si16E6/E7, si16E6, or control siRNA (siNeg), either alone or in combination with sip53 or sip21. Protein levels of P-pRb Ser807/811, total pRb, and GAPDH were assessed 72 h post-transfection by immunoblot analyses.

An important difference between silencing E6/E7 or E6 expression is the maintenance of the E7 protein in the latter case (Figs 1B, 4C, 5A, 5C, 7A, and 8B), which can interfere with the expression and activity of growth-inhibitory pocket proteins, such as pRb and p130 [11–13]. Accordingly, E6/E7 silencing led to increased pRb and p130 amounts and a highly efficient downregulation of phosphorylated pRb levels in SiHa cells, as analyzed 72 h after transfection (Fig 8A). In line with a functional reconstitution of the pocket proteins pRb and p130, E6/E7 silencing was linked to a strong reduction of B-MYB, FOXM1, E2F1, Cyclin A, Cyclin B1, CDC2, CDK2, and CKS1 levels (Fig 4C) [40,41], indicative for an efficient G1 arrest. In contrast, E6 silencing alone did not elevate pRb or p130 levels (Fig 8A), the downregulation of phosphorylated pRb levels was limited (Fig 8A), and B-MYB, FOXM1, E2F1, Cyclin A, Cyclin B1, CDC2, CDK2, and CKS1 levels remained largely unaltered (Fig 4C). To exclude that E6 silencing might more efficiently downregulate phosphorylated pRb levels under alternative experimental conditions, we also examined the cells following transfection of higher siE6 concentrations or upon repeated siE6 transfections for an extended time period of 96 h (S3A Fig). However, also under these conditions, only a limited reduction of phosphorylated pRb levels was detectable (S3B Fig) and the senescence response (S3C Fig, upper panels) as well as the colony formation capacity (S3C Fig, lower panels) was not appreciably altered.

Upon E6AP silencing, total pRb and p130 levels were not increased or even slightly decreased, and E7 expression was maintained (Fig 8A). Notably, however, phosphorylated pRb levels were efficiently reduced upon E6AP silencing, raising the possibility that pocket proteins may be active and participate in the induction of senescence, albeit E7 is present. Therefore, we analyzed the functional contribution of pRb and p130 to the senescence induction following either E6AP or E6/E7 silencing by concomitantly silencing pRb or p130, alone or in combination (Fig 8B). As shown by the emergence of cells negative for SA-β-Gal staining and lacking the typical morphological signs of senescence, downregulation of pRb and/or p130 counteracted siE6AP- as well as siE6/E7-induced senescence in SiHa (Fig 8C) and HeLa cells (S4 Fig), indicating that both pRb and p130 are functionally relevant for the senescence induction upon either E6AP or E6/E7 repression in these cells.

The p53/p21 axis can be functionally linked to pRb, since an increase in p21 leads to CDK inhibition and subsequent hypophosphorylation and activation of pRb [48]. Interestingly, we found that silencing of p53 or p21 together with E6AP silencing was linked to pRb phosphorylation, which, in contrast, was at most only marginally observable when combining silencing of p53 or p21 together with E6/E7 silencing (Fig 8D). These latter findings are in further support of the notion that senescence induction upon E6AP silencing is highly dependent on the p53/p21 axis, as its activation leads to hypophosphorylation of pRb despite the presence of E7.

Overall, these results demonstrate that – despite pronounced differences in the functional significance of the p53/p21 axis – the pathways underlying the pro-senescent responses of HPV-positive cancer cells upon E6AP or E6/E7 silencing eventually converge and are both pRb- and p130-dependent.

## Discussion

In this study, we uncover a hitherto unrecognized activity for E6AP, revealing that it acts as a potent anti-senescent factor in HPV-positive cancer cells. We further show that this activity of E6AP is essential for maintaining the proliferation of HPV-positive cancer cells. Targeting E6AP may therefore provide an attractive strategy to effectively block the growth of HPV-positive cancer cells.

The senescence induction upon E6AP repression is highly efficient, being comparable to the known pro-senescent response of HPV-positive cancer cells upon E6/E7 repression. Yet, we discovered differences between the mechanisms underlying both anti-proliferative responses. Cell cycle analyses revealed that E6/E7 repression led to a highly efficient accumulation of the cells in the G1 phase, which was not observed upon E6AP repression. More recent data has shown that E7, which is maintained upon E6AP repression, not only interferes with the function of pRb, but also impairs the activity of the multiprotein DREAM (dimerization partner, RB-like, E2F, and multi-vulval class B complex) complex by interfering with its key component p130 [49]. The DREAM complex can support induction of G1 arrest and senescence by transcriptionally repressing a broad spectrum of cell cycle-promoting genes, which partly overlap with pRb-repressed genes [40,41,49,50]. Indeed, we found that E6/E7 silencing was linked to a strong downregulation of pRb and/or DREAM targets, such as B-MYB, FOXM1, E2F1, Cyclin A, Cyclin B1, CDC2, CDK2, and CKS1 [41]. In strong contrast to E6/E7 repression, the effects of E6AP repression on cell cycle profiles and expression of pRb/DREAM targets were much less pronounced, although proliferation was also efficiently blocked. This suggests that the growth inhibition upon E6AP repression largely occurs through slowing down cell cycle progression in several cell cycle phases, thereby not leading to a pronounced accumulation of cells in a distinct cell cycle phase. This scenario could also explain why the downregulation of pRb/DREAM targets, which are expressed in a cell cycle phase-dependent manner [40,41,49], is only weakly detectable upon silencing E6AP expression.

Interestingly, albeit E6AP and E6/E7 repression both increase p53 and p21 levels, we found that only the anti-proliferative and pro-senescent effects resulting from E6AP repression were highly dependent on induction of the p53/p21 axis, since they could be efficiently blocked by concomitant silencing of either p53 or p21. In strong contrast, the anti-proliferative and pro-senescent responses of HPV-positive cancer cells following E6/E7 repression were only weakly affected, if at all, by concomitant silencing of p53 or p21. These findings indicate a differential functional role of the p53/p21 axis in both pro-senescent processes.

It is also interesting that E6AP silencing – other than E6 silencing – leads to efficient senescence, albeit E7 levels are maintained under both conditions. One possible explanation may be our finding that the increase in total and transactivating phosphorylated/acetylated p53 forms as well as the increase in p21 levels was substantially higher upon E6AP repression than upon E6 repression. This may be possibly linked to the observation that E6AP silencing leads to efficient downregulation not only of E6AP but also of E6, consistent with the notion that E6 stability is dependent on the presence of E6AP [34]. In turn, this increased p21 induction could result in more efficient CDK inhibition. Indeed, E6AP silencing is associated with a sustained decrease of phosphorylated pRb, other than E6 silencing. Thus, by more efficiently activating the p53/p21 axis, E6AP silencing would be expected to lead to increased amounts of hypophosphorylated pRb and p130 proteins, which might not be efficiently sequestered by E7 anymore.

**Fig 9:**
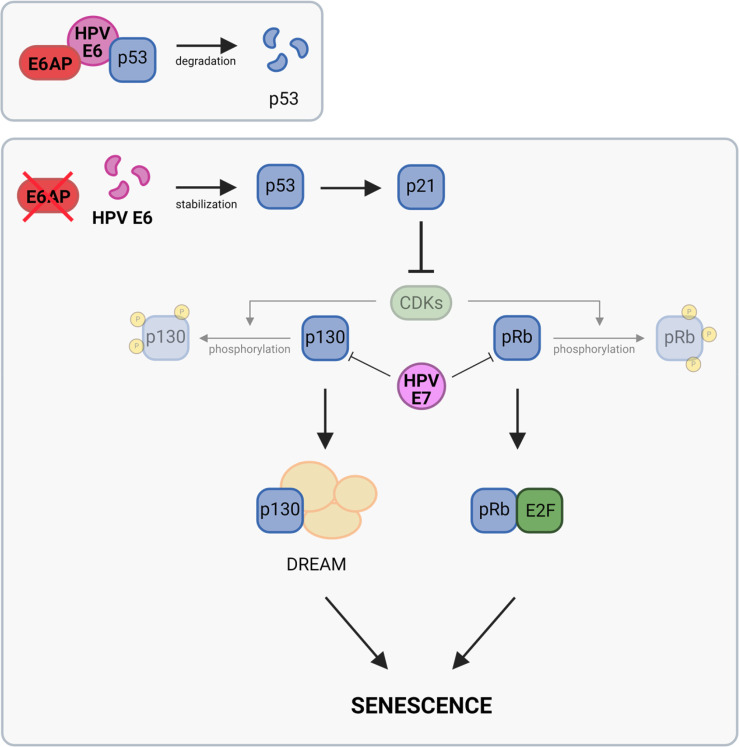
Tentative model for the pro-senescent effect of E6AP downregulation. In HPV-positive cancer cells, E6AP forms a trimeric complex with E6 and p53, leading to the proteolytic degradation of p53 (upper panel). Formation of this complex is abolished upon downregulation of E6AP, and E6 is destabilized (lower panel). This leads to a strong upregulation of p53 and p21 levels, and subsequent inhibition of the phosphorylation of p130 and pRb through p21-mediated suppression of CDKs. Both p130 and pRb are important for senescence induction upon E6AP downregulation, e.g., by being a key component of the multiprotein DREAM complex (p130) or by inhibiting E2F (pRb). Notably, senescence induction upon E6AP downregulation can take place in the presence of E7 (please refer to the discussion in the text). Created with BioRender.com.

Collectively, our results could be integrated into the following tentative model for the pro-senescent activity of E6AP repression (Fig 9). In HPV-positive cancer cells, E6AP binds to E6, which subsequently leads to the formation of a trimeric E6/E6AP/p53 complex (upper panel). This complex results in the proteolytic degradation of p53 via the ubiquitin ligase activity of E6AP. However, upon E6AP repression (lower panel), the concomitant downregulation of both E6AP and E6 results in strongly increased p53 (including post-translationally modified transactivating p53 forms) and p21 levels. This, in turn, leads to efficient activation (hypophosphorylation) of p130 and pRb by the interference of p21 with CDK activities [48,51]. In contrast, following E6/E7 silencing, the E7 depletion leads to the release of p130 and pRb from their detrimental interactions with E7, allowing direct stimulation of pRb- and DREAM-linked pro-senescent pathways. This effect may therefore not be substantially counteracted by blocking the activation of the p53/p21 axis, which acts further upstream in this regulatory circuit. The pathways underlying the pro-senescent effects of E6AP or E6/E7 ultimately converge at the level of the pocket proteins p130 and pRb, which support senescence by repressing cell cycle-promoting genes, which are under negative transcriptional control of pRb and/or DREAM [40,41,49,50]. In line, we found that silencing pRb or p130 expression counteracts the pro-senescent effects of both E6AP and E6/E7 silencing.

Additionally, other E6AP activities – besides its role for p53 degradation – could contribute to its effects on senescence regulation in cervical cancer cells. Indeed, E6AP likely acts pleiotropic, since numerous cellular proteins have been proposed to serve as substrates for E6AP [52] and, furthermore, E6AP was described to function also as a transcriptional co-activator independent of its role in proteolysis [53,54]. Accordingly, E6AP has been reported to affect several tumor-linked cellular pathways, including Pi3K/AKT-, Wnt/β-Catenin-, PML-, and HIPPO-signaling [55–58], which all also have been linked to senescence regulation [56,59–61]. Whereas we did not observe pro-senescent effects following E6AP silencing in HPV-negative, p53 wildtype RKO and HCT116 colon cancer cells, or U2OS osteosarcoma cells, it was reported that E6AP has pro- or anti-senescent effects in certain cell lines [56,62,63]. Further, in view of the broad array of possible direct and indirect E6AP activities, it is also conceivable that E6AP silencing could indirectly interfere with E7 function in HPV-positive cancer cells, without substantially affecting E7 levels, and thus may allow senescence induction in the presence of E7. In line with this possibility, there is evidence that E7 activities can also be regulated at the post-translational level, e.g., via phosphorylation or polyamination [64].

Previous reports have shown that interfering with E6 or E6AP expression exerts pro-apoptotic effects, albeit the extent of apoptosis varied between different studies (see Introduction). This may be linked to variables in experimental conditions, such as differences in siRNA or drug concentrations, transfection efficiencies, cell densities, availability of nutrients and other serum factors, treatment duration, etc. One of these parameters affecting apoptosis could be differences in glucose supply [65,66]. We indeed observed that the apoptotic responses of cervical cancer cells to E6AP or E6 repression are increased in medium containing 1 g/L glucose (corresponding to normal serum concentrations in humans) when compared to 4.5 g/L glucose (which is also often used in cell culture media). Of note, substantial intercellular metabolic heterogeneity can occur in a given cell population even under cell culture conditions, with some cells containing rather low intracellular glucose levels [67], which potentially may also influence their apoptotic response. Furthermore, the glucose supply in the interstitial fluid of tumors is typically heterogeneously distributed [68] and may be substantially lower than in serum [69]. This raises the possibility that the efficacy of the pro-apoptotic response towards treatment strategies aiming at E6AP or E6 inhibition could be heterogeneously distributed in HPV-positive cancers. In this context, it is also noteworthy that, besides apoptosis, therapy-induced senescence is considered to be a promising strategy for cancer treatment [70,71]. Thus, the additional strong pro-senescent effects of E6AP repression, which we detected in our study, could also be beneficial under therapeutic aspects and advantageous over targeting E6.

Collectively, the findings of our study reveal that E6AP exerts a thus far unrecognized and potent anti-senescent function in HPV-positive cervical cancer cells. The processes promoting the pro-senescent responses following E6AP or E6/E7 repression differ in terms of the role of the p53/p21 axis, but ultimately converge by being both pRb- and p130-dependent. Moreover, E6AP silencing allows induction of senescence in the presence of E7. Our results indicate that E6AP is essential for the proliferation of cervical cancer cells and that interfering with E6AP expression or function could result in therapeutically useful effects by inducing both apoptosis and senescence. Moreover, since the functional significance of the E6/E6AP/p53 complex for HPV-induced carcinogenesis is conserved for different types of oncogenic genital HPVs, targeting E6AP could have the advantage of providing a therapeutic strategy, which is not HPV type-restricted.

## Materials and methods

### Cell culture

HPV18-positive HeLa (RRID: CVCL_0030) and HPV16-positive SiHa (RRID: CVCL_0032) cervical cancer cells, HPV-negative RKO (RRID: CVCL_0504) and HCT116 (RRID: CVCL_0291) colon cancer cells, and HPV-negative U2OS (RRID: CVCL_0042) osteosarcoma cells were obtained from the tumor bank of the German Cancer Research Center (DKFZ), Heidelberg. HeLa “p53 null” cervical cancer cells (HeLa A1 and HeLa C2) were described before [31]. HeLa-mKate2 and SiHa-mKate2 cells stably express a nuclear-restricted mKate2 fluorescent protein and were generated as detailed previously [72]. Cell lines were cultivated in Dulbecco’s minimal essential medium (DMEM) (HeLa, SiHa, U2OS, HeLa A1, HeLa C2, HeLa-mKate2, SiHa-mKate2), RPMI 1640 (RKO), or McCoy’s 5A medium (HCT116) (Gibco, Thermo Fisher Scientific), containing 4.5 g/L glucose (if not specified otherwise), 10% fetal bovine serum (PAN-Biotech) and 2 mM L-glutamine, 100 U/mL penicillin, and 100 µg/mL streptomycin (all from Sigma-Aldrich) at 37 °C, 5% CO_2_, and 21% O_2_. Authentication of the cell lines was performed by single-nucleotide polymorphism profiling within the last year (Multiplexion GmbH), and all experiments were performed with cells validated to be negative for mycoplasma.

### RNA interference

Reverse transfections of small interfering RNAs (siRNAs) (Life Technologies, Thermo Fisher Scientific) were performed using Lipofectamine RNAiMAX (Invitrogen, Thermo Fisher Scientific) according to the manufacturer’s protocol. To silence E6AP expression, three unrelated siRNAs were employed, which target sequences in three different exons of the *UBE3A*/*E6AP* gene: siE6AP-1: 5′-GAAUUUGUUCAUUAUCGUA-3′; siE6AP-2: 5′-CCAAUGAUGUAUGAUCUAA-3′; siE6AP-3: 5′-CGGCUAGAGAUGAUCGCUA-3′ (S5A Fig). The siRNAs were applied either individually or as an equimolar pool targeting all known E6AP isoforms (referred to in the text as siE6AP). HPV18/HPV16 E6 or E6/E7 expression was blocked by using equimolar pools of three different siRNAs (referred to in the text as si18E6, si18E6/E7, si16E6, or si16E6/E7, respectively), which have been characterized before [22,73–78] (S5B Fig). Target sequences of siRNAs silencing p53, p21 (*CDKN1A*), p130, or pRb expression, respectively, were: sip53: 5′-GACUCCAGUGGUAAUCUAC-3′ [79]; sip21 (*CDKN1A*): 5′-CAAGGAGUCAGACAUUUUA-3′ [76]; sip130: 5′-GAGCAGAGCUUAAUCGAAU-3′ [80]; sipRb: 5′-GCGUGUAAAUUCUACUGCA-3′. Control siRNA siNeg, 5′-UACGACCGGUCUAUCGUAG-3′, contains at least 4 mismatches to all known human genes [81–83]. Individual siRNAs and siRNA pools were used at a final concentration of 6 nM to 10 nM, if not stated otherwise. For co-transfection experiments, equimolar amounts of the siRNAs were used and the total siRNA concentration was kept constant across all samples by supplementing with control siRNA siNeg.

### CRISPR/Cas9 genome editing

Two different E6AP-specific guide RNAs (gRNAs) were expressed from LentiCRISPRv2 (Addgene plasmid #52961) to target the *UBE3A*/*E6AP* gene in HeLa cells by the CRISPR/Cas9 method [84]. The following gRNA target sequences were used: gE6AP-1: 5′-GGTTTACTATGCAAATGTAG-3′; gE6AP-2: 5′-GAAGGATAGGTGATAGCTCAC-3′. After transfection by calcium phosphate coprecipitation [85], HeLa cells were cultivated under 1 µg/mL puromycin (Enzo Life Sciences) for selection. If indicated, nocodazole (Cayman Chemical Company), dissolved in dimethyl sulfoxide (DMSO), was added into the cell culture medium after selection with puromycin. DMSO served as solvent control. For further experimental details, please refer to the legend of S2 Fig.

### Protein analyses

Protein extraction, sodium dodecyl-sulfate polyacrylamide gel electrophoresis (SDS-PAGE), and immunoblot analyses were conducted as detailed previously [76], with the modification that protein extraction was performed in CSK-1 lysis buffer (10 nM Pipes pH 6.8, 300 M NaCl, 1 mM EDTA, 300 mM Sucrose, 1 mM MgCl_2_, 0.5% Triton X-100), supplemented with 100 µL/mL PhosSTOP phosphatase inhibitor cocktail (Roche Diagnostics), 25 µL/mL Pefabloc protease inhibitor (Merck), and 10 µL/mL protease inhibitor cocktail (Sigma-Aldrich) for 30 minutes on ice.

The following primary antibodies were used: anti-HPV18 E6 (AVC 399) and anti-HPV16 E6 (AVC 843, kind gifts from Dr. Johannes Schweizer, Arbor Vita Corporation, Fremont, CA, USA); anti-HPV18 E7 (E7C) [86]; anti-HPV16 E7 (NM2, kind gift from Dr. Martin Müller, German Cancer Research Center, Heidelberg, Germany); anti-B-MYB [87] (LX015.1, from Dr. Roger Watson; kindly provided by Dr. Kurt Engeland, University of Leipzig, Leipzig, Germany); anti-FOXM1 (D3F2B, #20549), anti-acetyl-p53 (K382) (#2525), anti-phospho-p53 (S15) (#9284), anti-phospho-p53 (S20) (#9287), anti-p130 (D9T7M, #13610), anti-pRb (#9309), anti-phospho-pRb (S807/811) (#9308), all from Cell Signaling Technology; anti-CKS1 (36-6800), from Invitrogen, Thermo Fisher Scientific; anti-β-Actin (C4, sc-47778), anti-CDC2 (B-6, sc-8395), anti-CDK2 (D-12, sc-6248), anti-Cyclin A (H-432, sc-751), anti-E2F1 (KH95, sc-251), anti-glyceraldehyde 3-phosphate dehydrogenase (GAPDH) (FL-335, sc-25778), anti-p21 (SX118, sc-53870), anti-p53 (DO-1, sc-126), anti-Vinculin (7F9, sc-73614), all from Santa Cruz Biotechnology; anti-Cyclin B1 (05–373, clone GNS3 [8A5D12]), from Upstate; anti-E6AP (E8655, clone E6AP-330), from Sigma-Aldrich.

HRP-conjugated secondary antibodies used in this study were: anti-chicken IgY-horseradish peroxidase (HRP) (sc-2428), from Santa Cruz Biotechnology; anti-mouse IgG (H + L), HRP conjugate (W4021), anti-rabbit IgG (H + L), HRP conjugate (W4011), from Promega.

Enhanced chemiluminescence (Western Bright Sirius, Advansta, or Amersham ECL Prime, Cytiva) was employed for imaging of immunoblots with the Fusion SL Detection System (Vilber Lourmat). Immunoblots were repeated at least thrice, with consistent results.

### RNA analyses

RNA extraction and quantitative reverse transcription-polymerase chain reaction (qRT-PCR) were performed as described elsewhere [88]. Primer sequences were: CCNA2 for: 5′-CCCCCAGAAGTAGCAGAGTTT-3′; CCNA2 rev: 5′-ACTTGAGGTATGGGTCAGCATC-3′; E2F1 for: 5′-GACGGCTTGAGGGGTTGAC-3′; E2F1 rev: 5′-ATGCTACGAAGGTCCTGACAC-3′; E6AP for: 5′-CGGTGGCTATACCAGGGACT-3′; E6AP rev: 5′-CTCTGTCTGTGCCCGTTGT-3′; FOXM1 for: 5′-GGCAGCAGGCTGCACTATC-3′; FOXM1 rev: 5′-TCGAAGGCTCCTCAACCTTAAC-3′; MYBL2 for: 5′-AGGCTGGCATCGAACTCATC-3′; MYBL2 rev: 5′-CTTGGGCAGTGTGGACATCA-3′; CDKN1A for: 5′-GACCATGTGGACCTGTCACT-3′; CDKN1A rev: 5′-GCGGATTAGGGCTTCCTCTT-3′; TMBIM6 for: 5′-GTGGTCATGTGTGGCTTCGT-3′; TMBIM6 rev: 5′-GGAAAGGCTGGATGGTCACT-3′.

Relative mRNA levels were quantified using the comparative C_t_$2^{-\Delta \Delta C_{t}}$method [89], with C_t_ values normalized to the internal reference gene *TMBIM6*. Subsequently, fold change values were log_2_-transformed for analysis.

### Senescence and colony formation assays

If not specified otherwise, cells were split 72 h after transfection and replated in fresh medium. For senescence assays, cells were fixed 4 days after splitting using 2% formaldehyde and 0.2% glutaraldehyde in phosphate-buffered saline (PBS). To analyze senescence-associated β-galactosidase (SA-β-Gal) activity, fixed cells were stained with 1 mg/mL X-Gal, 5 mM K_3_[Fe(CN)_6_], 5 mM K_4_[Fe(CN)_6_] in 40 mM citric acid, 150 mM NaCl, and 2 mM MgCl_2_, pH 6.0, for 24 h at 37 °C. Imaging of cells was performed using the EVOSxI Core Cell Imaging System (Invitrogen, Thermo Fisher Scientific) with 20-fold magnification. For colony formation assays, the cell culture medium was exchanged every 3 to 4 days and cells were fixed and stained with formaldehyde-cristal violet 10 to 17 days after splitting. All senescence and colony formation assays were conducted at least thrice, with consistent results.

### Cell cycle analyses

For cell cycle analyses, cells were trypsinized, washed in cold PBS, and fixed 72 h after transfection with 75% ice-cold ethanol at -20 °C overnight. Subsequently, cells were pelleted, stained with PBS containing 25 µg/mL propidium iodide (Sigma-Aldrich) in the presence of 500 µg/mL RNase A (Roche Diagnostics), and incubated for 30 minutes at room temperature. If indicated, nocodazole, dissolved in DMSO, was added into the cell culture medium during the final 16 h (HeLa) or 24 h (SiHa) of the respective experiment at a final concentration of 0.1 µg/mL. DMSO served as solvent control.

Cell cycle profiles were assessed by flow cytometry using the BD LSRFortessa (BD Biosciences) and the BD FACS DIVA Software version v8.0.1. Flow cytometry data were analyzed using FlowJo v10.8.1 Software (BD Life Sciences) and the Dean-Jett-Fox model was applied to quantify cells in individual cell cycle phases [90]. Cell cycle analyses were performed thrice, with consistent results.

### Live-cell imaging

For live-cell imaging, mKate2-labeled cells were reverse transfected and seeded at a density of 2,000 to 4,000 cells per well into 96-well plates. Beginning 24 h post-transfection, imaging was performed every 6 h at a 10-fold magnification using the Incucyte S3 device (Sartorius). Proliferation curves were generated by analyzing viable cell counts (red objects) with the Incucyte Software (v2021C). Live-cell imaging experiments were performed at least thrice in triplicates, with consistent results.

### Terminal deoxynucleotidyl transferase-mediated UTP end labeling (TUNEL) assays

For TUNEL analyses, the “In Situ Cell Death Detection Kit” (Roche) was used as per the manufacturer’s protocol. Imaging was performed using a Zeiss Cell Observer Microscope (Zeiss) at a 20-fold magnification. TUNEL positive cells and total cell counts (determined by 4`,6-diamidino-2-phenylindole [DAPI] staining, Roche) were counted from a minimum of 5 images per condition using a Fiji/ImageJ25 macro (kindly provided by Dr. Damir Krunic, Light Microscopy Core Facility, German Cancer Research Center) [72,88].

### Statistical analyses

Statistical analyses were performed using GraphPad Prism 10.1.2 (GraphPad Software Inc.). One-way analysis of variance (ANOVA), followed by Sidak’s multiple comparisons test, or two-way ANOVA, followed by Tukey’s multiple comparisons test, were conducted to determine statistical significance, indicated as * *p* ≤ 0.05, ** *p* ≤ 0.01, and *** *p* ≤ 0.001.

## Supporting information

S1 Fig Downregulation of E6AP does not induce senescence in HPV-negative cancer cells. RKO, HCT116, or U2OS cells were transfected with siE6AP or control siRNA (siNeg). Cells were examined following the treatment scheme depicted in Fig 1A by **(A)** senescence assays (upper panels; SA-β-Gal staining; scale bar: 200 µm) and corresponding CFAs (lower panels), and by **(B)** immunoblot analyses for E6AP, p53, p21, and GAPDH protein levels.
(TIF)

S2 Fig Interfering with E6AP expression by CRISPR/Cas9 genome editing induces senescence in HeLa cells. **(A)** Treatment scheme: HeLa cells were transfected (Tx) with control plasmid LentiCRISPRv2, or with LentiCRISPRv2 expressing either gE6AP-1 or gE6AP-2. For selection of transfected cells, 1 µg/mL puromycin was added into the cell culture medium 24 h post-transfection for 48 h. To eliminate proliferating cells, cells were treated with nocodazole, when indicated, and cultivated for another 72 h. DMSO served as solvent control (-). 6 days after transfection, cells were either harvested for protein analyses or investigated for senescence induction by SA-β-Gal staining assays. **(B)** Corresponding senescence assays (SA-β-Gal staining, blue; scale bar: 200 µm). **(C)** Immunoblot analyses of E6AP, p53, p21, 18E6, 18E7, and GAPDH protein levels. Vertical lines between the lanes indicate where original images from the same blot were spliced for the purpose of presentation. Please note the emergence of senescent cells upon transfection with LentiCRISPRv2 expressing either gE6AP-1 or gE6AP-2, but not upon transfection with control plasmid LentiCRISPRv2 (S2B Fig, upper panels). For further analyses, senescent cells were enriched by eliminating proliferating cells through nocodazole treatment [91] (S2B Fig, lower panels). As observed for RNAi-mediated E6AP repression (please refer to the main text), E6AP repression was linked to senescence induction (S2B Fig), concomitant downregulation of E6 levels, maintenance of E7, and a strong increase in p53 and p21 levels (S2C Fig).
(TIF)

S3 Fig The senescence response of SiHa cells is not enhanced by increasing siE6 concentrations or by repeated siE6 transfections. **(A)** Treatment scheme: SiHa cells were transfected with either 10 nM or 30 nM si16E6 or control siRNA (siNeg). 48 h after the first transfection (Tx 1), half of the samples were transfected a second time with the same siRNAs (Tx 2). 96 h post-transfection, cells were either harvested for protein (Prot) analyses, or split and further cultivated for senescence assays (SA-β-Gal staining) or colony formation assays (CFAs) after the indicated time periods. **(B)** Immunoblot analyses of P-pRb Ser807/811, total pRb, E6AP, 16E6, 16E7, and β-Actin protein levels following transfection with the indicated amounts of siRNAs, performed either once (1x) or twice (2x). **(C)** Corresponding senescence assays (upper panels; SA-β-Gal staining, blue; scale bar: 200 µm) and CFAs (lower panels).
(TIF)

S4 Fig Senescence induction upon downregulation of E6AP or E6/E7 expression is counteracted by repression of p130 or pRb in HeLa cells. HeLa cells were transfected with siE6AP, si18E6/E7, or control siRNA (siNeg), either alone or in combination with sip130, sipRb, or a combination of both sip130/sipRb, as indicated. Senescence assays (SA-β-Gal staining, blue; scale bar: 200 µm) were performed 7 days after transfection.
(TIF)

S5 Fig Schematic overview of siRNA target sites. **(A)** siE6AP-1, -2, -3 target distinct exons of the *UBE3A/E6AP* gene. The reference transcript NM_000462.5, representing the transcript variant producing the longest protein isoform, was used to illustrate the location of the siRNA target sites. The pool of all three siRNAs targets all known isoforms of E6AP. For individual siRNA sequences, please refer to the materials and methods section. **(B, C)** Schematic representation of **(B)** HPV18 and **(C)** HPV16 transcripts coding for E6 and E7 [92,93]. Boxes represent coding regions, intron sequences are indicated, and main translated products are shown on the right. The target sites for 18E6 siRNAs (si18E6-1, -2, -3), 18E6/E7 siRNAs (si18E6/E7-1, -2, -3), 16E6 siRNAs (si16E6-1, -2, -3), and 16E6/E7 siRNAs (si16E6/E7-1, -2, -3) are illustrated. Type 1 transcripts produce E6, but little or no E7, due to inefficient translational reinitiation. Type 2 transcripts (and type 3 transcripts for HPV16) produce E7 [92,93]. Consequently, siRNAs targeting the intronic E6 sequences selectively suppress E6, whereas siRNAs targeting sequences present in all transcript classes suppress both E6 and E7. For individual siRNA sequences, please refer to the materials and methods section. Created with BioRender.com.
(TIF)

S1 Data Numerical data underlying the graphs presented in the study. (XLSX)

S1 Raw images Original images of western blots for Figs 1 and 4. For each blot, two images are presented: (1) a marker overlay image indicating molecular weight (kDa) positions based on a protein marker, and (2) the exposures of the blots used for the individual figures. Red boxes indicate the crops used for the figures.
(PDF)

S2 Raw images Original images of western blots for Figs 5 and 7 and 8. For each blot, two images are presented: (1) a marker overlay image indicating molecular weight (kDa) positions based on a protein marker, and (2) the exposures of the blots used for the individual figures. Red boxes indicate the crops used for the figures.
(PDF)

S3 Raw images Original images of western blots for S1B, S2C, and S3B Figs. For each blot, two images are presented: (1) a marker overlay image indicating molecular weight (kDa) positions based on a protein marker, and (2) the exposures of the blots used for the individual figures. Red boxes indicate the crops used for the figures.
(PDF)
