# Supplementary figures and images for: E6AP is essential for the proliferation of HPV-positive cancer cells by preventing senescence
Source: PLoS Pathog. 2025 Feb 7;21(2):e1012914. doi: 10.1371/journal.ppat.1012914 (PMC11805377; doi:10.1371/journal.ppat.1012914)

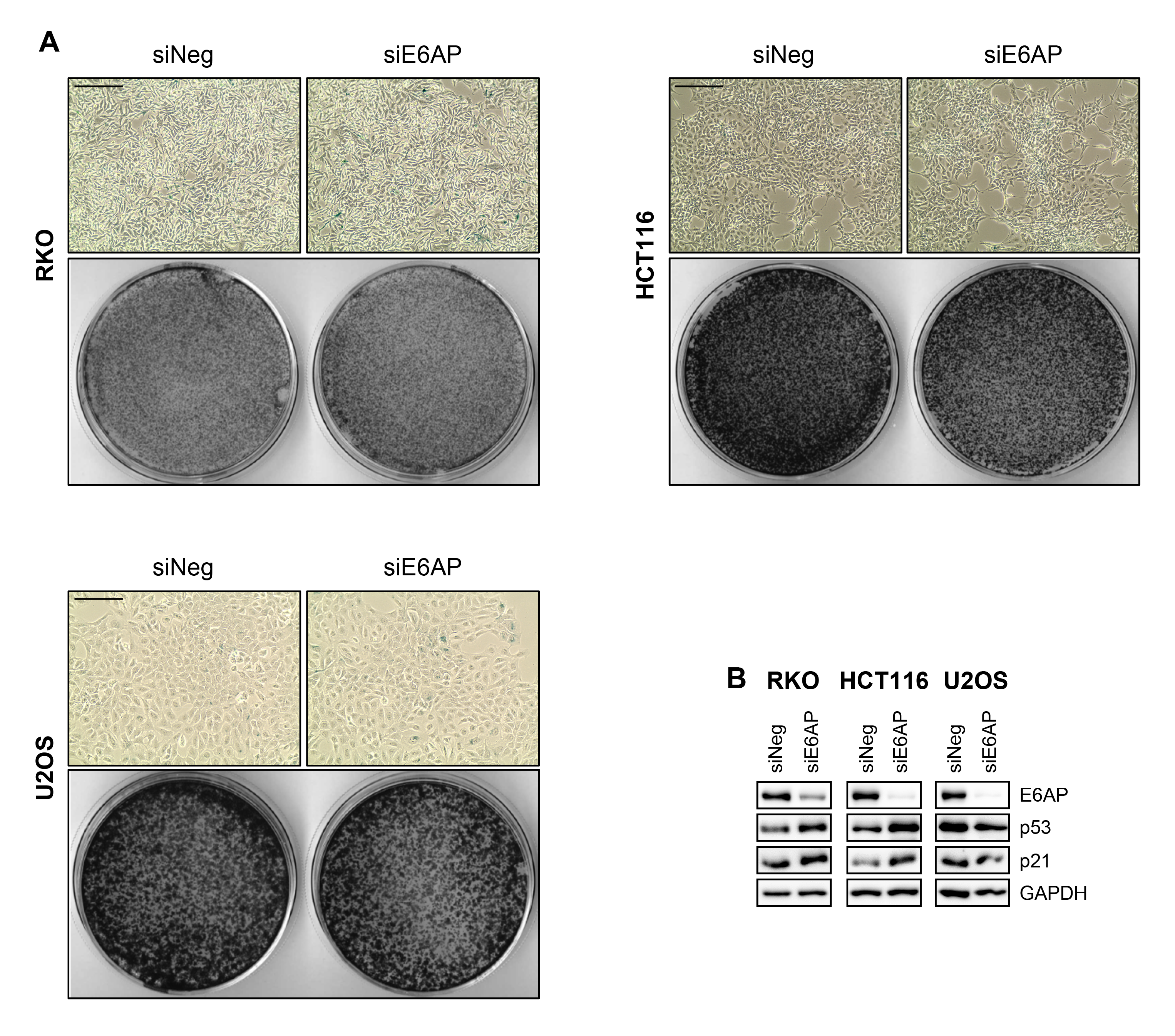

Supplement: S1 Fig — RKO, HCT116, or U2OS cells were transfected with siE6AP or control siRNA (siNeg). Cells were examined following the treatment scheme depicted in Fig 1A by (A) senescence assays (upper panels; SA-β-Gal staining; scale bar: 200 µm) and corresponding CFAs (lower panels), and by (B) immunoblot analyses for E6AP, p53, p21, and GAPDH protein levels. (TIF) [file ppat.1012914.s001.tif]

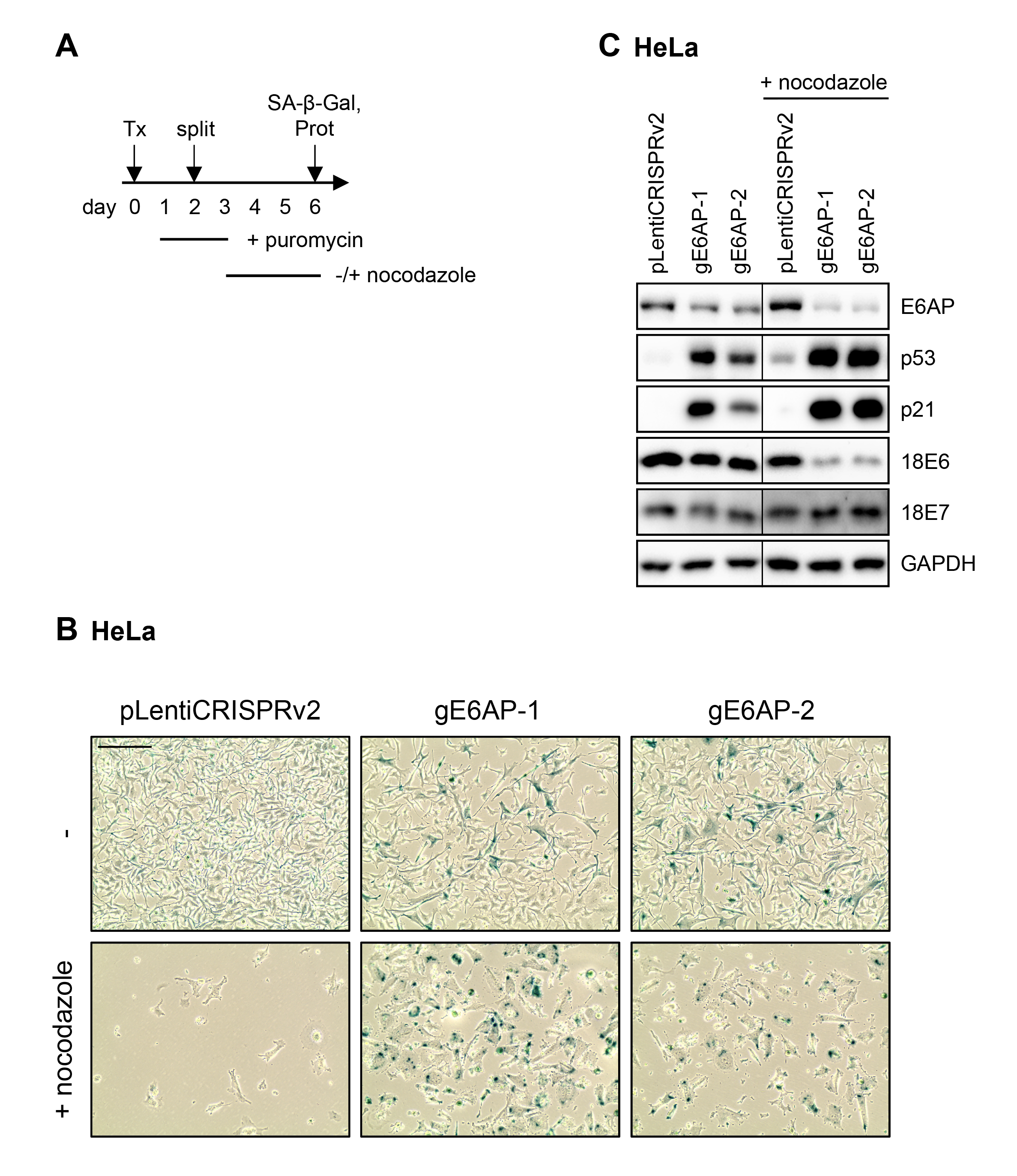

Supplement: S2 Fig — (A) Treatment scheme: HeLa cells were transfected (Tx) with control plasmid LentiCRISPRv2, or with LentiCRISPRv2 expressing either gE6AP-1 or gE6AP-2. For selection of transfected cells, 1 µg/mL puromycin was added into the cell culture medium 24 h post-transfection for 48 h. To eliminate proliferating cells, cells were treated with nocodazole, when indicated, and cultivated for another 72 h. DMSO served as solvent control (-). 6 days after transfection, cells were either harvested for protein analyses or investigated for senescence induction by SA-β-Gal staining assays. (B) Corresponding senescence assays (SA-β-Gal staining, blue; scale bar: 200 µm). (C) Immunoblot analyses of E6AP, p53, p21, 18E6, 18E7, and GAPDH protein levels. Vertical lines between the lanes indicate where original images from the same blot were spliced for the purpose of presentation. Please note the emergence of senescent cells upon transfection with LentiCRISPRv2 expressing either gE6AP-1 or gE6AP-2, but not upon transfection with control plasmid LentiCRISPRv2 (S2B Fig, upper panels). For further analyses, senescent cells were enriched by eliminating proliferating cells through nocodazole treatment [91] (S2B Fig, lower panels). As observed for RNAi-mediated E6AP repression (please refer to the main text), E6AP repression was linked to senescence induction (S2B Fig), concomitant downregulation of E6 levels, maintenance of E7, and a strong increase in p53 and p21 levels (S2C Fig). (TIF) [file ppat.1012914.s002.tif]

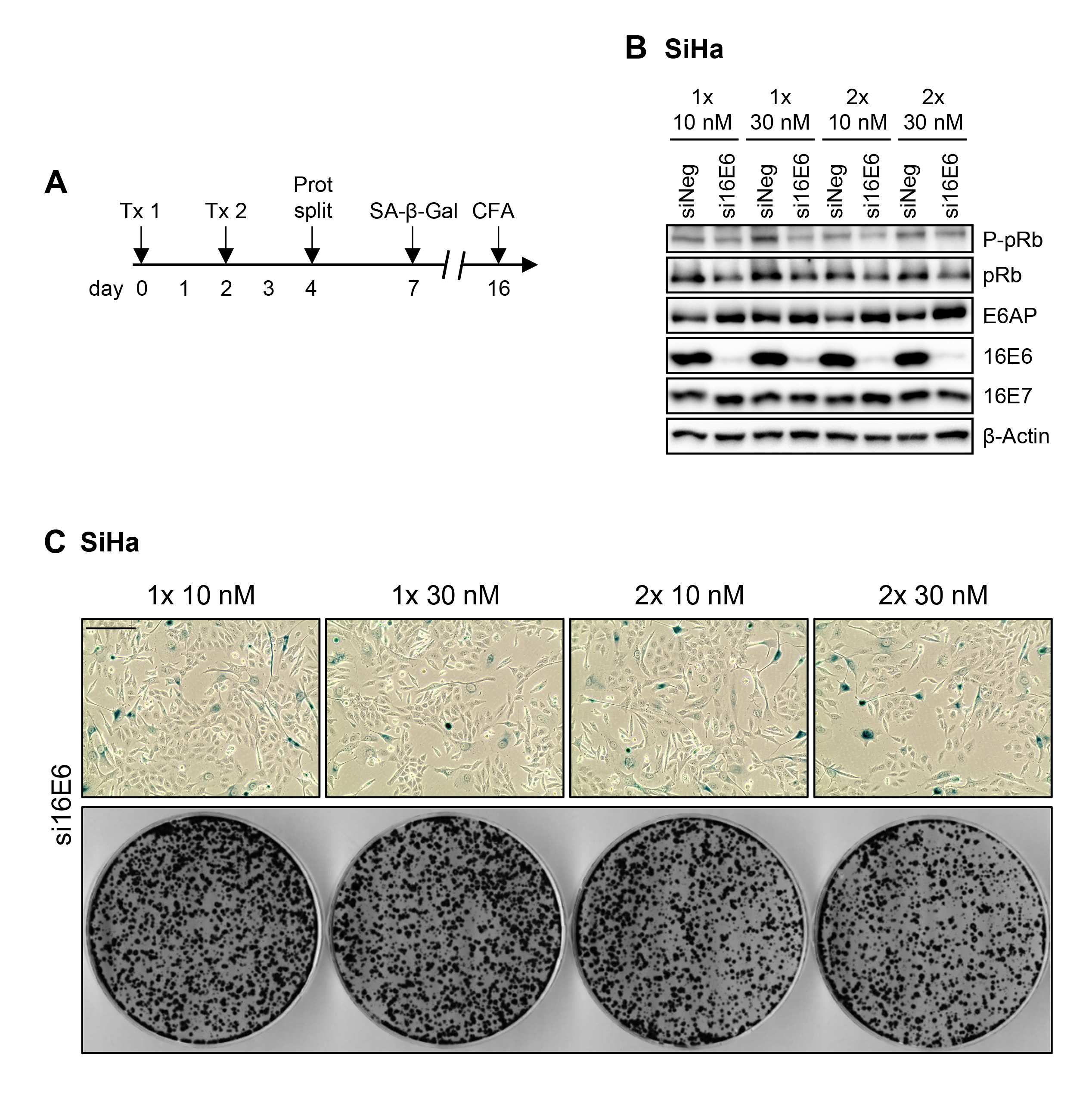

Supplement: S3 Fig — (A) Treatment scheme: SiHa cells were transfected with either 10 nM or 30 nM si16E6 or control siRNA (siNeg). 48 h after the first transfection (Tx 1), half of the samples were transfected a second time with the same siRNAs (Tx 2). 96 h post-transfection, cells were either harvested for protein (Prot) analyses, or split and further cultivated for senescence assays (SA-β-Gal staining) or colony formation assays (CFAs) after the indicated time periods. (B) Immunoblot analyses of P-pRb Ser807/811, total pRb, E6AP, 16E6, 16E7, and β-Actin protein levels following transfection with the indicated amounts of siRNAs, performed either once (1x) or twice (2x). (C) Corresponding senescence assays (upper panels; SA-β-Gal staining, blue; scale bar: 200 µm) and CFAs (lower panels). (TIF) [file ppat.1012914.s003.tif]

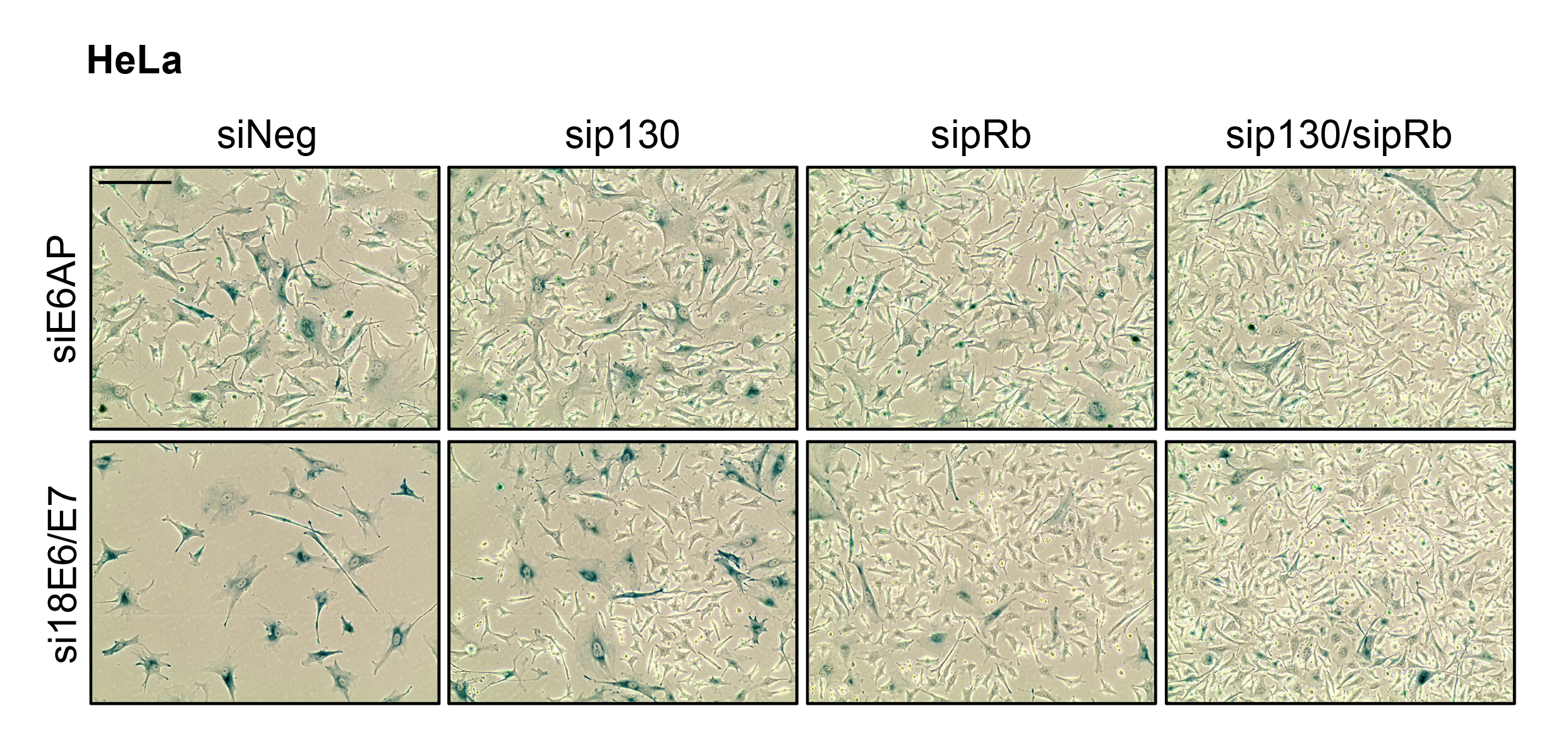

Supplement: S4 Fig — HeLa cells were transfected with siE6AP, si18E6/E7, or control siRNA (siNeg), either alone or in combination with sip130, sipRb, or a combination of both sip130/sipRb, as indicated. Senescence assays (SA-β-Gal staining, blue; scale bar: 200 µm) were performed 7 days after transfection. (TIF) [file ppat.1012914.s004.tif]

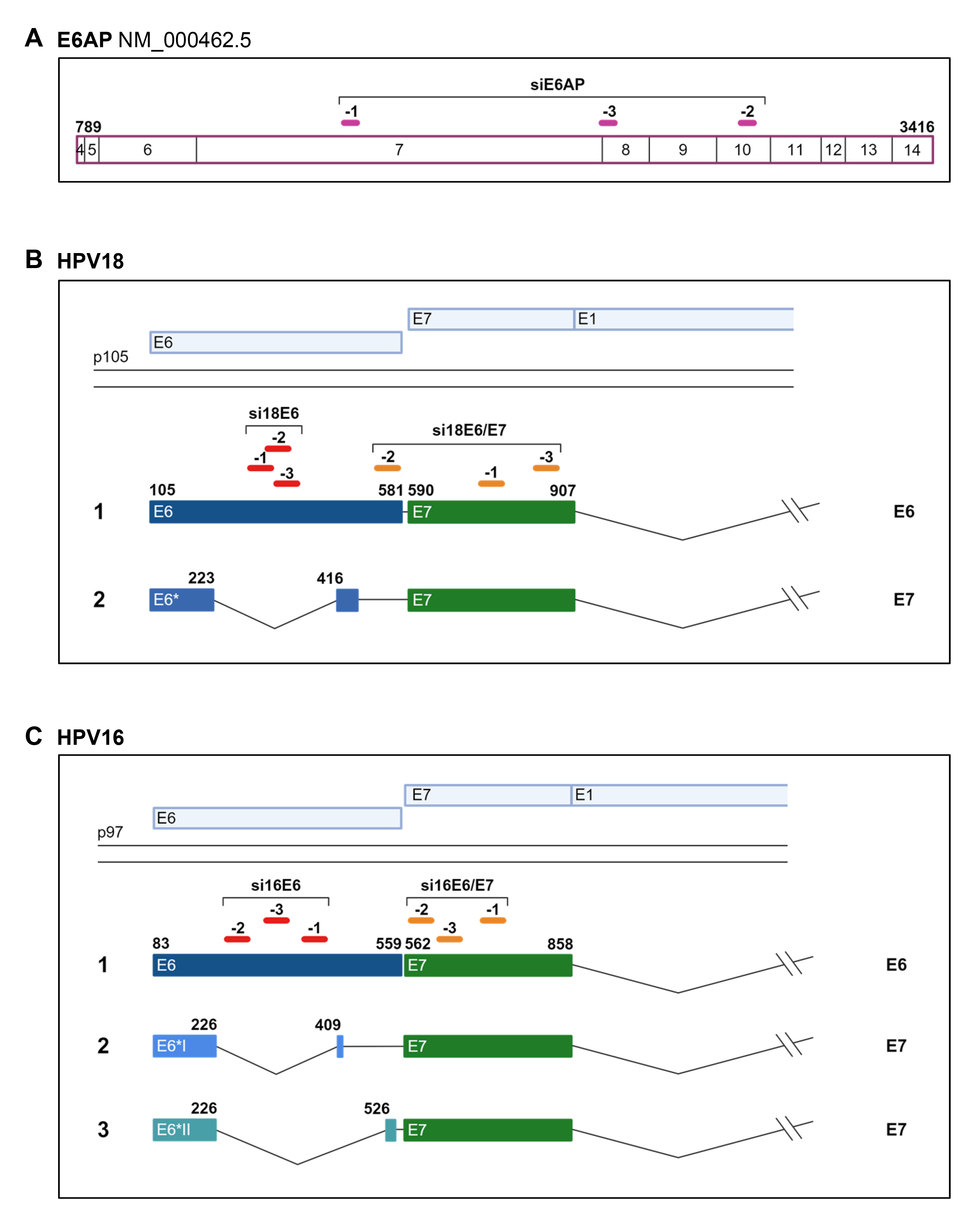

Supplement: S5 Fig — (A) siE6AP-1, -2, -3 target distinct exons of the UBE3A/E6AP gene. The reference transcript NM_000462.5, representing the transcript variant producing the longest protein isoform, was used to illustrate the location of the siRNA target sites. The pool of all three siRNAs targets all known isoforms of E6AP. For individual siRNA sequences, please refer to the materials and methods section. (B, C) Schematic representation of (B) HPV18 and (C) HPV16 transcripts coding for E6 and E7 [92,93]. Boxes represent coding regions, intron sequences are indicated, and main translated products are shown on the right. The target sites for 18E6 siRNAs (si18E6-1, -2, -3), 18E6/E7 siRNAs (si18E6/E7-1, -2, -3), 16E6 siRNAs (si16E6-1, -2, -3), and 16E6/E7 siRNAs (si16E6/E7-1, -2, -3) are illustrated. Type 1 transcripts produce E6, but little or no E7, due to inefficient translational reinitiation. Type 2 transcripts (and type 3 transcripts for HPV16) produce E7 [92,93]. Consequently, siRNAs targeting the intronic E6 sequences selectively suppress E6, whereas siRNAs targeting sequences present in all transcript classes suppress both E6 and E7. For individual siRNA sequences, please refer to the materials and methods section. Created with BioRender.com. (TIF) [file ppat.1012914.s005.tif]
