# Supplementary material for: E6AP is essential for the proliferation of HPV-positive cancer cells by preventing senescence
Source: PLoS Pathog. 2025 Feb 7;21(2):e1012914. doi: 10.1371/journal.ppat.1012914 (PMC11805377; doi:10.1371/journal.ppat.1012914)

Fig 1B HeLa

E6AP

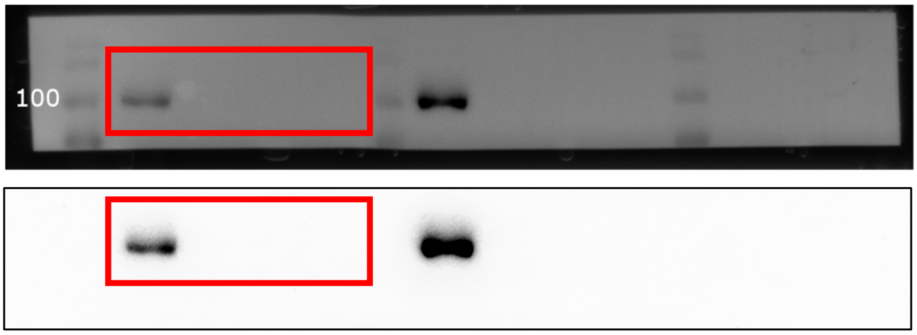

18E6

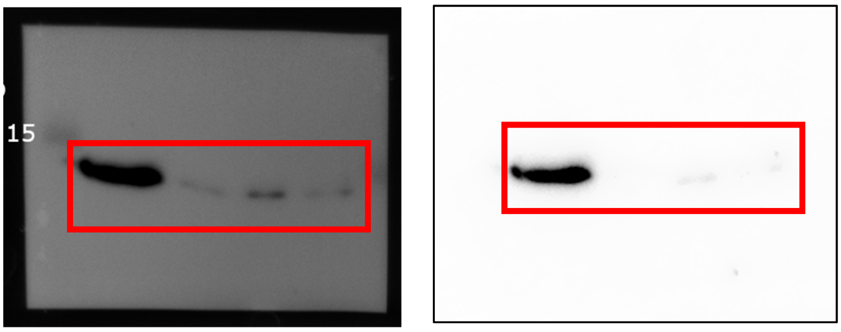

18E7

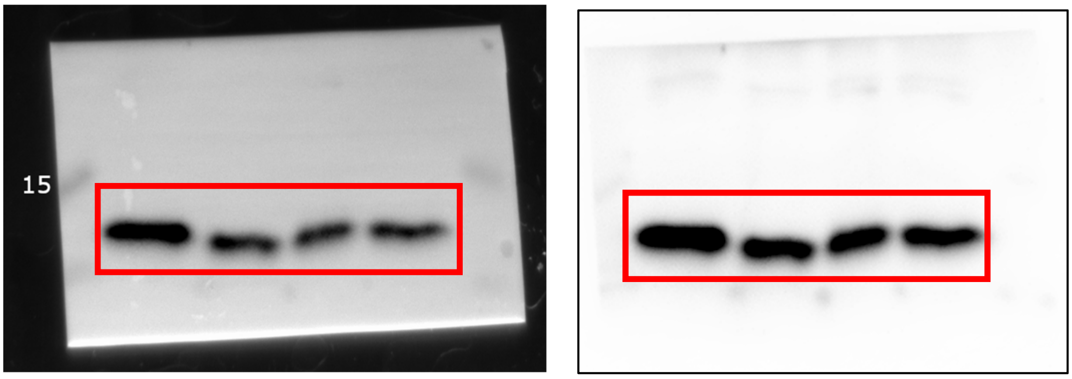

p53

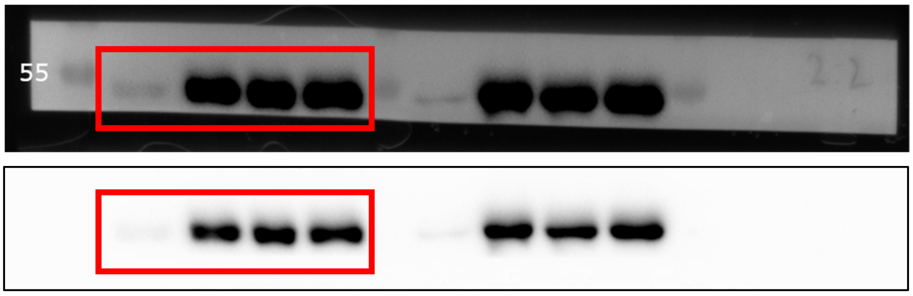

Fig 1B HeLa

p21

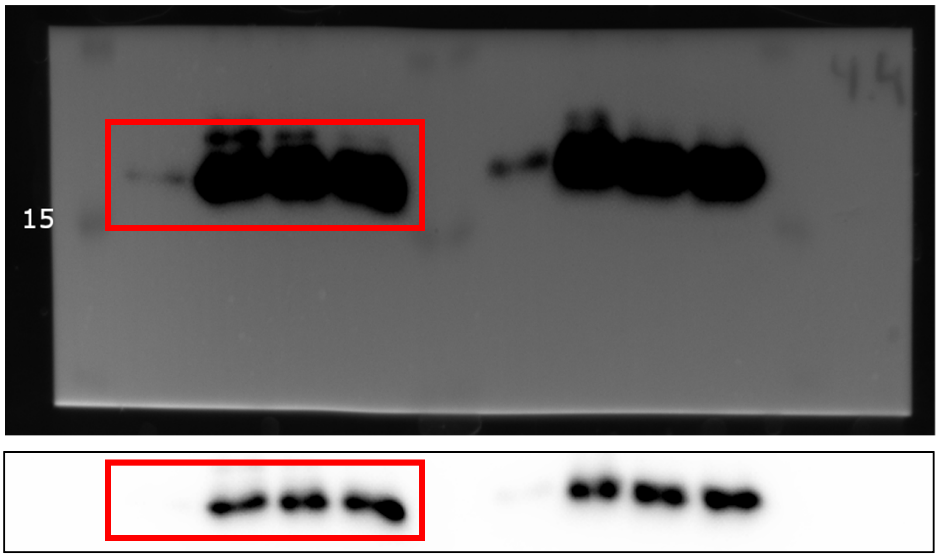

Vinculin

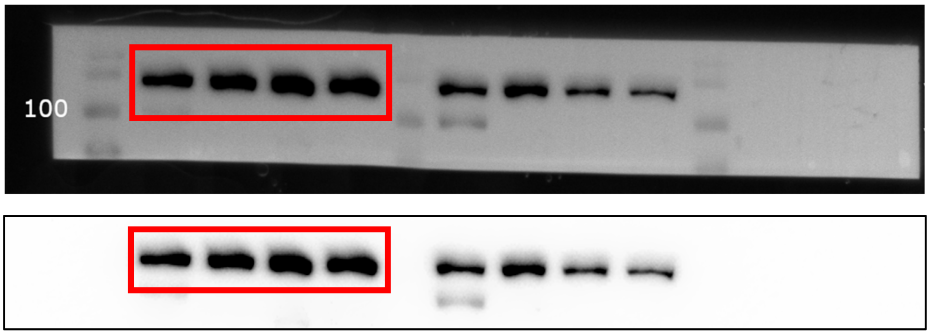

Fig 1B SiHa

E6AP

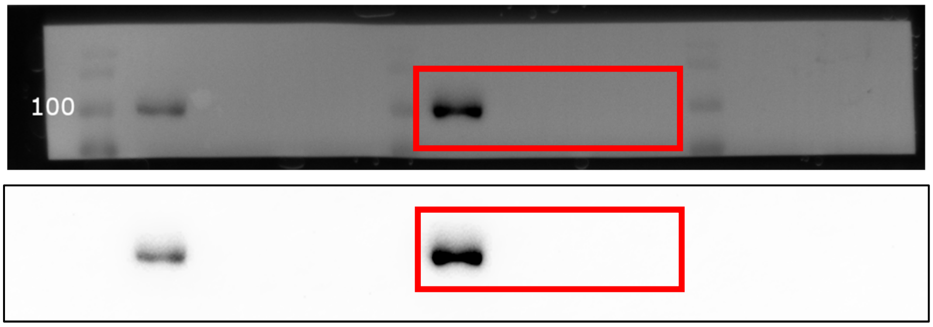

16E6

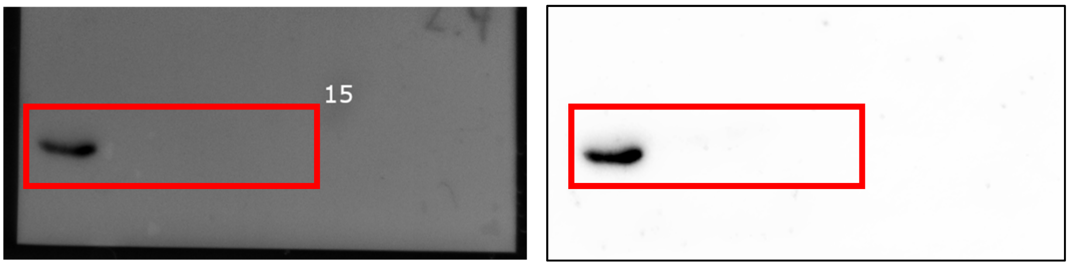

16E7

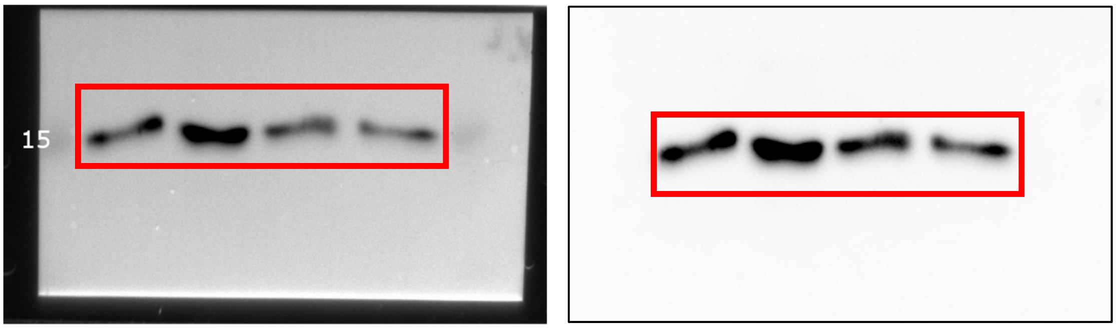

p53

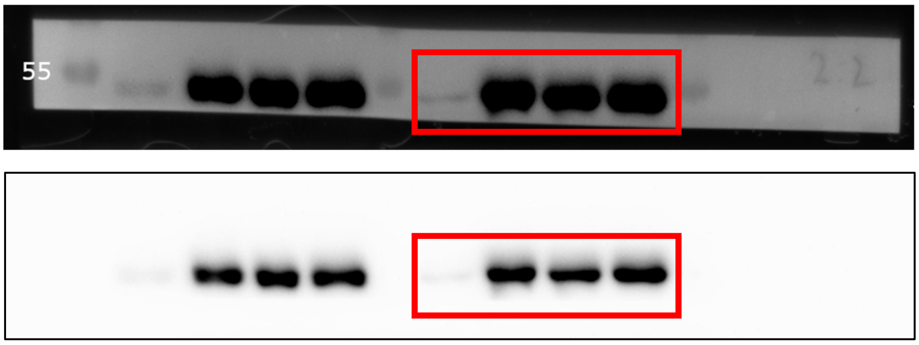

Fig 1B SiHa

p21

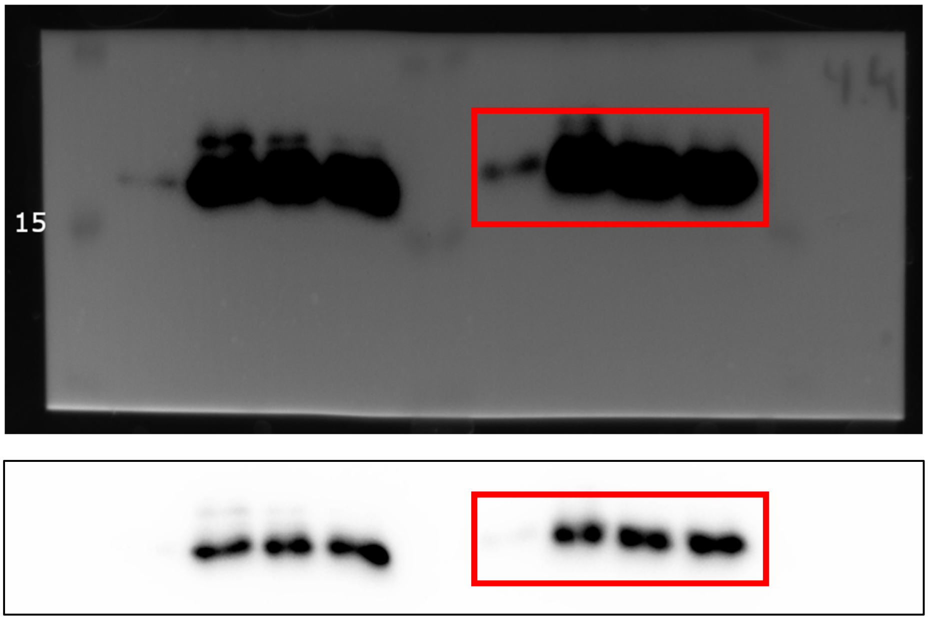

Vinculin

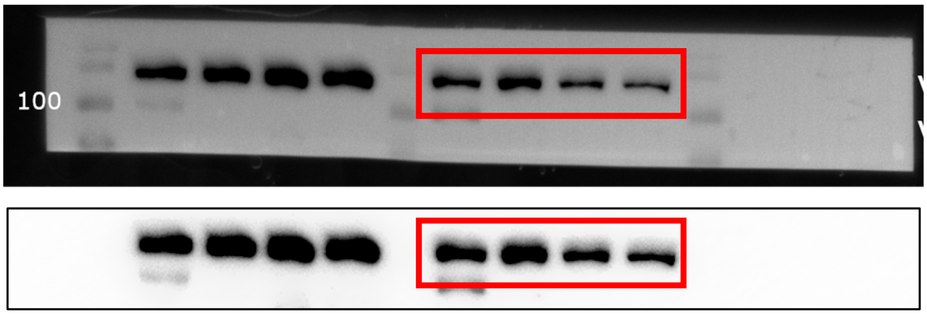

Fig 4C HeLa

B-MYB

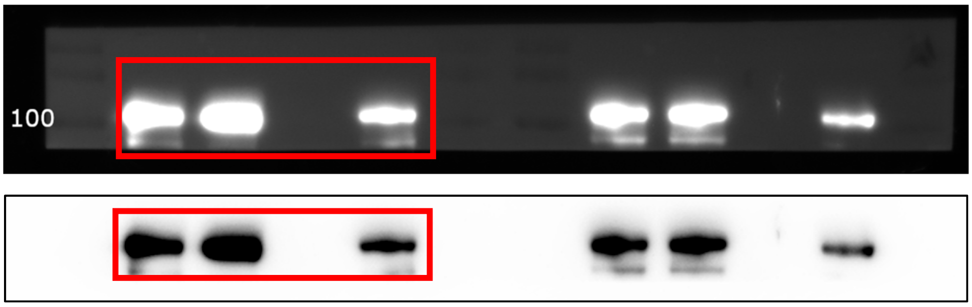

FOXM1

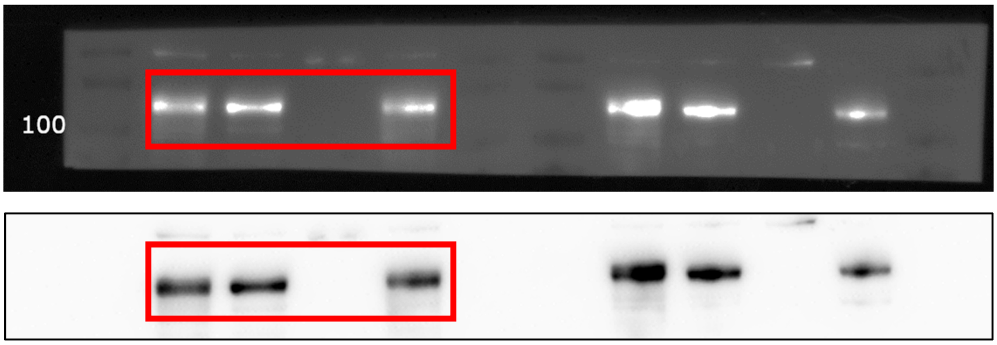

E2F1

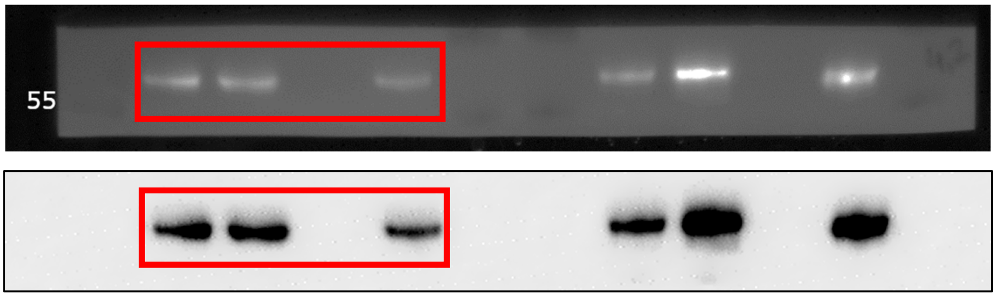

Cyclin A

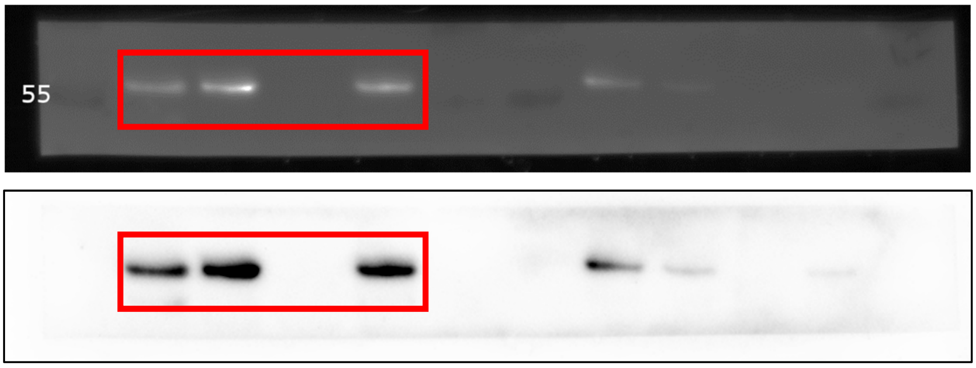

Fig 4C HeLa

Cyclin B1

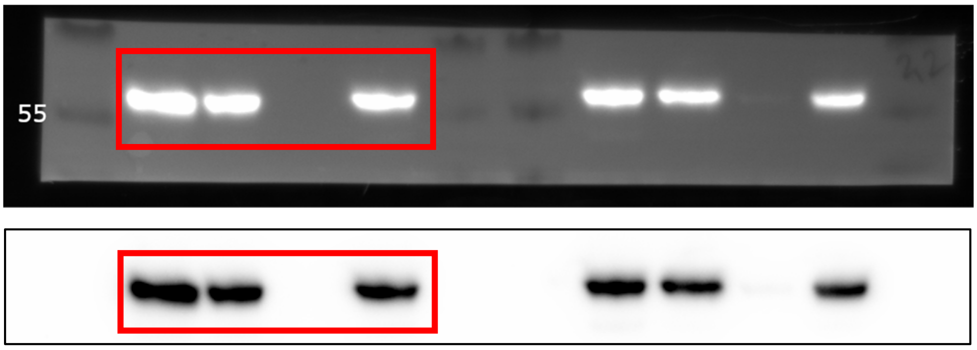

CDC2

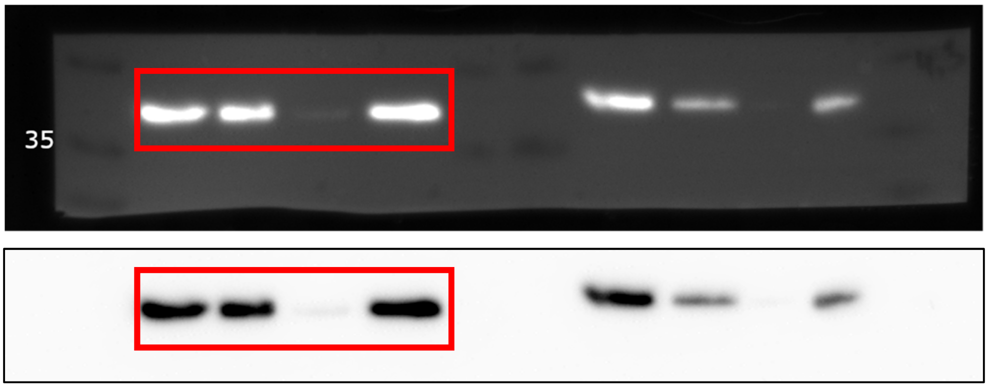

CDK2

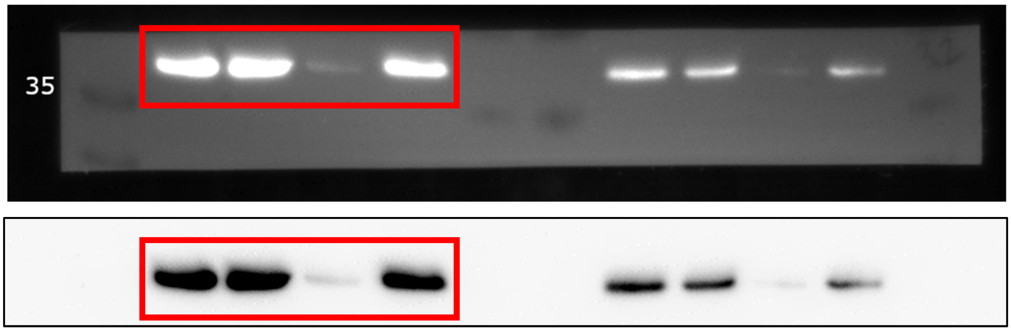

CKS1

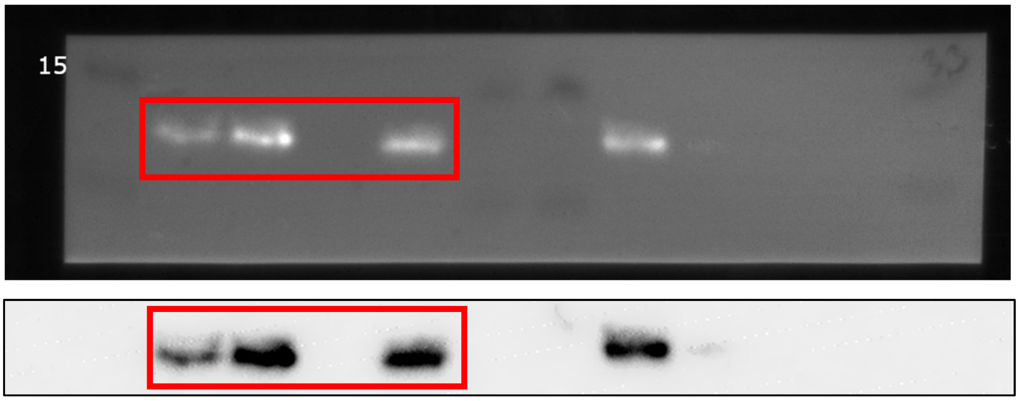

Fig 4C HeLa

E6AP

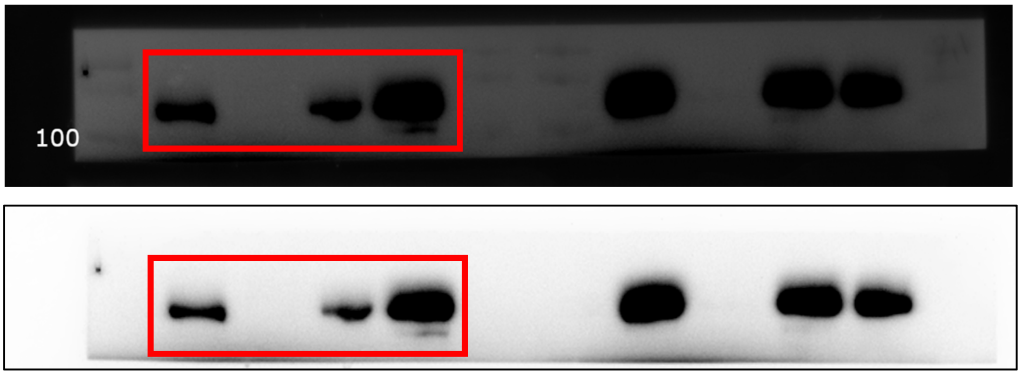

18E6

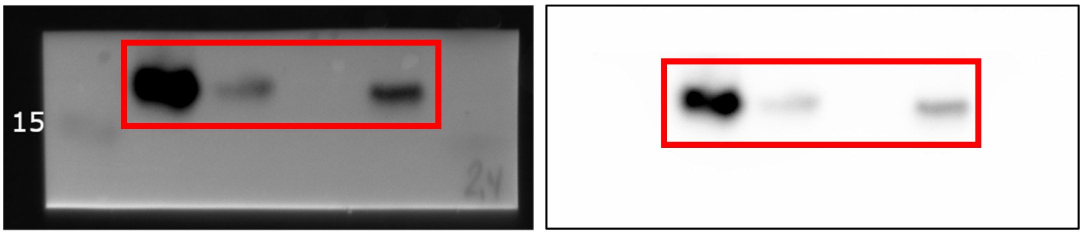

18E7

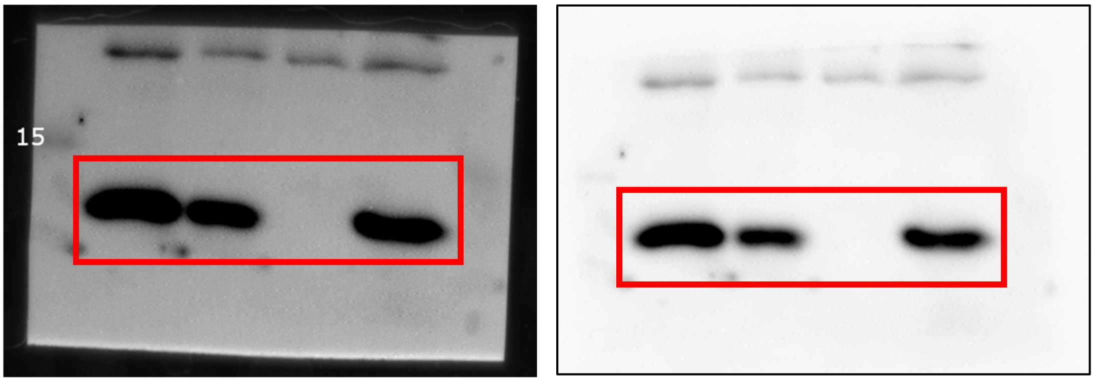

GAPDH

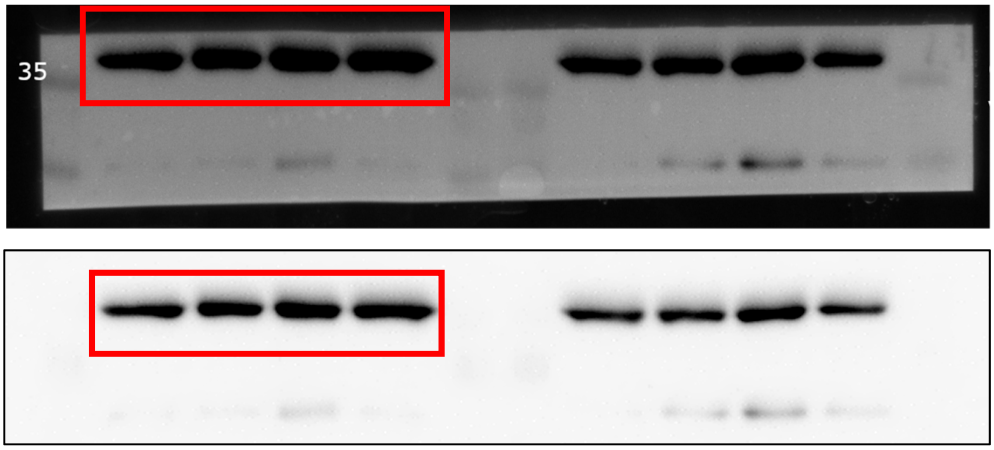

Fig 4C SiHa

B-MYB

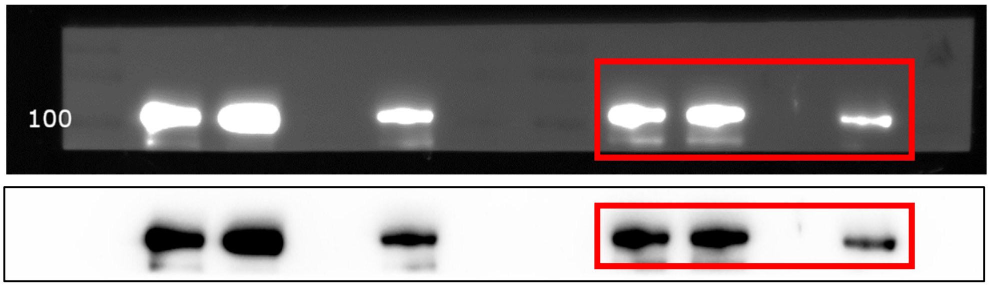

FOXM1

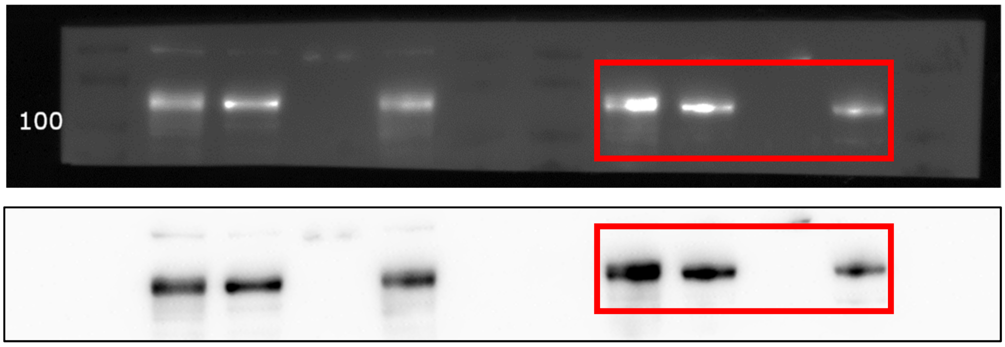

E2F1

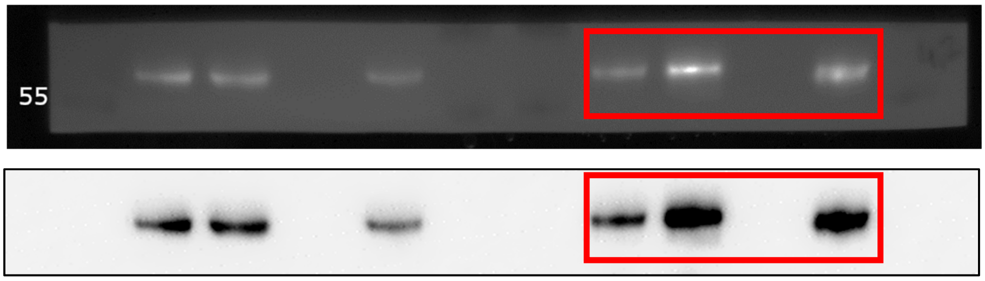

Cyclin A

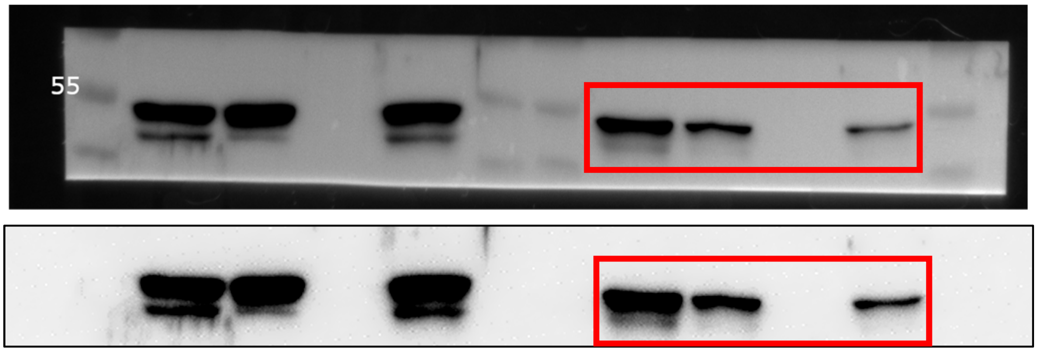

Fig 4C SiHa

Cyclin B1

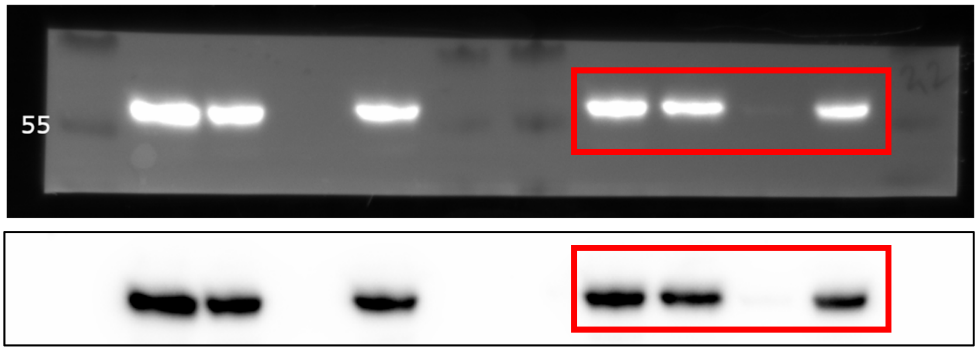

CDC2

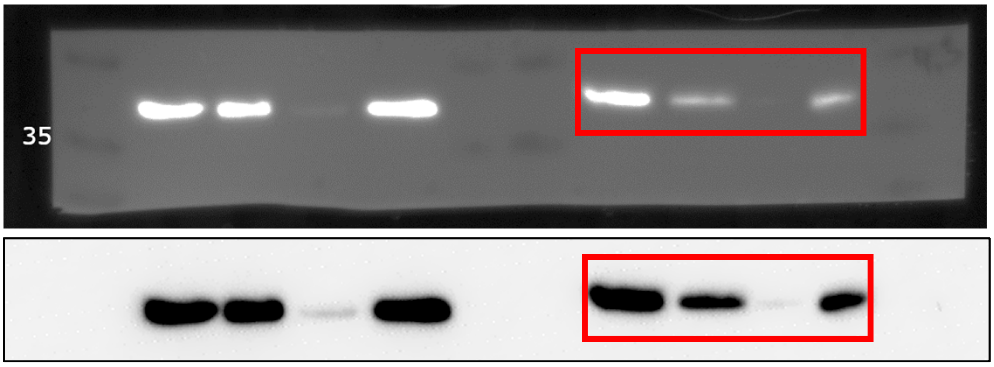

CDK2

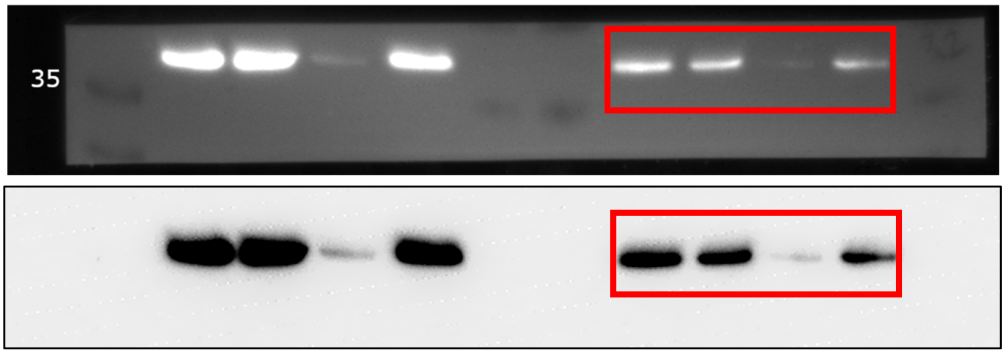

CKS1

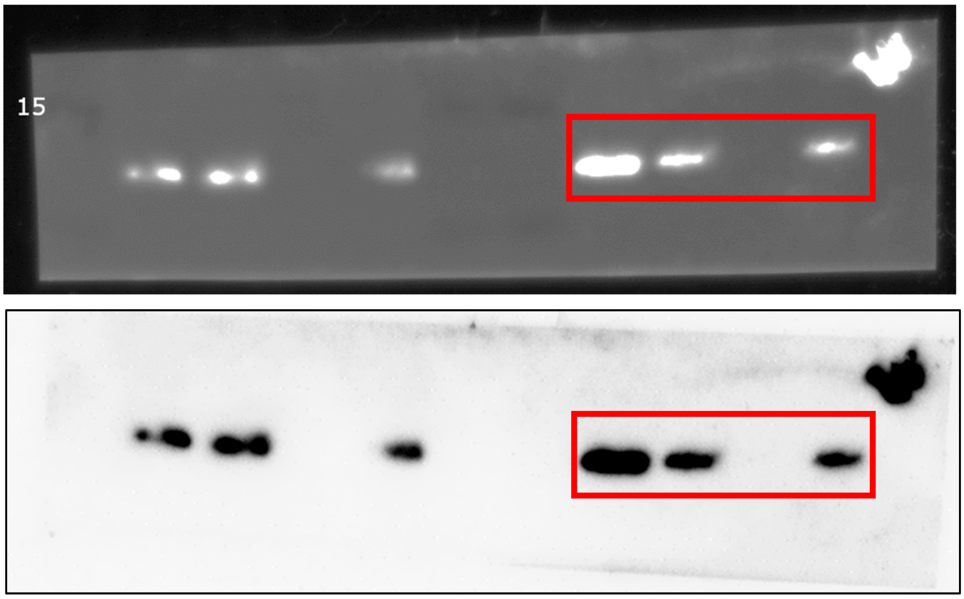

Fig 4C SiHa

E6AP

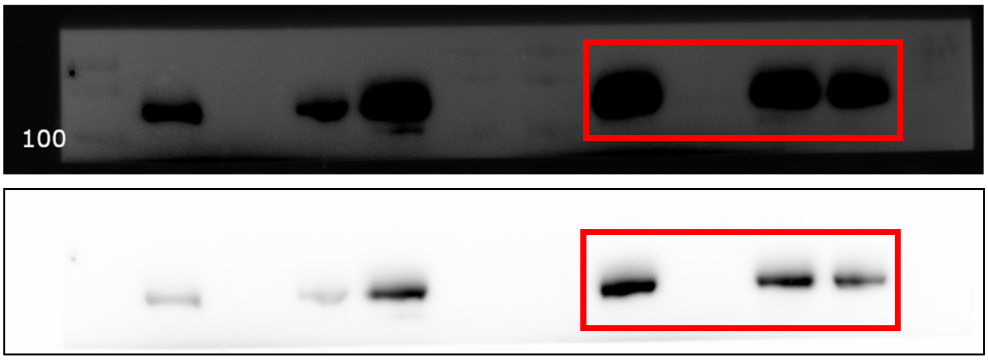

16E6

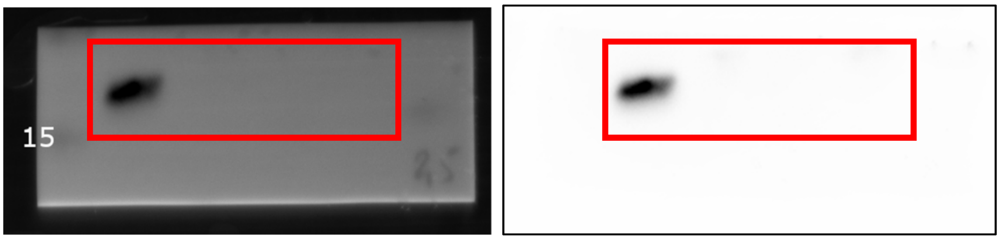

16E7

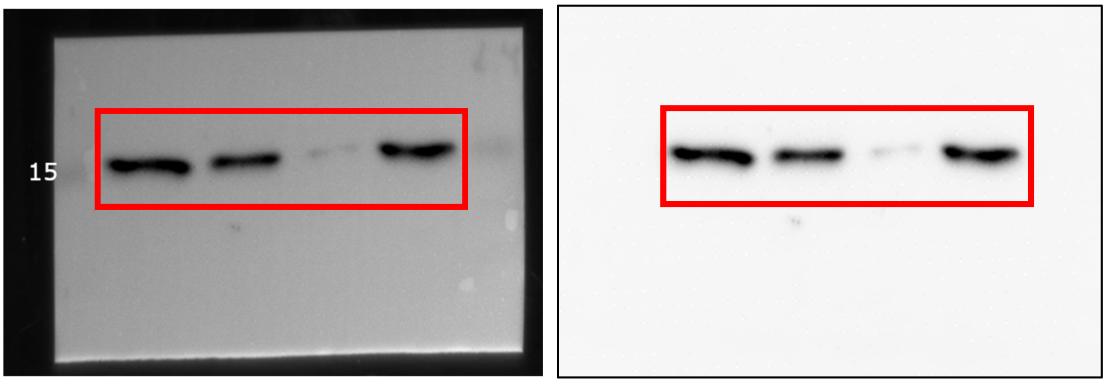

GAPDH

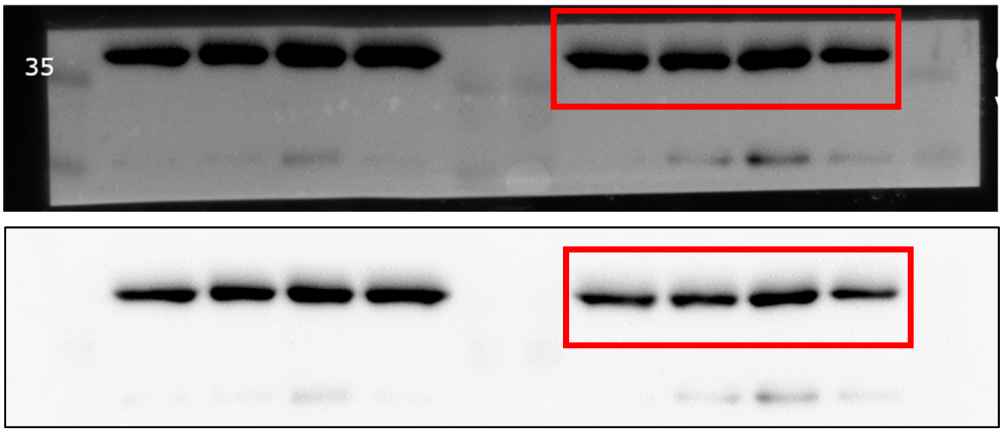

Supplement: S1 Raw images — For each blot, two images are presented: (1) a marker overlay image indicating molecular weight (kDa) positions based on a protein marker, and (2) the exposures of the blots used for the individual figures. Red boxes indicate the crops used for the figures. (PDF) [file ppat.1012914.s007.pdf]
