# Supplementary material for: E6AP is essential for the proliferation of HPV-positive cancer cells by preventing senescence
Source: PLoS Pathog. 2025 Feb 7;21(2):e1012914. doi: 10.1371/journal.ppat.1012914 (PMC11805377; doi:10.1371/journal.ppat.1012914)

Fig 5A HeLa

p53

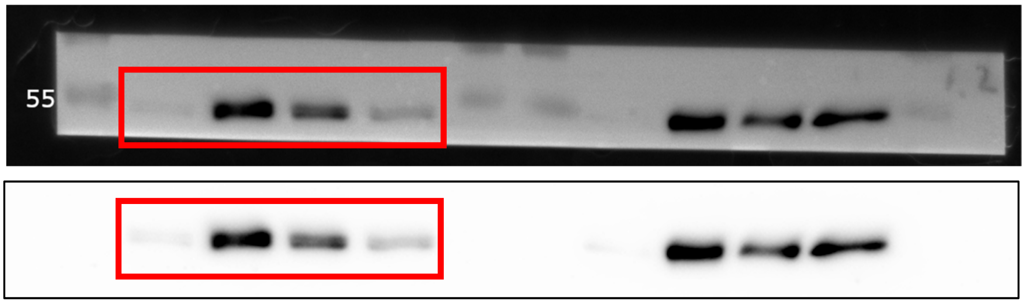

P-p53 Ser15

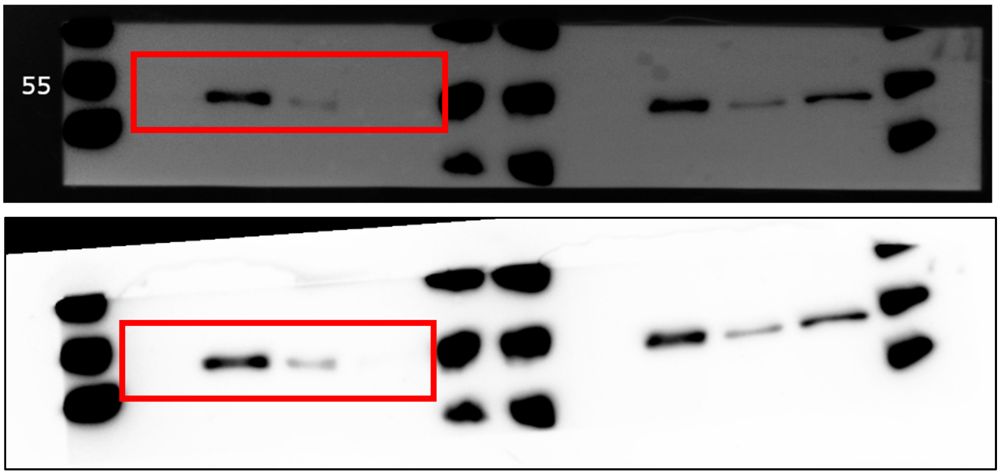

P-p53 Ser20

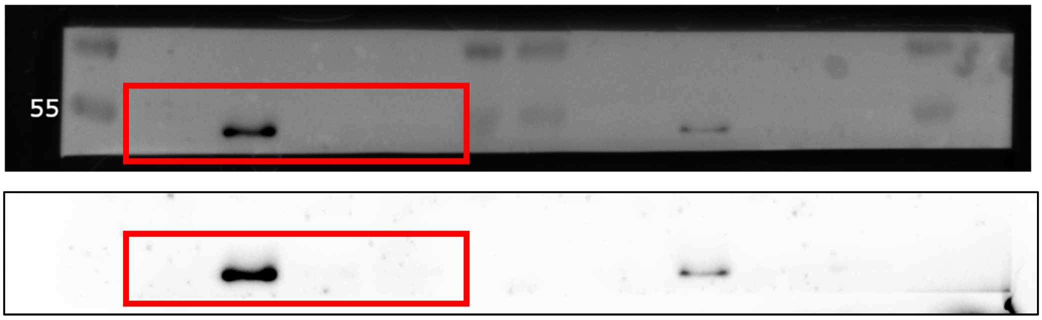

Ac-p53

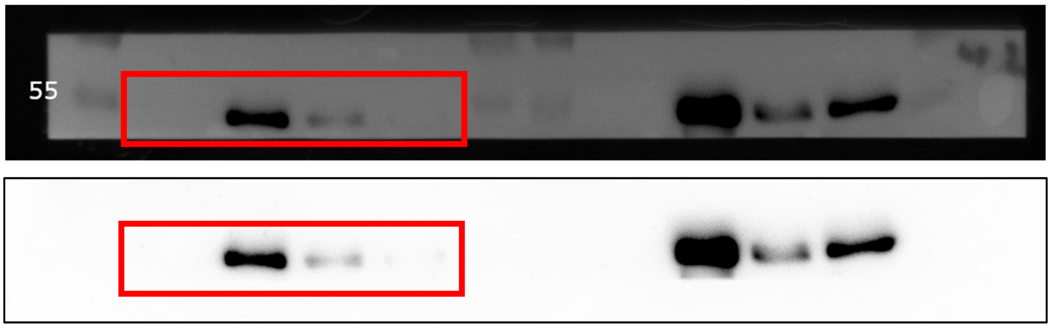

Fig 5A HeLa

p21

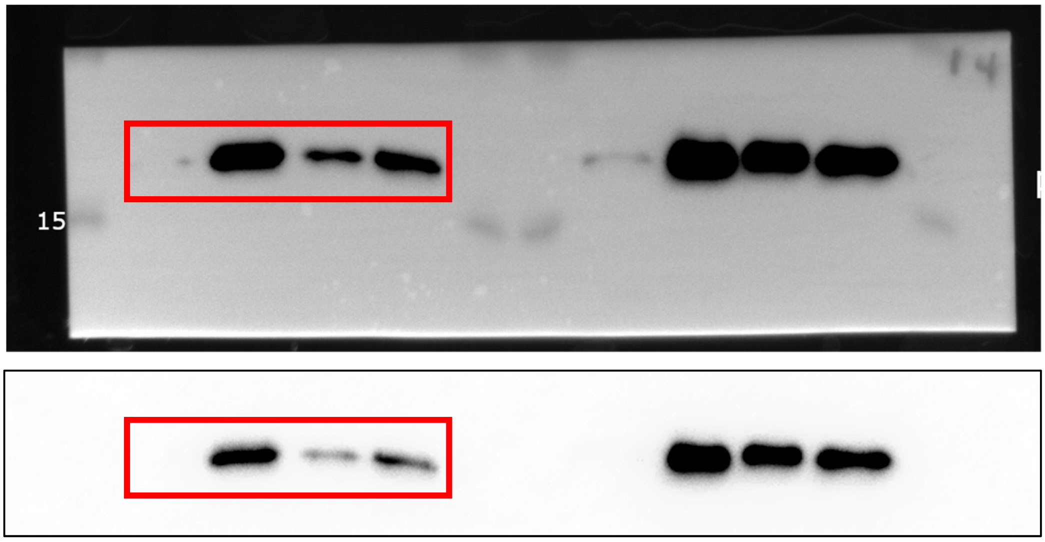

E6AP

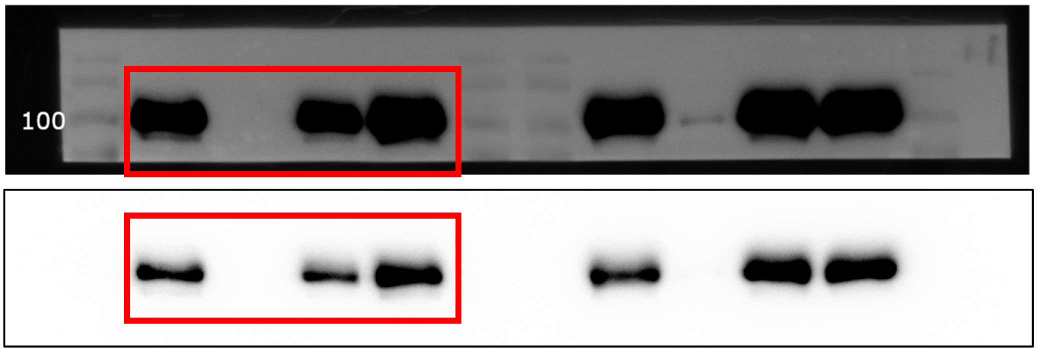

18E6

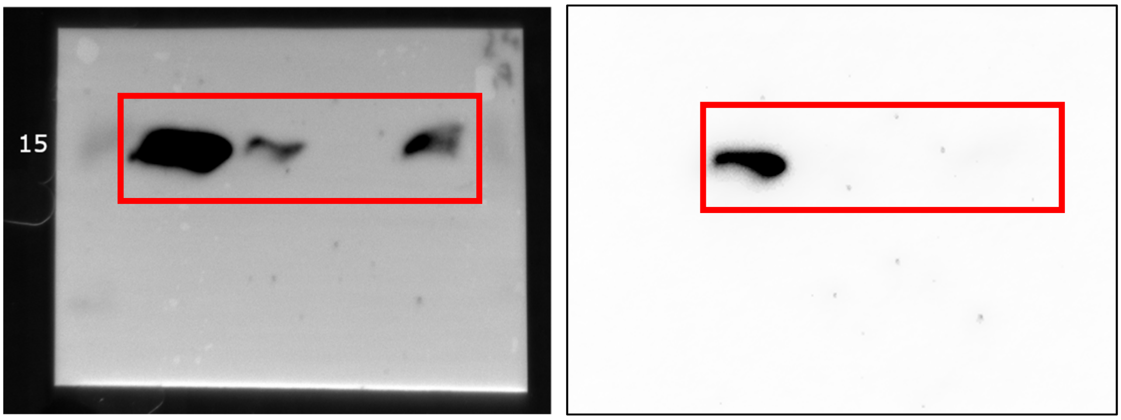

Fig 5A HeLa

18E7

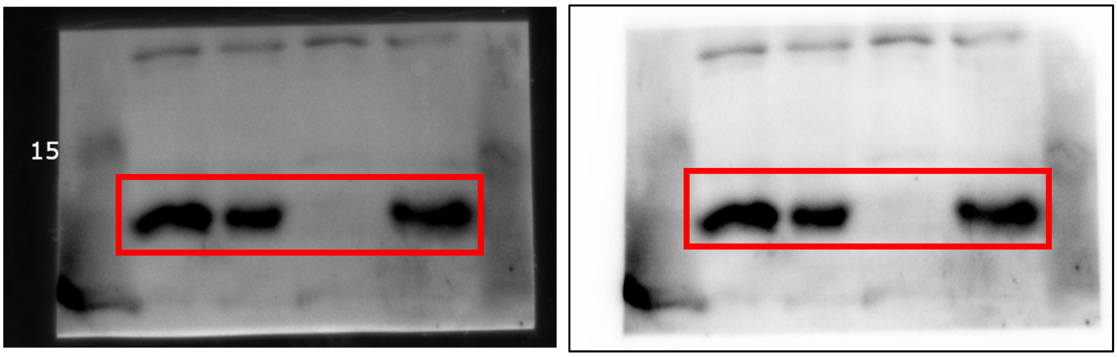

GAPDH

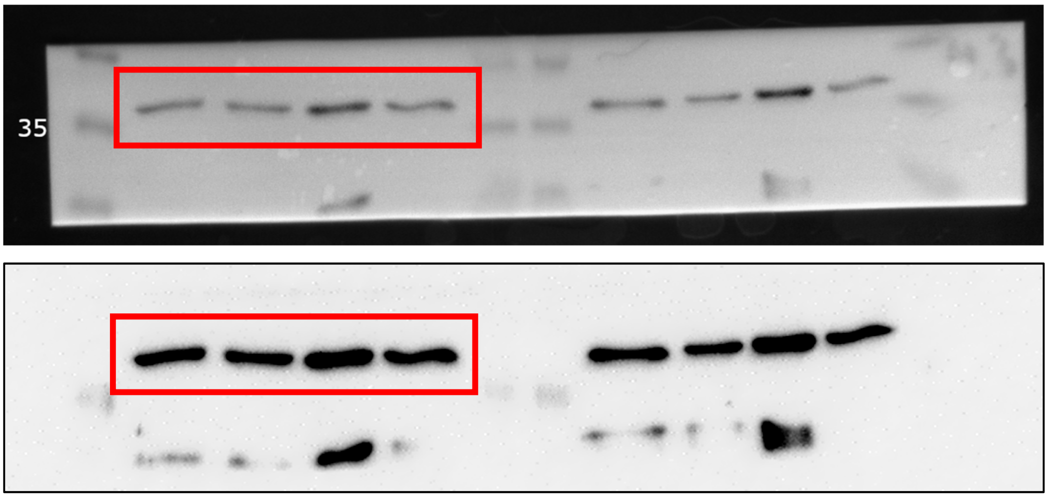

Fig 5A SiHa

p53

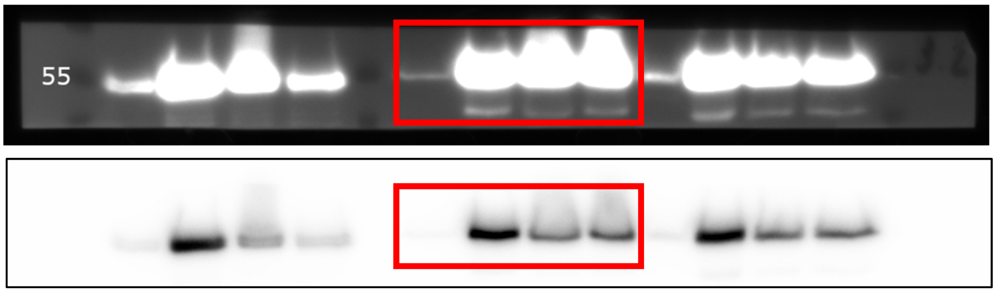

P-p53 Ser15

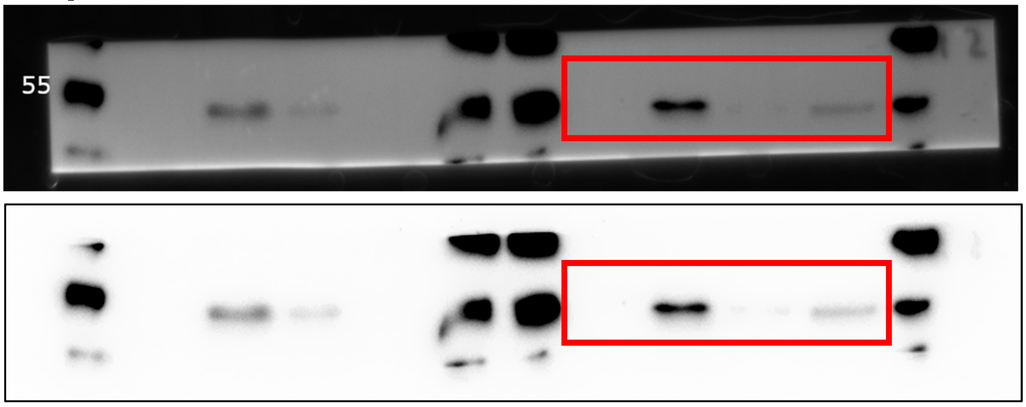

P-p53 Ser20

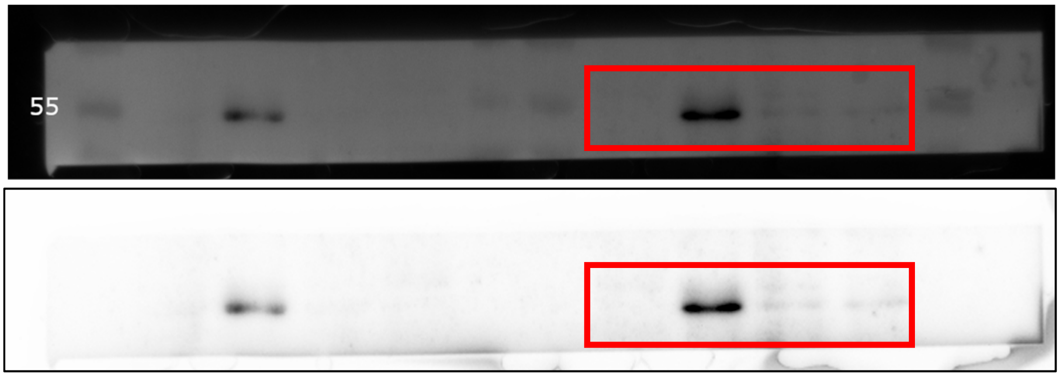

Ac-p53

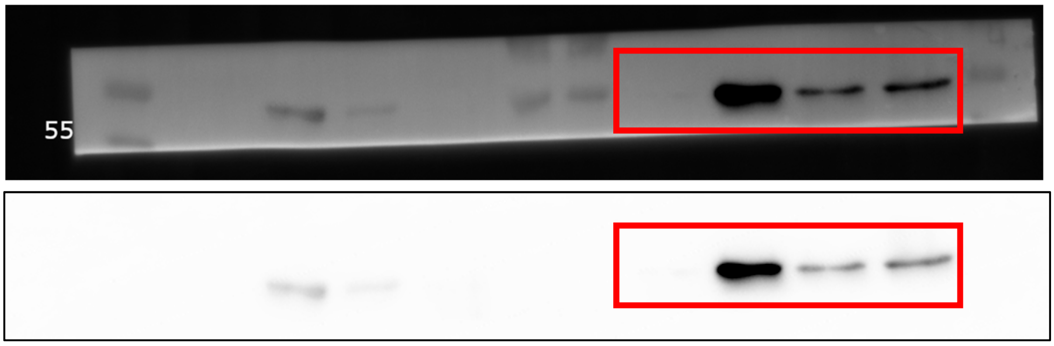

Fig 5A SiHa

p21

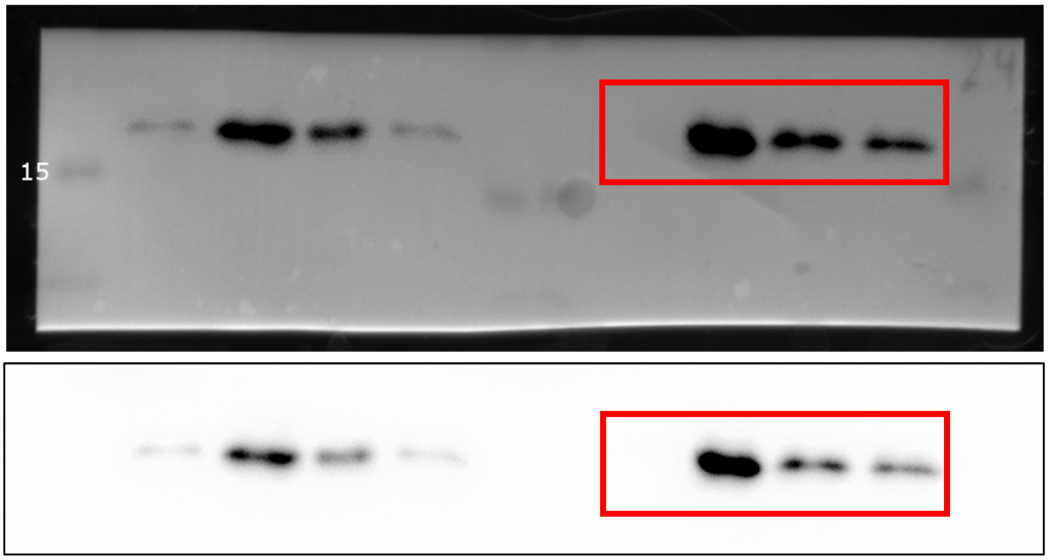

E6AP

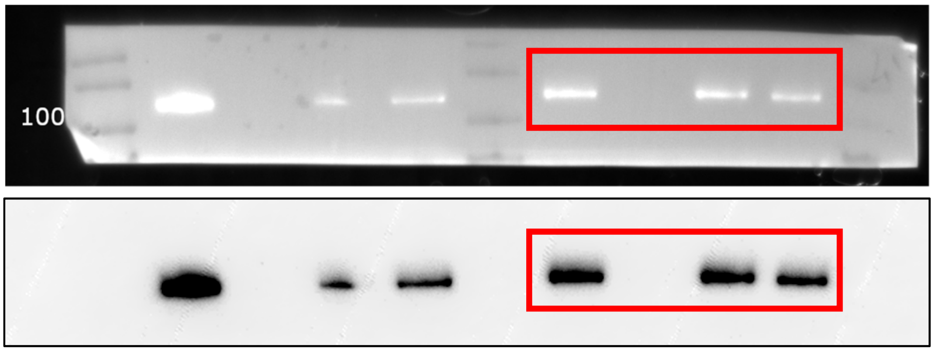

16E6

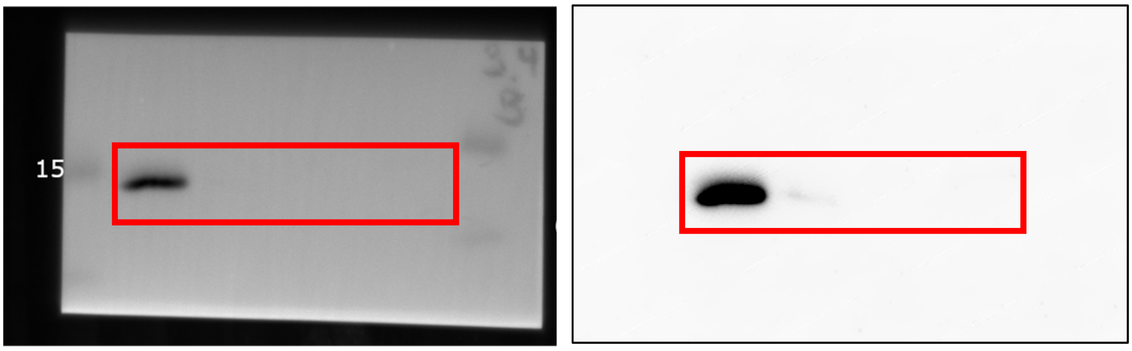

Fig 5A SiHa

16E7

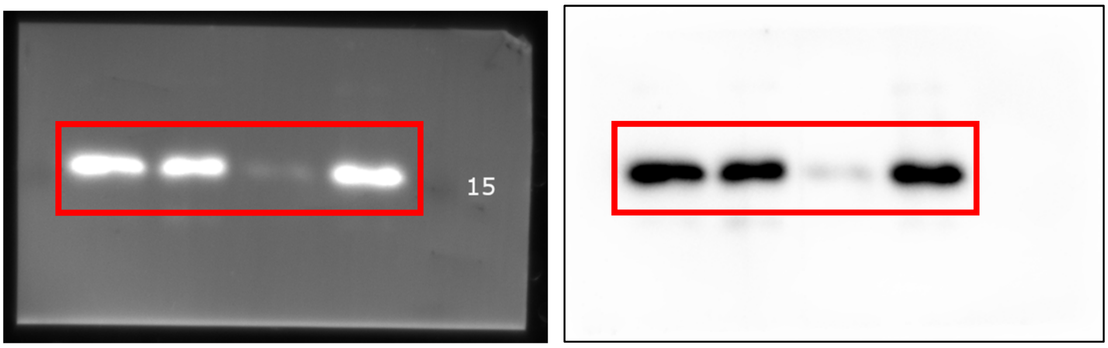

GAPDH

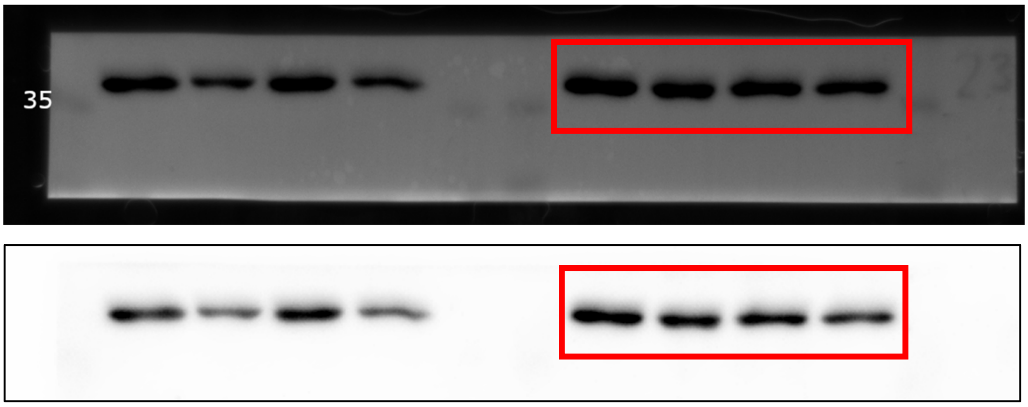

Fig 5C HeLa

p53

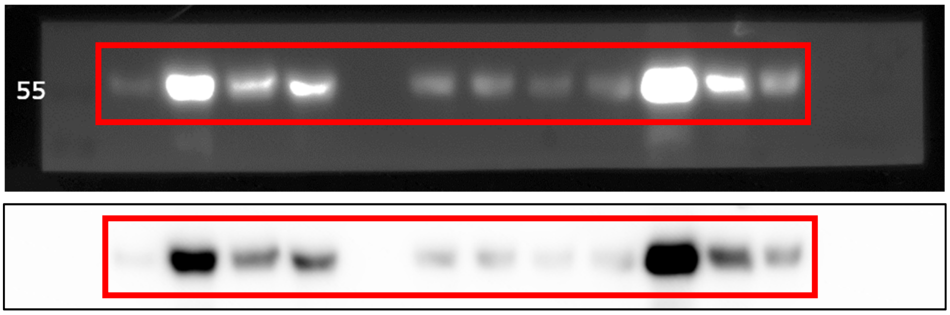

p21

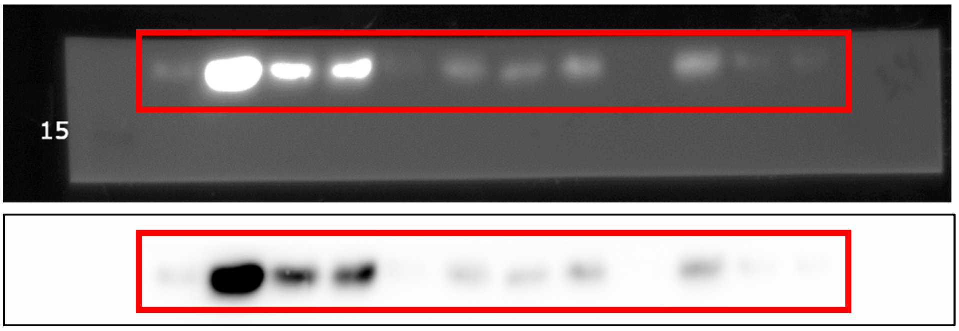

E6AP

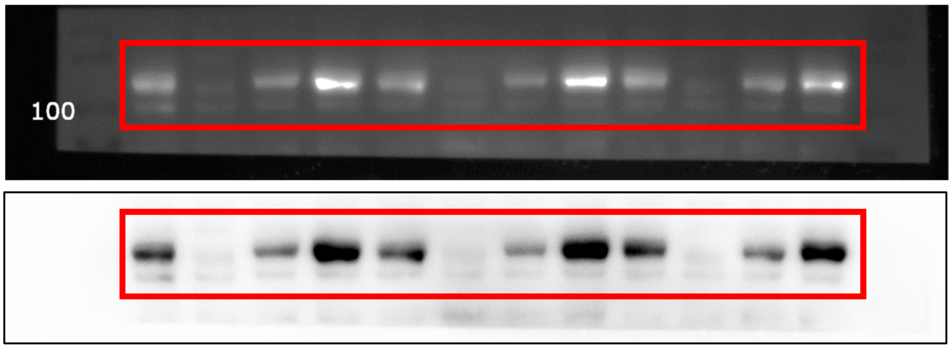

18E6

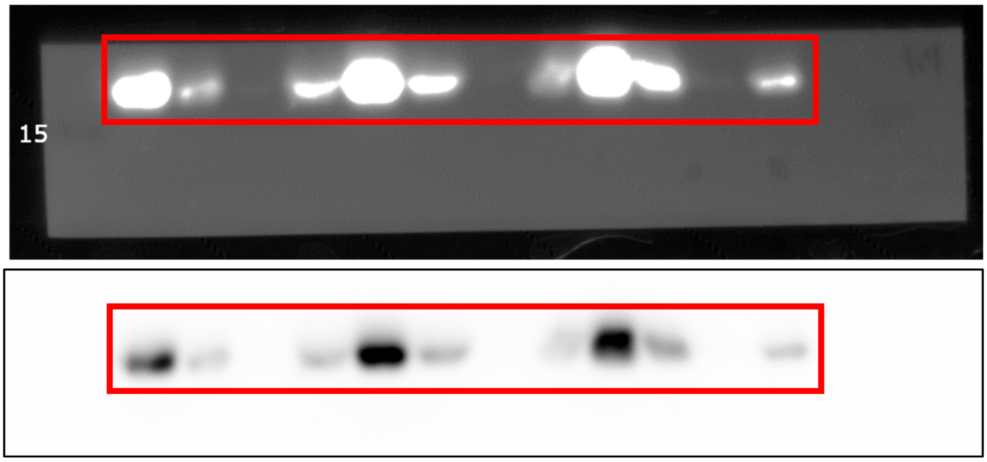

Fig 5C HeLa

18E7

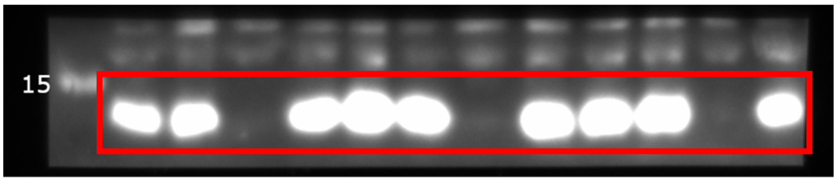

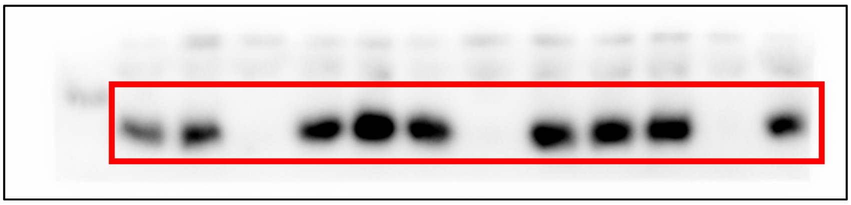

GAPDH

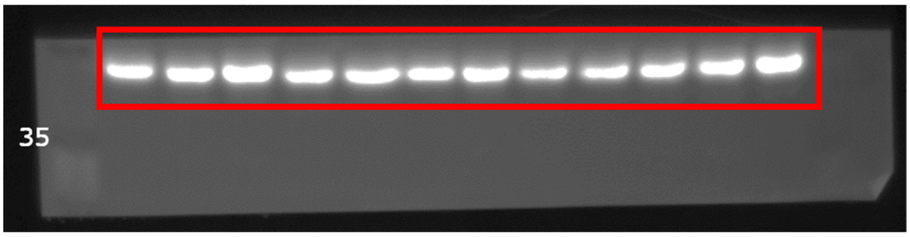

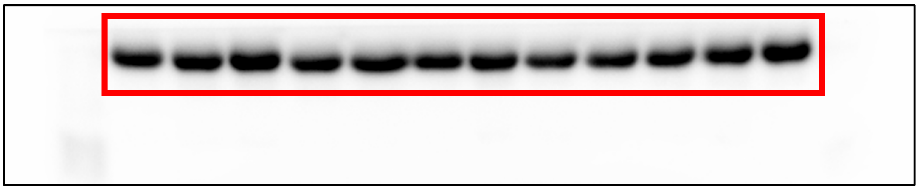

Fig 5C SiHa

p53

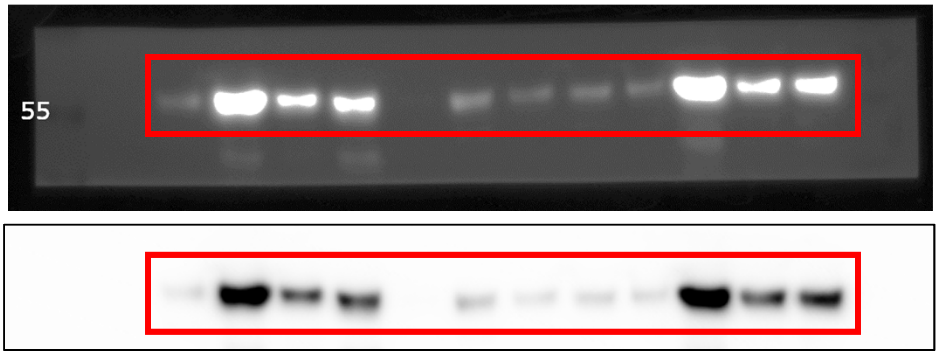

p21

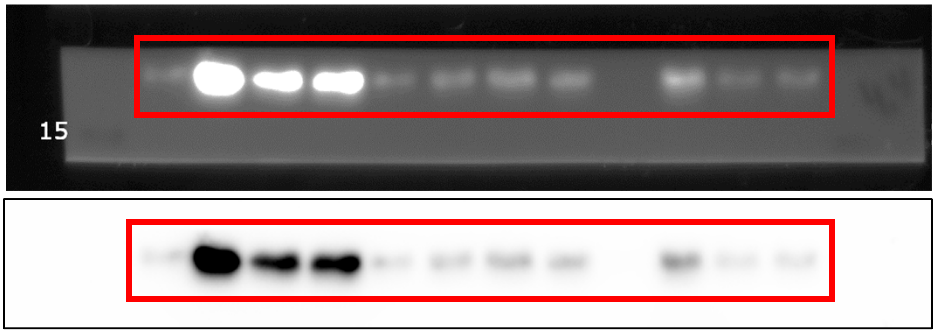

E6AP

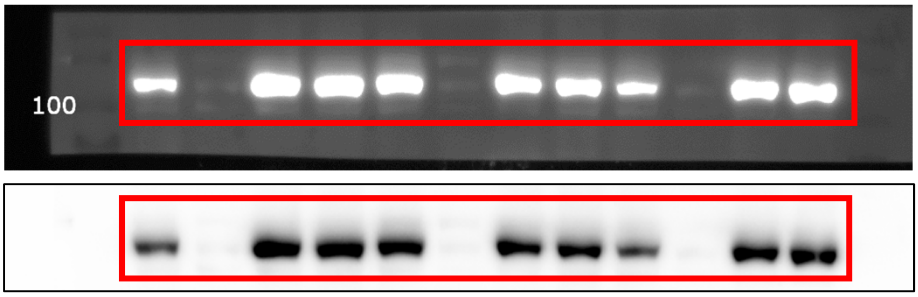

16E6

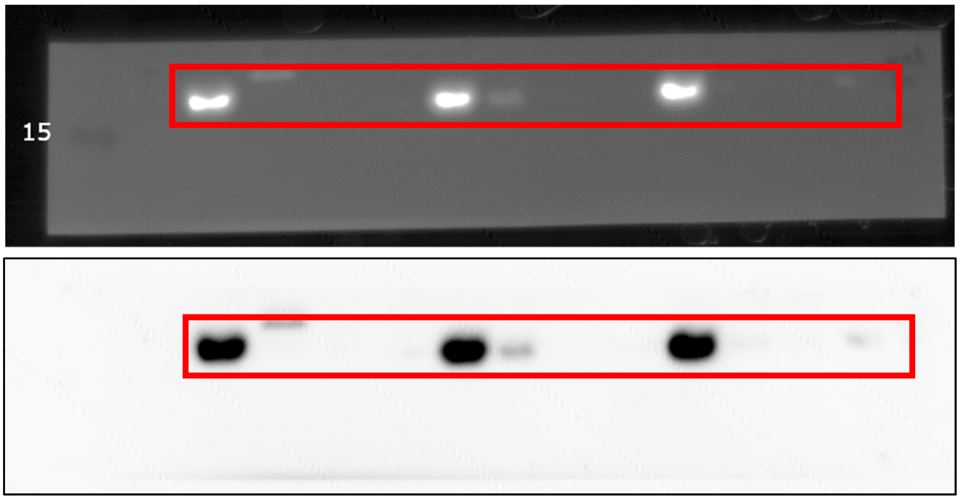

Fig 5C SiHa

16E7

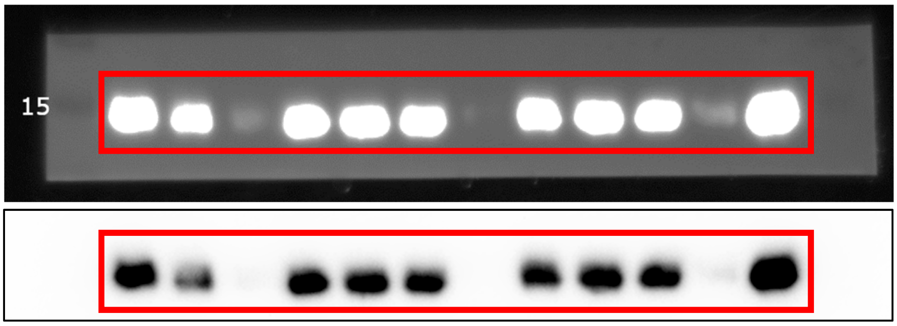

GAPDH

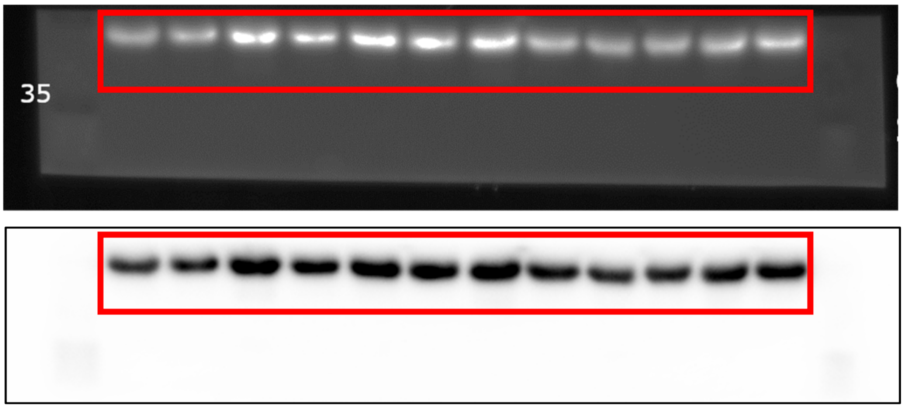

Fig 7A

p53

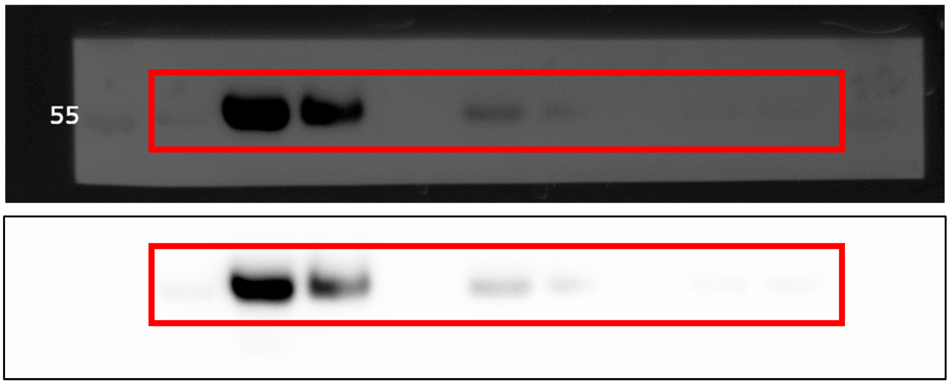

p21

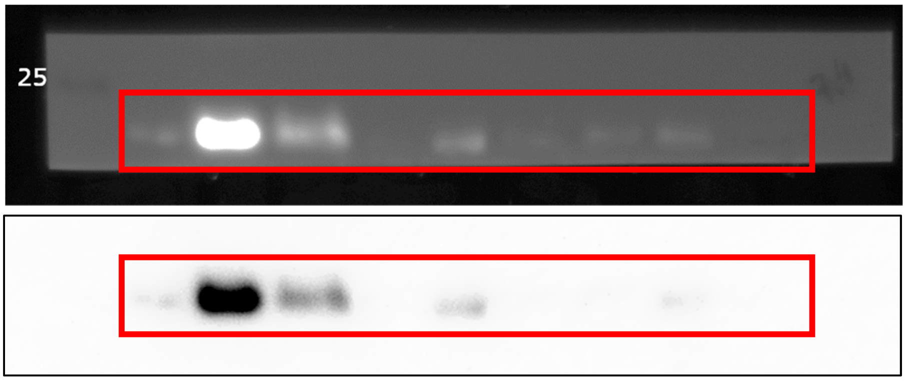

E6AP

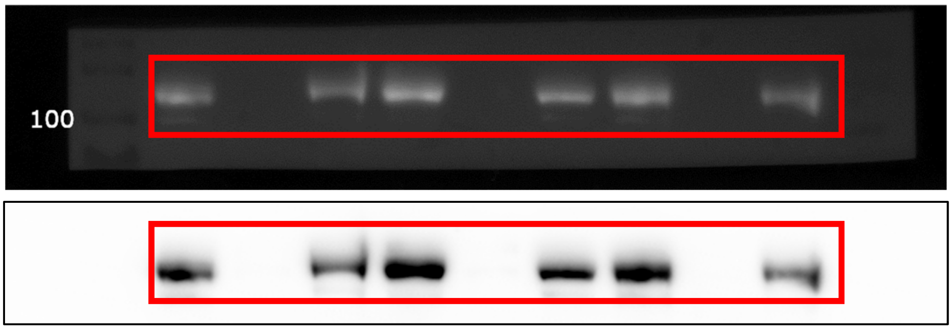

18E6

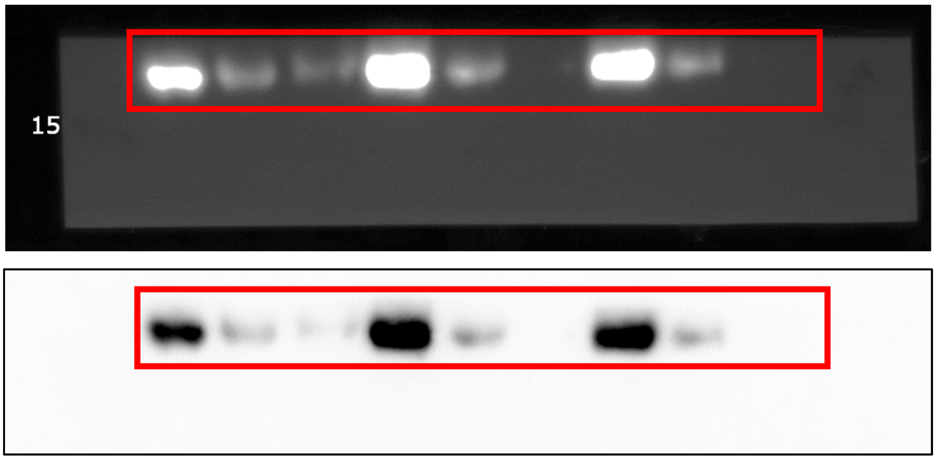

Fig 7A

18E7

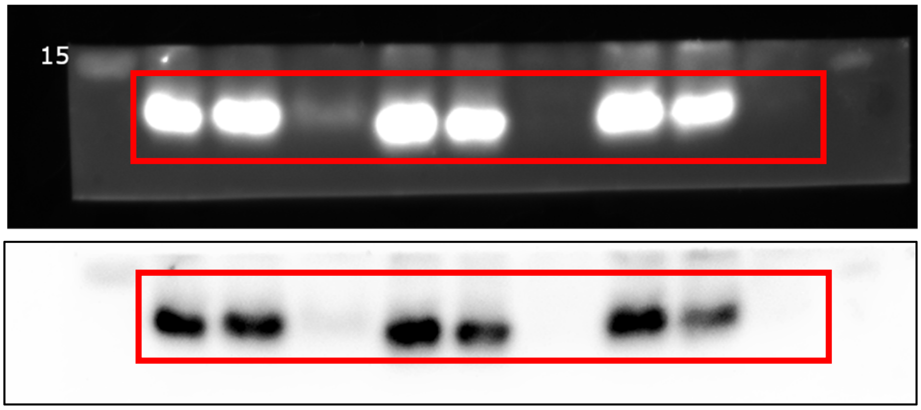

GAPDH

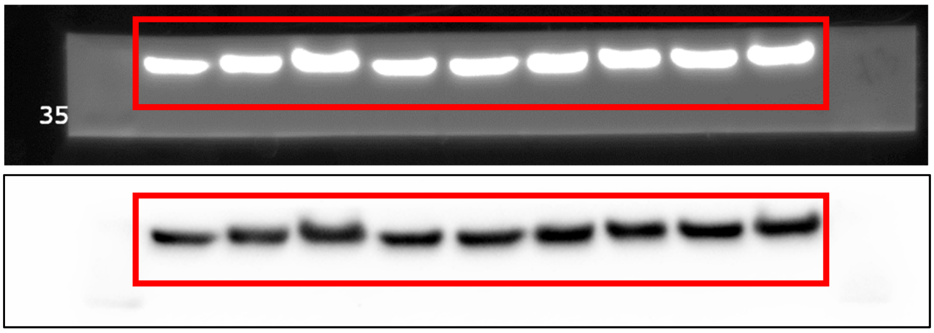

Fig 8A

P-pRb

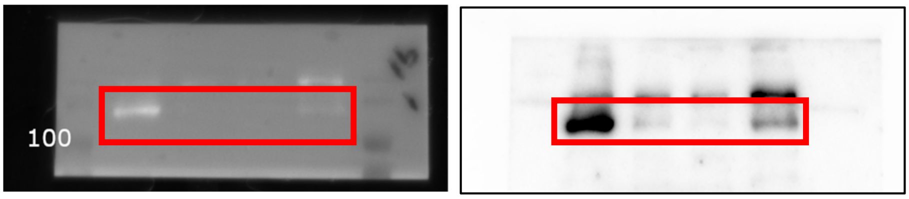

pRb

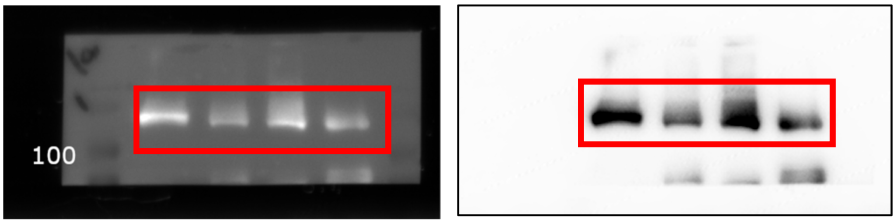

p130

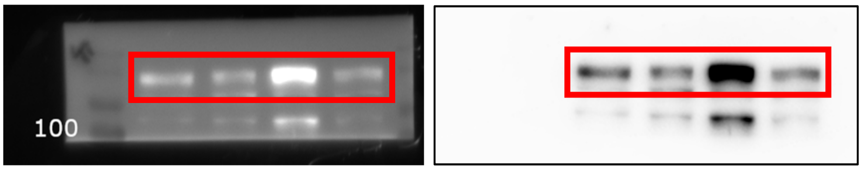

E6AP

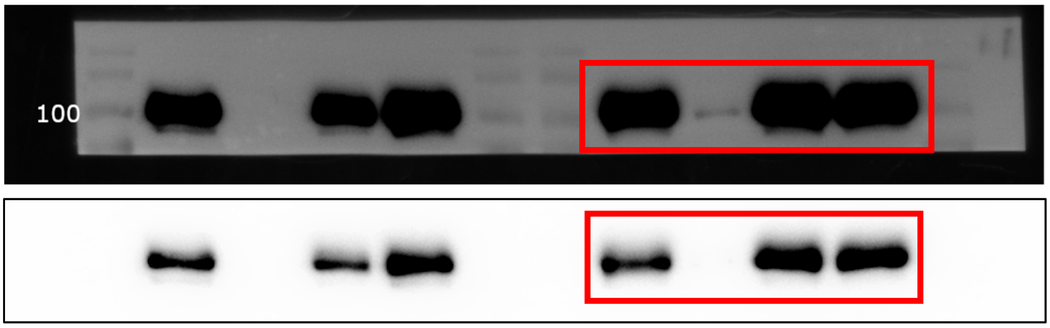

16E6

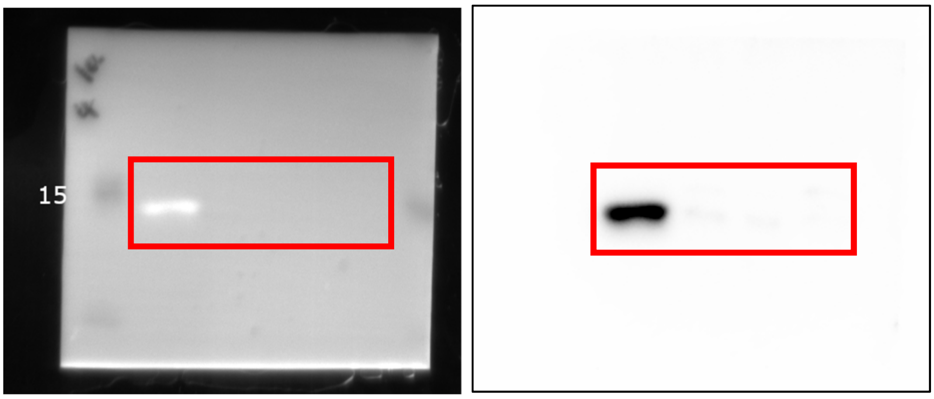

Fig 8A

16E7

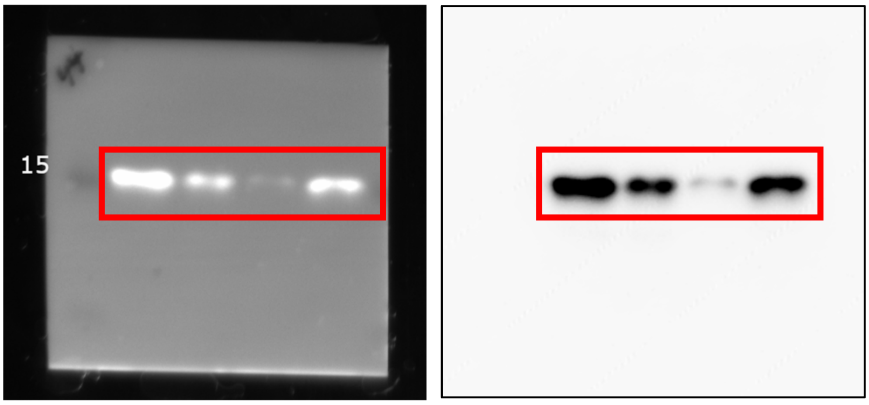

β-Actin

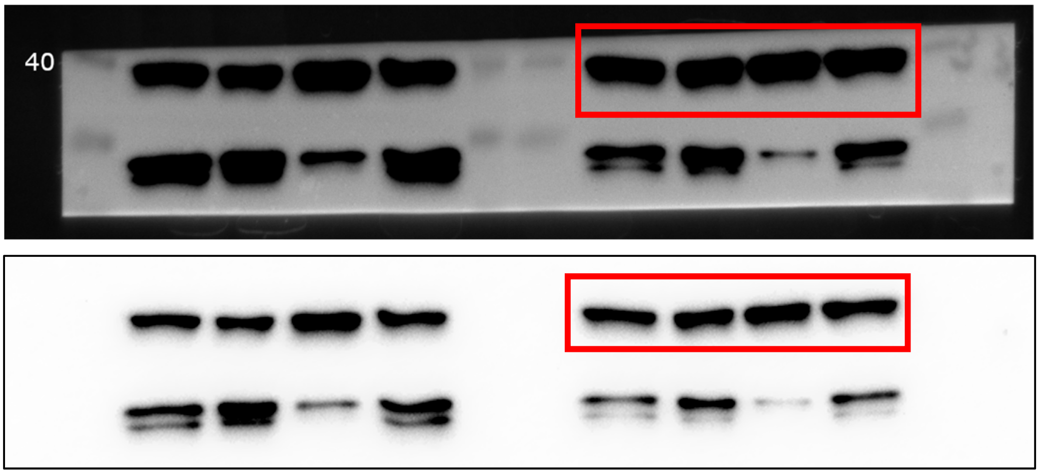

Fig 8B

P-pRb

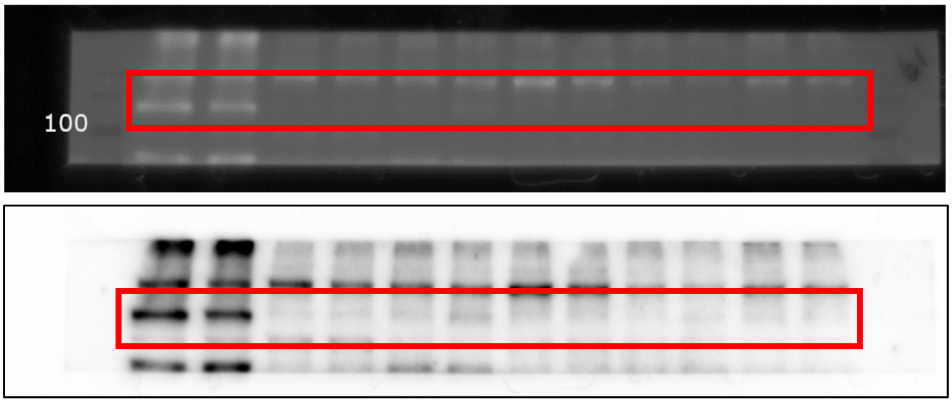

pRb

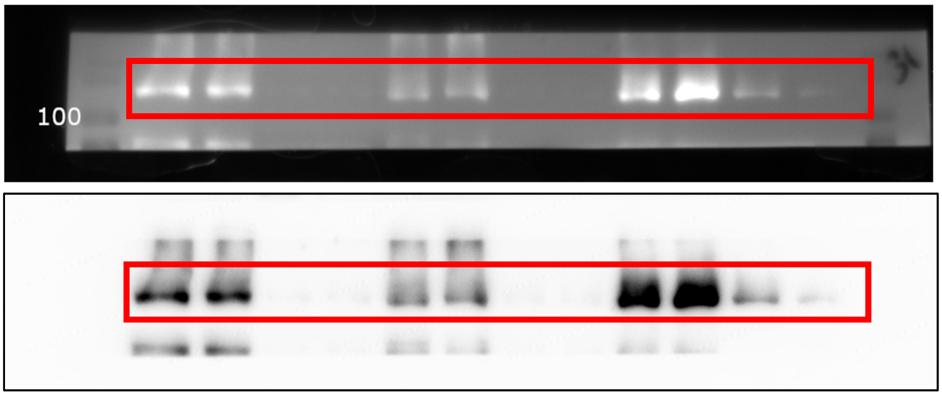

p130

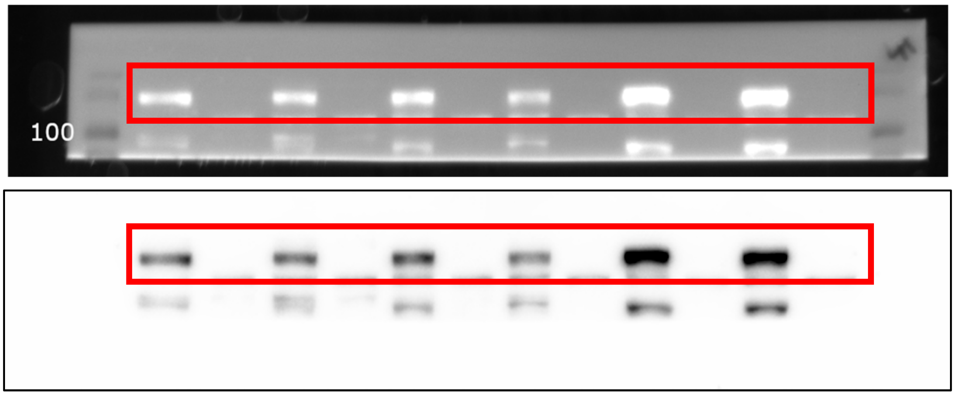

E6AP

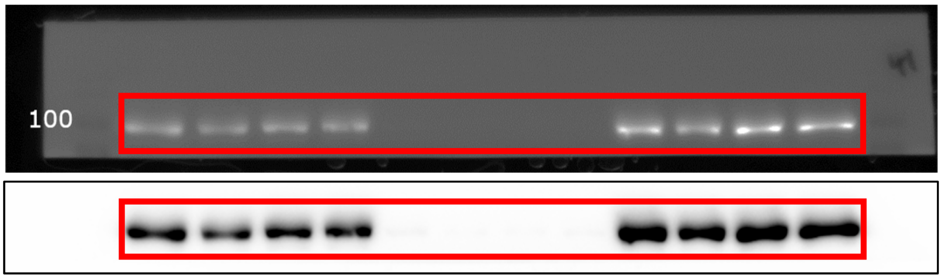

Fig 8B

16E6

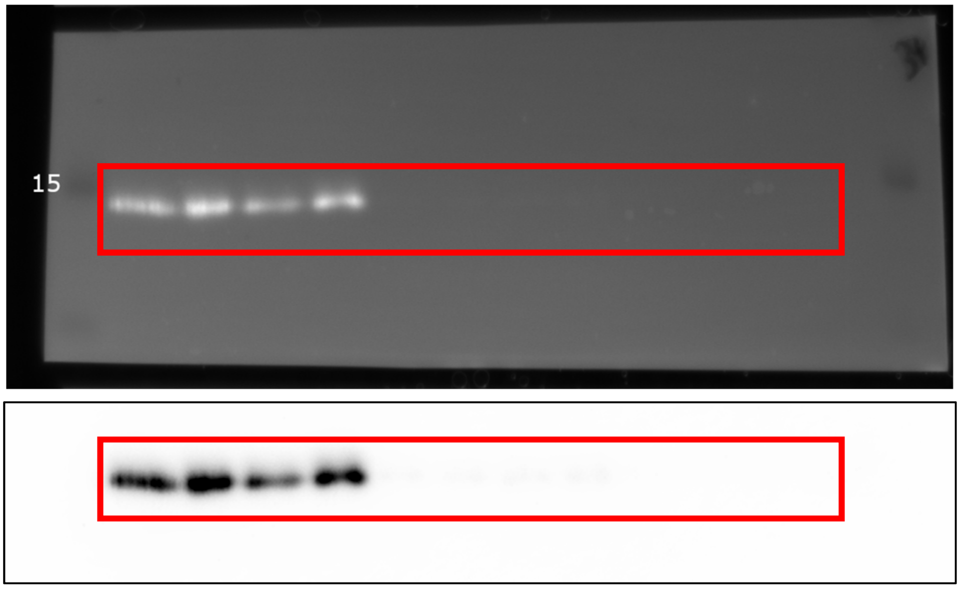

16E7

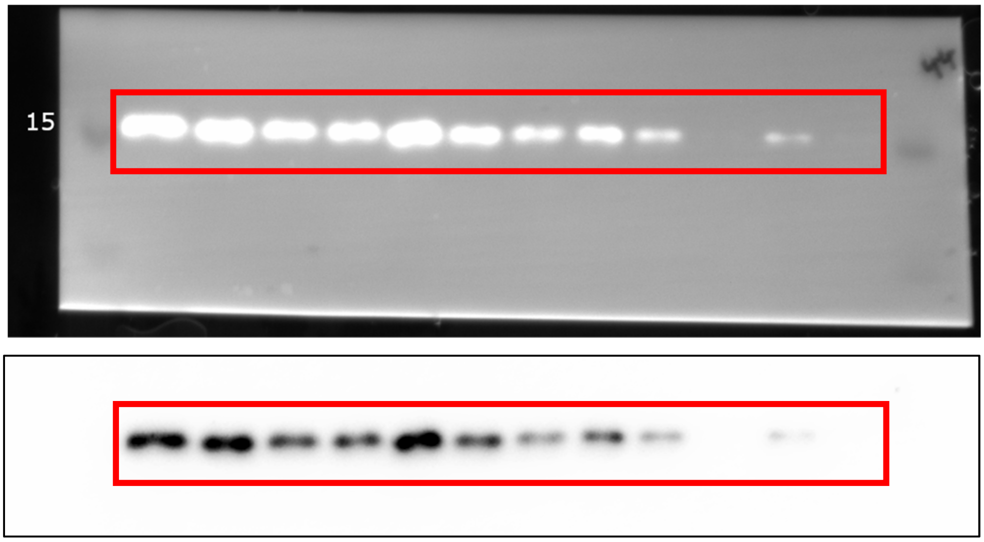

β-Actin

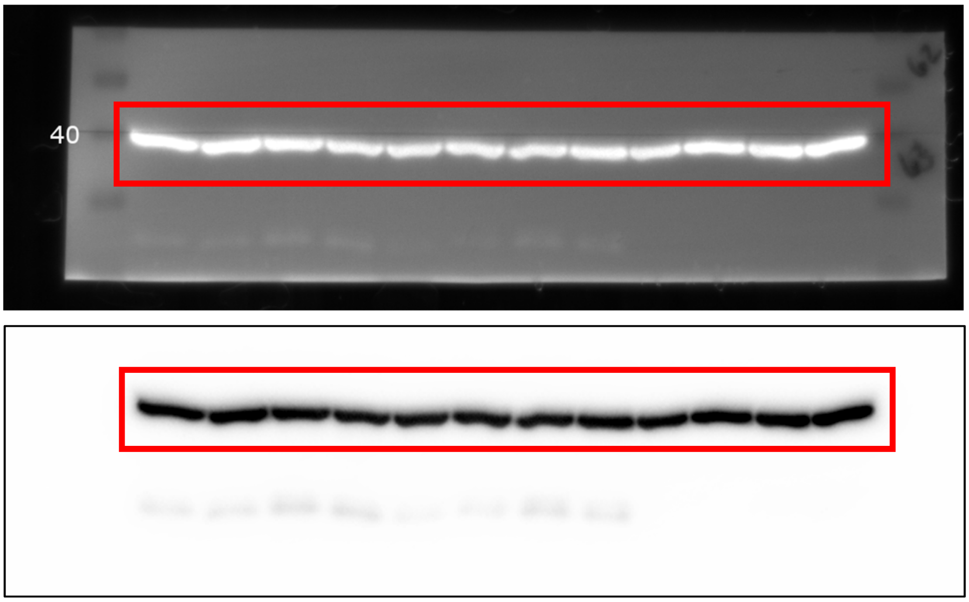

Fig 8D

P-pRb

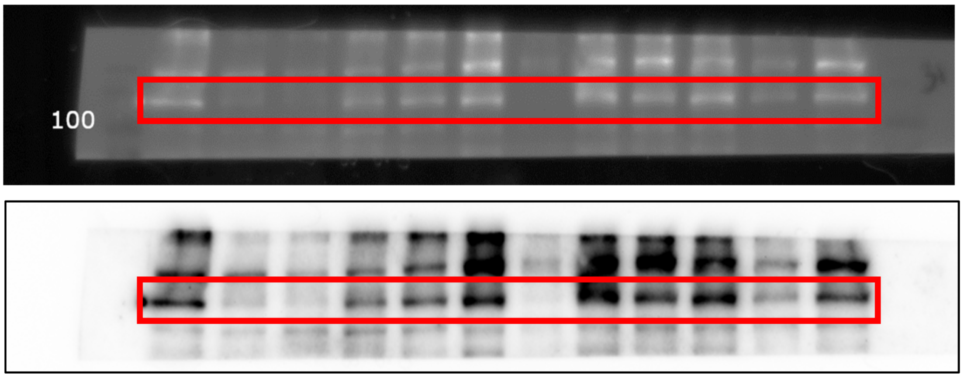

pRb

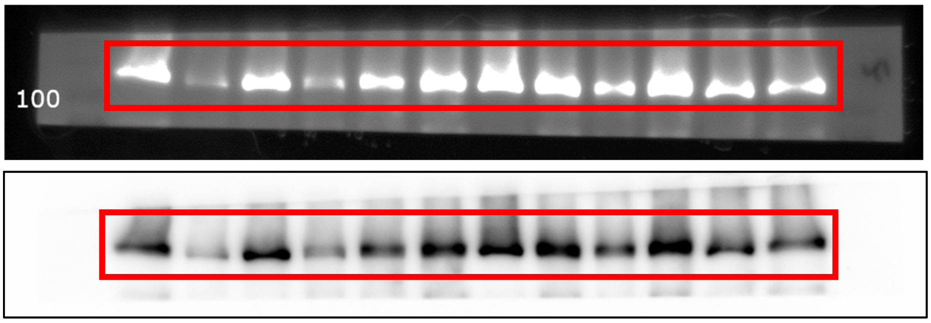

GAPDH

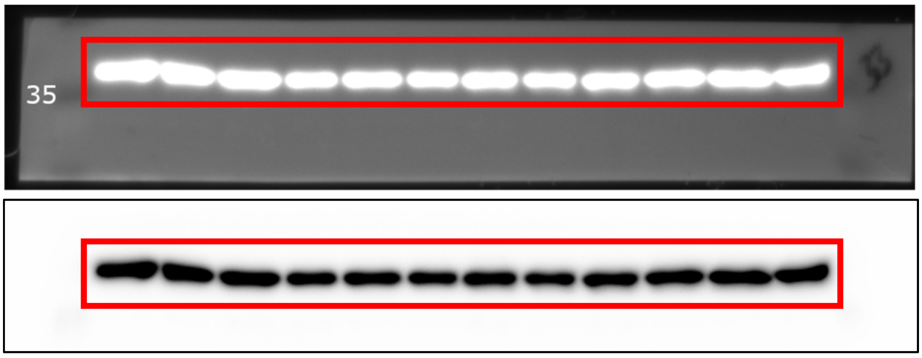

Supplement: S2 Raw images — For each blot, two images are presented: (1) a marker overlay image indicating molecular weight (kDa) positions based on a protein marker, and (2) the exposures of the blots used for the individual figures. Red boxes indicate the crops used for the figures. (PDF) [file ppat.1012914.s008.pdf]
