# Supplementary material for: E6AP is essential for the proliferation of HPV-positive cancer cells by preventing senescence
Source: PLoS Pathog. 2025 Feb 7;21(2):e1012914. doi: 10.1371/journal.ppat.1012914 (PMC11805377; doi:10.1371/journal.ppat.1012914)

S1B Fig, RKO

E6AP

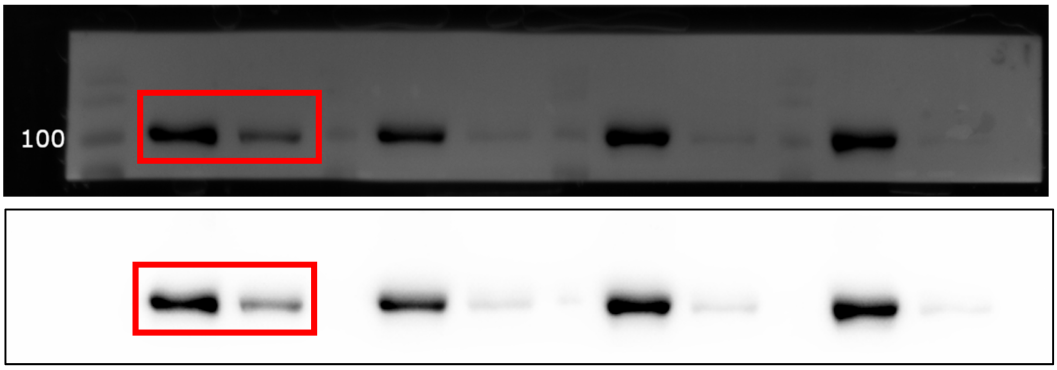

p53

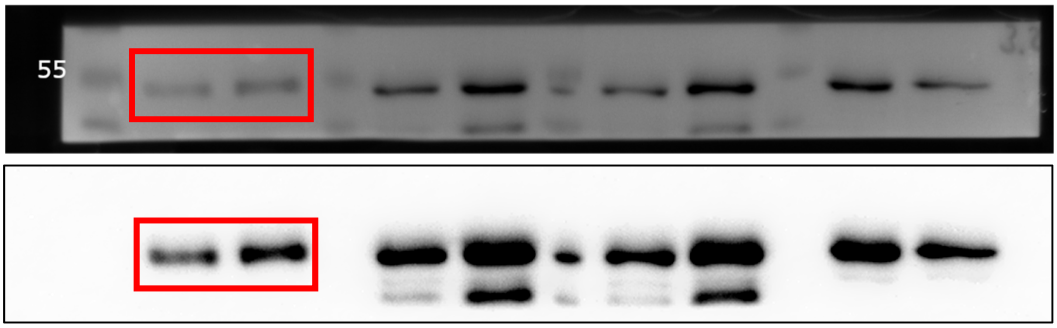

p21

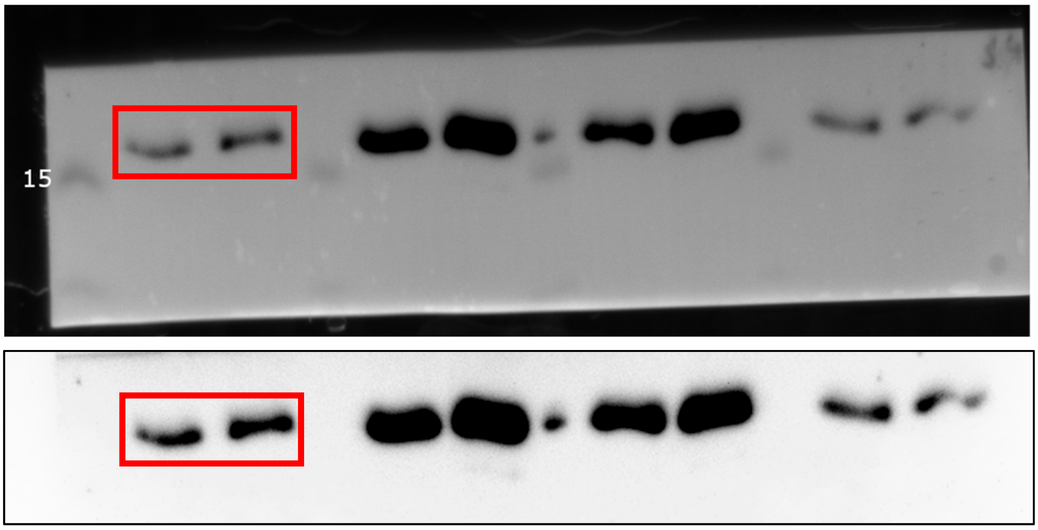

GAPDH

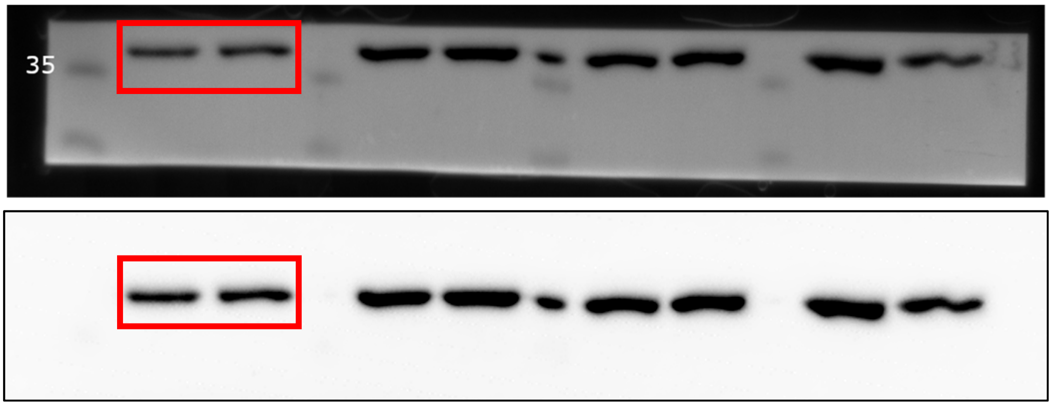

S1B Fig, HCT116

E6AP

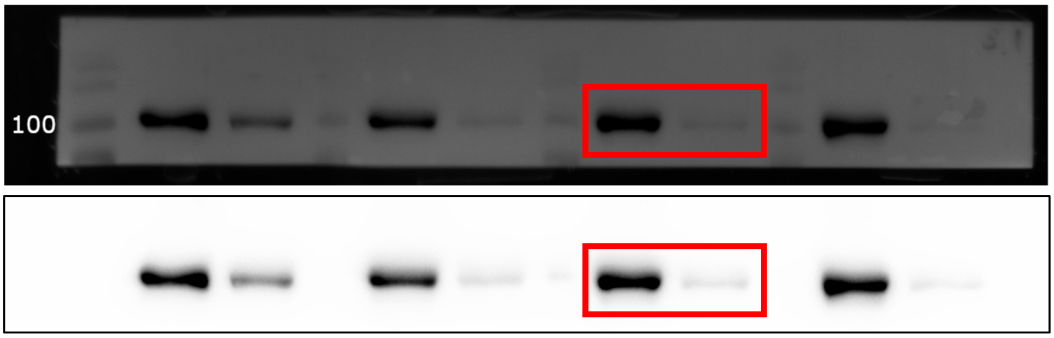

p53

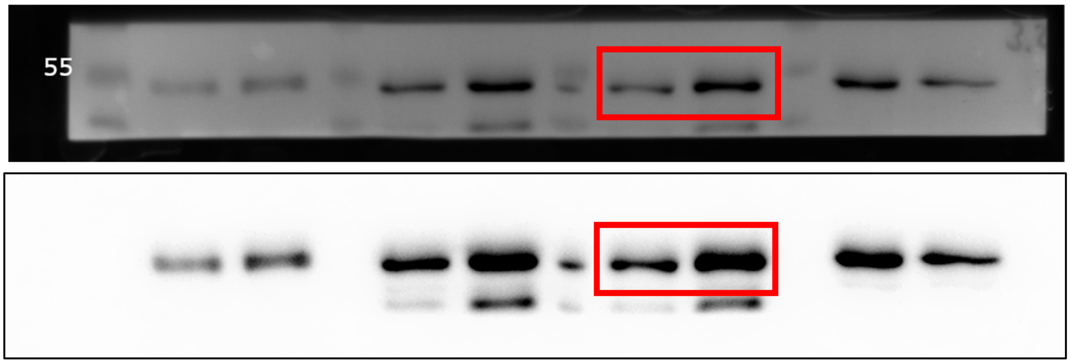

p21

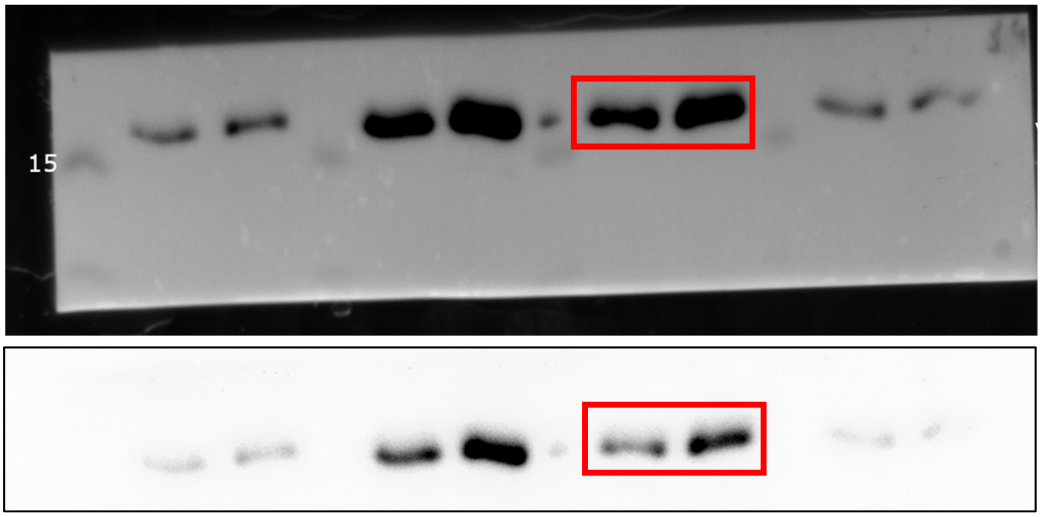

GAPDH

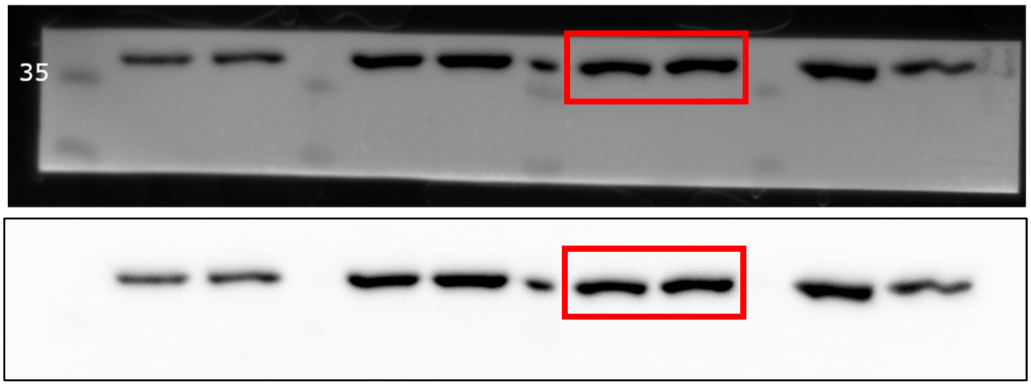

S1B Fig, U2OS

E6AP

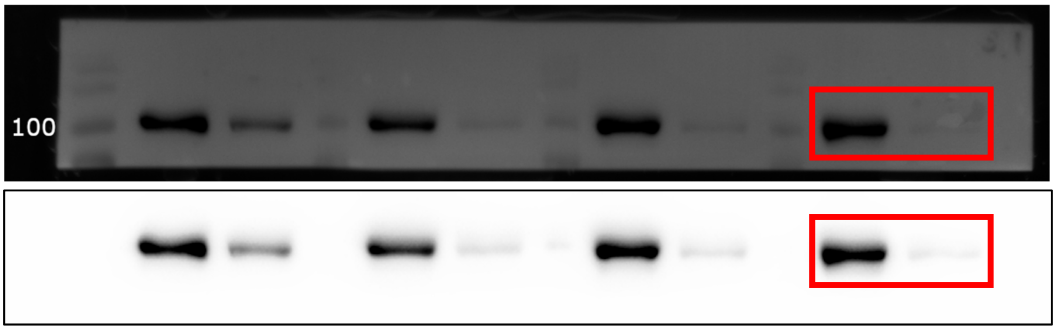

p53

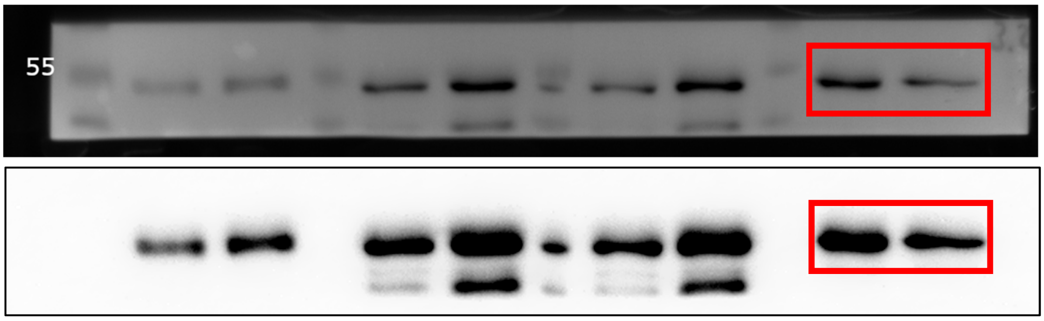

p21

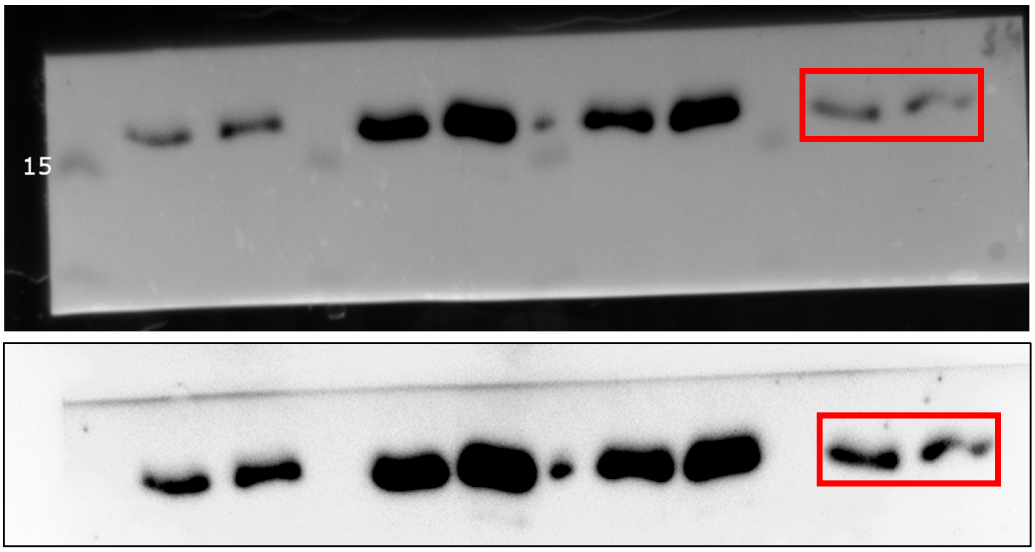

GAPDH

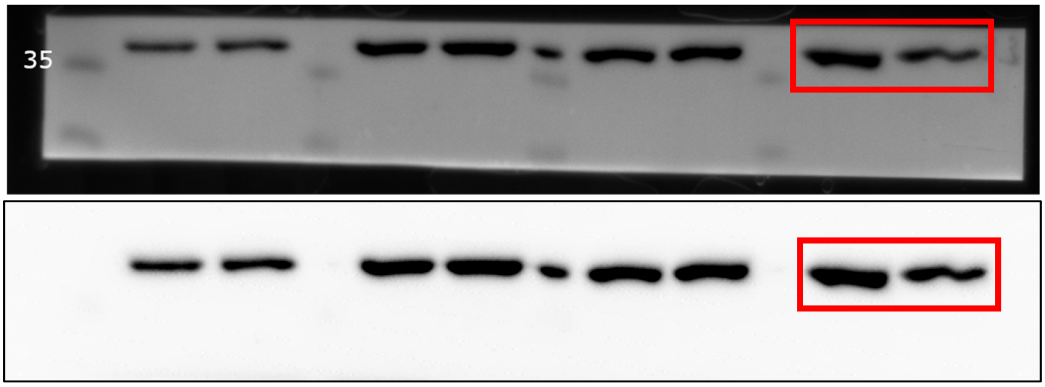

S2C Fig

E6AP

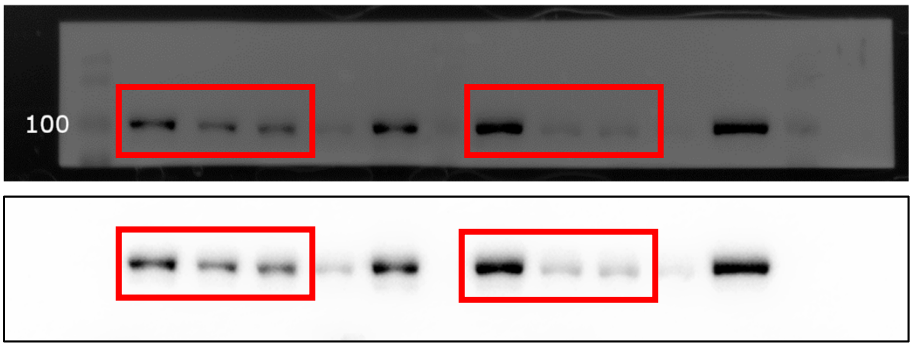

p53

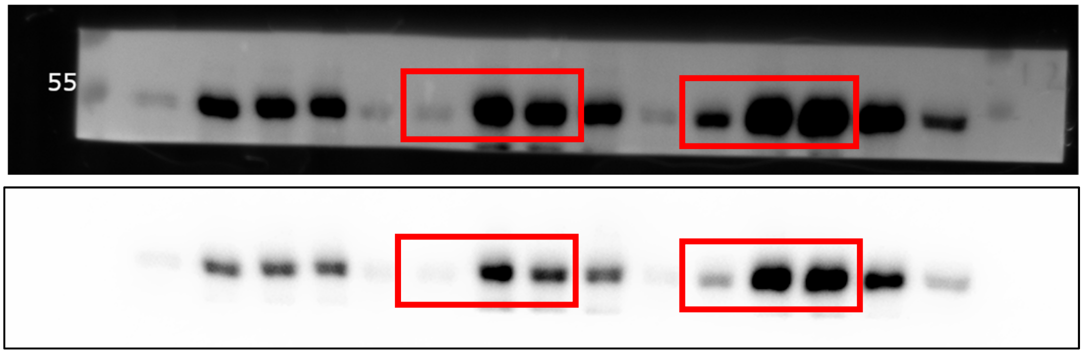

p21

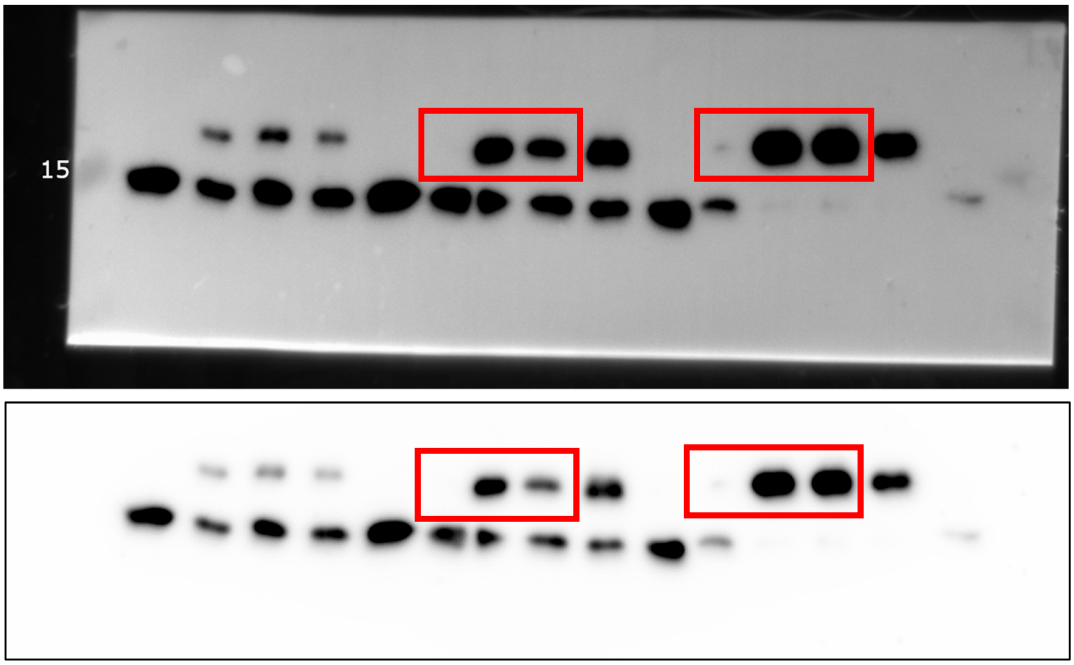

S2C Fig

18E6

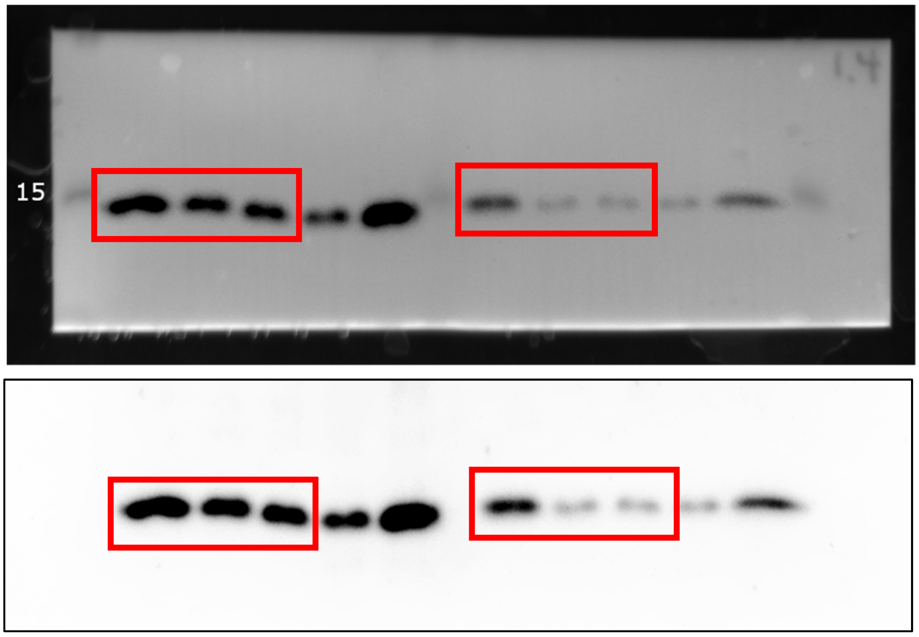

18E7

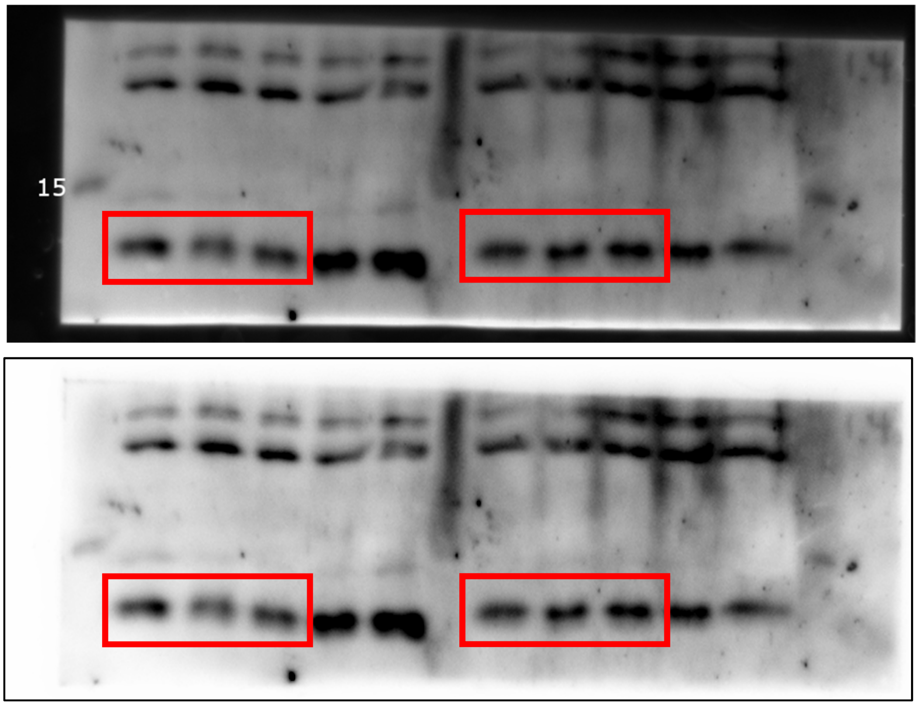

GAPDH

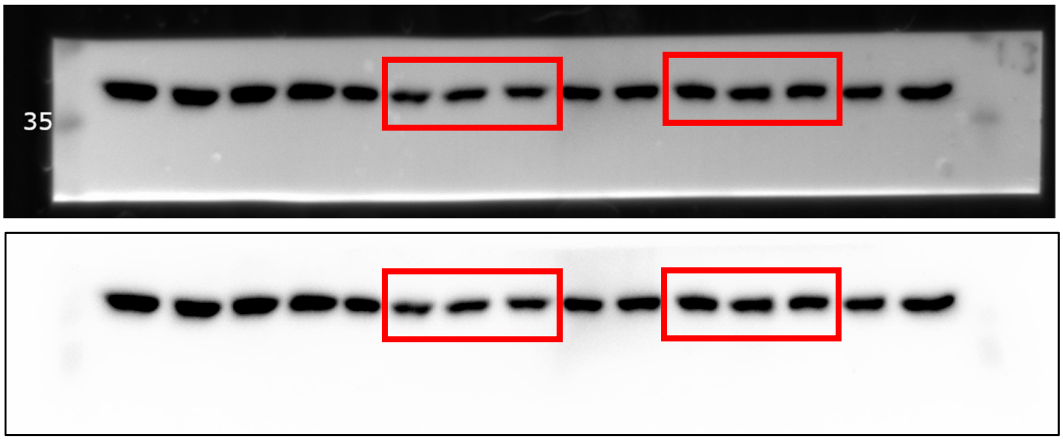

S3B Fig

P-pRb

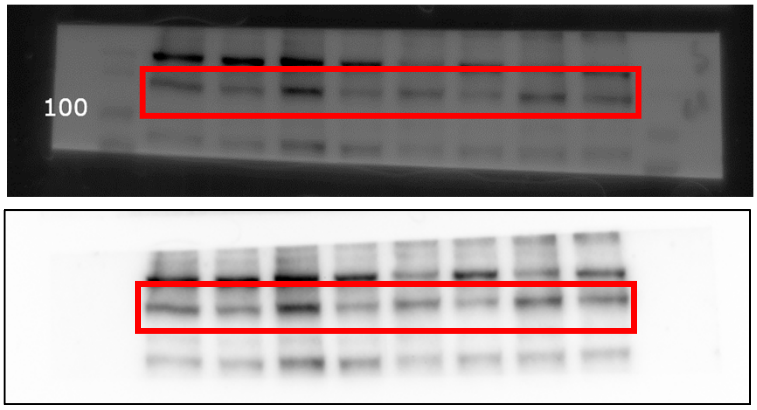

pRb

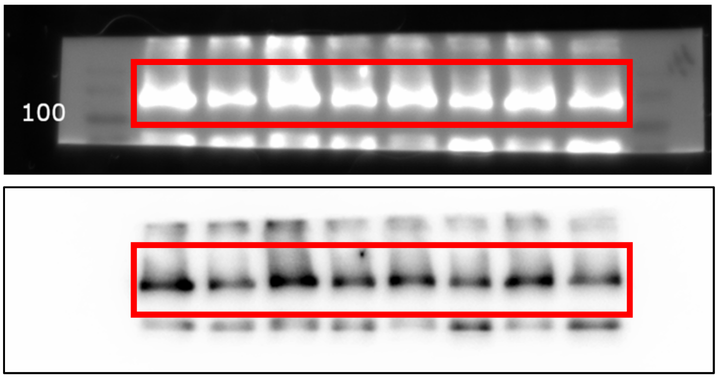

E6AP

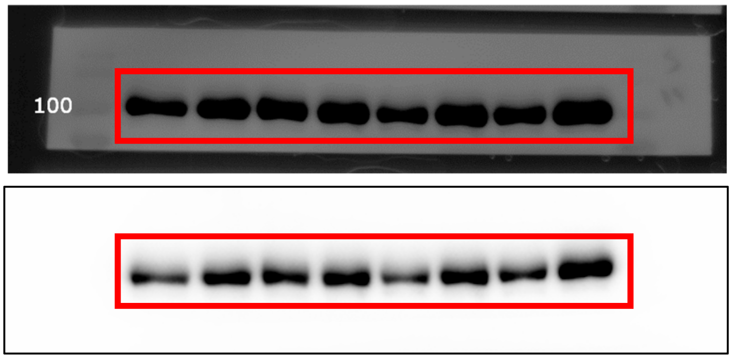

S3B Fig

16E6

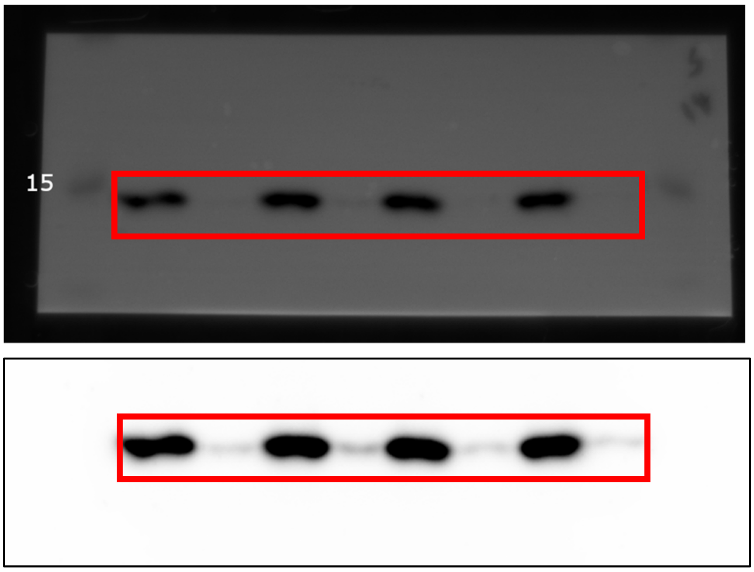

16E7

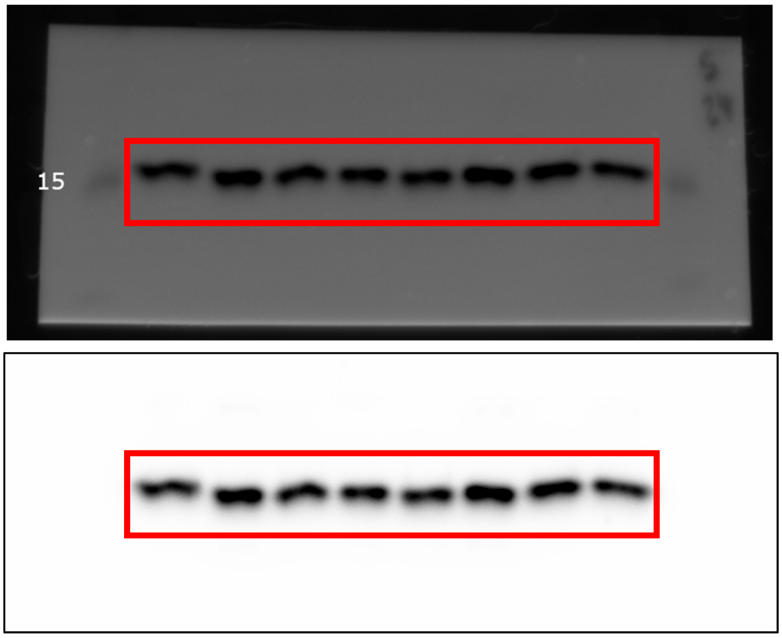

β-Actin

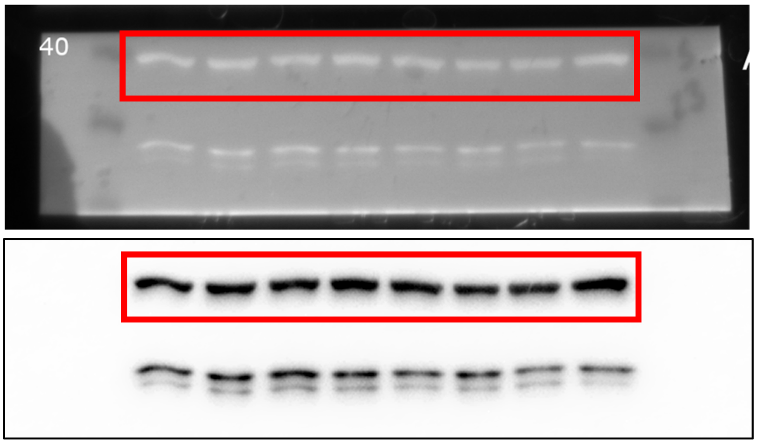

Supplement: S3 Raw images — For each blot, two images are presented: (1) a marker overlay image indicating molecular weight (kDa) positions based on a protein marker, and (2) the exposures of the blots used for the individual figures. Red boxes indicate the crops used for the figures. (PDF) [file ppat.1012914.s009.pdf]
